# Supplementary material for: Microfluidic space coding for multiplexed nucleic acid detection via CRISPR-Cas12a and recombinase polymerase amplification
Source: Nat Commun. 2022 Oct 29;13:6480. doi: 10.1038/s41467-022-34086-y (PMC9617605; doi:10.1038/s41467-022-34086-y)
Supplement: Supplementary file 1 — Supplementary Information [file 41467_2022_34086_MOESM1_ESM.pdf]

Microfluidic space coding for multiplexed nucleic acid detection via CRISPR-Cas12a and recombinase polymerase amplification

Zhichen Xu[a, b, #], Dongjuan Chen[c, #], Tao Li[a, b], Jiayu Yan[a, d], Jiang Zhu[a, b], Ting He[a, b], Rui Hu[a, b], Ying Li[a, b, *], Yunhuang Yang[a, b], Maili Liu[a, b]

[a] State Key Laboratory of Magnetic Resonance and Atomic Molecular Physics, National Centre for Magnetic Resonance in Wuhan, Wuhan Institute of Physics and Mathematics, Innovation Academy for Precision Measurement Science and Technology - Wuhan National Laboratory for Optoelectronics, Chinese Academy of Sciences, Wuhan 430071, China

[b] University of Chinese Academy of Sciences, Beijing 10049, China

[c] Department of Laboratory Medicine, Maternal and Child Health Hospital of Hubei Province, Tongji Medical College, Huazhong University of Science and Technology, Wuhan 430070, China

[d] School of Physical Education, China University of Geosciences, Wuhan 430074, China

#These authors contributed equally to this work.

* Corresponding author:

Email: liying@wipm.ac.cn

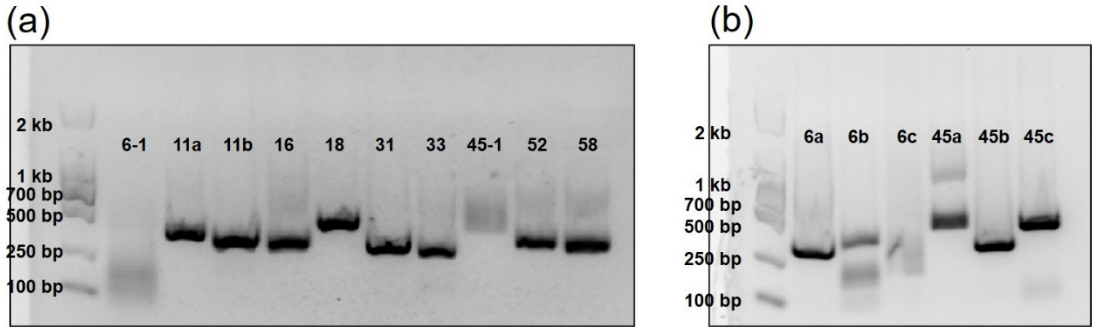

Supplementary Fig. 1 Evaluation of the RPA primers. (a) Agarose gel results showing the RPA products of the Round 1 primers for the 9 HPV subtypes. (b) Agarose gel results showing the RPA products of the Round 2 primers for HPV-6 and -45. Each experiment was repeated at least two times independently.

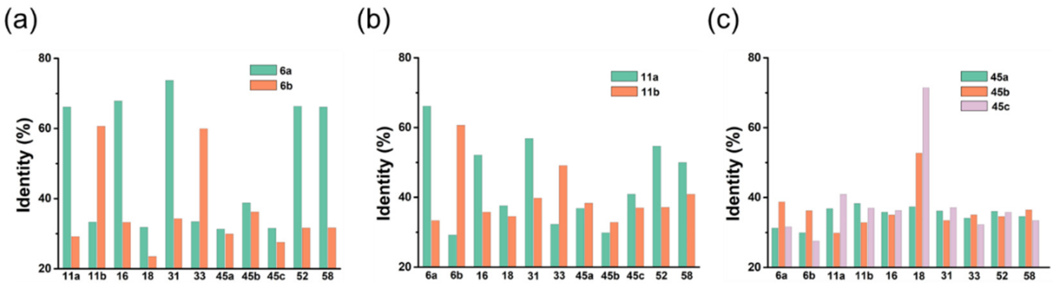

Supplementary Fig. 2 Primer screening by comparison the sequences of different amplification region. (a) Analysis of the sequence similarity between the amplification region of Primer HPV-6a and -6b and the other designed primers. Source data are provided as a Source Data file. (b) Analysis of the sequence similarity between the amplification region of Primer HPV-11a and -11b and the other designed primers. Source data are provided as a Source Data file. (c) Analysis of the sequence similarity between the amplification region of Primer HPV-45a, -45b and -45c and the other designed primers. Source data are provided as a Source Data file.

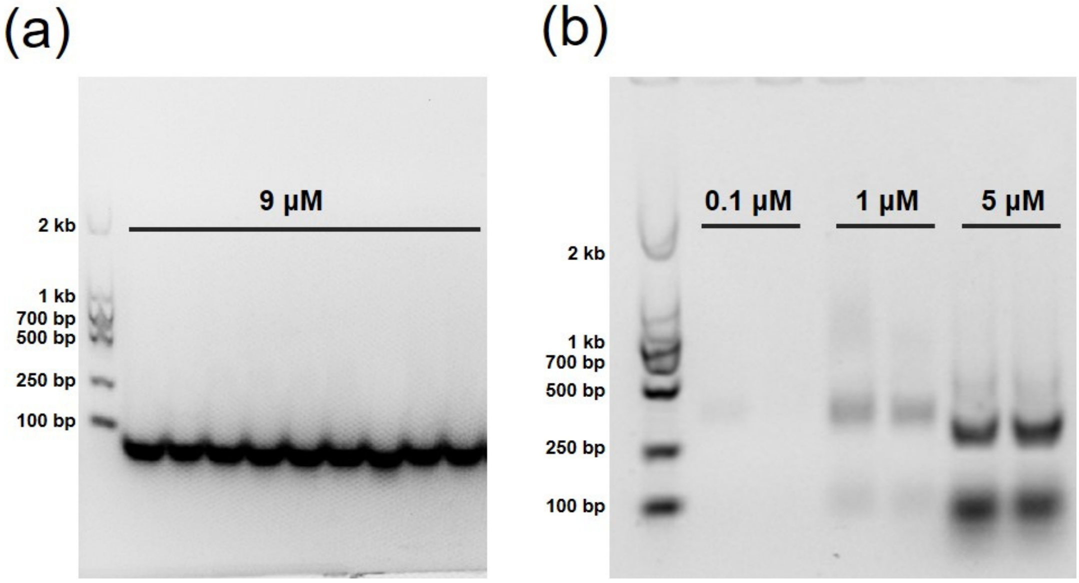

Supplementary Fig. 3 Optimization of the primer pool concentration. (a) Agarose gel results showing the RPA products of the round 1 primer pool (1 μM for each pair, and 9 μM in total). (b) Agarose gel results showing the RPA products of the round 2 primer pool (0.1, 1, and 5 μM in total for the 9 primers). Each experiment was repeated at least two times independently.

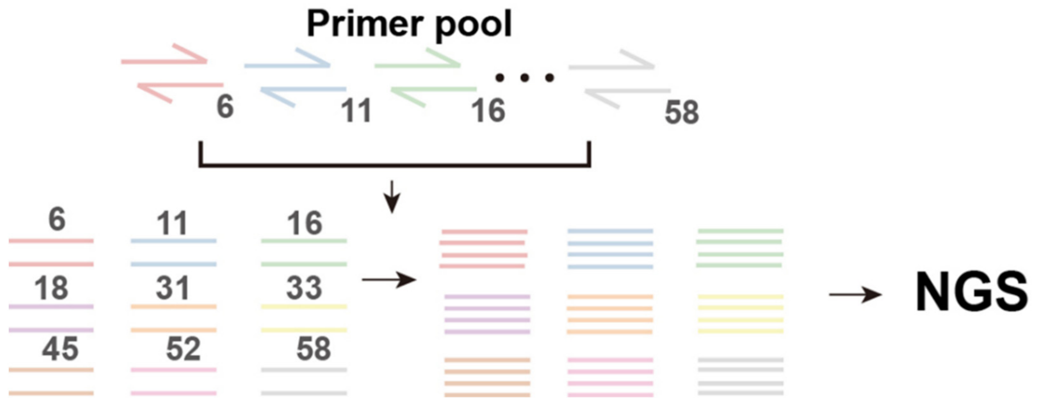

Supplementary Fig. 4 A brief workflow of NGS analysis of the RPA amplicons.

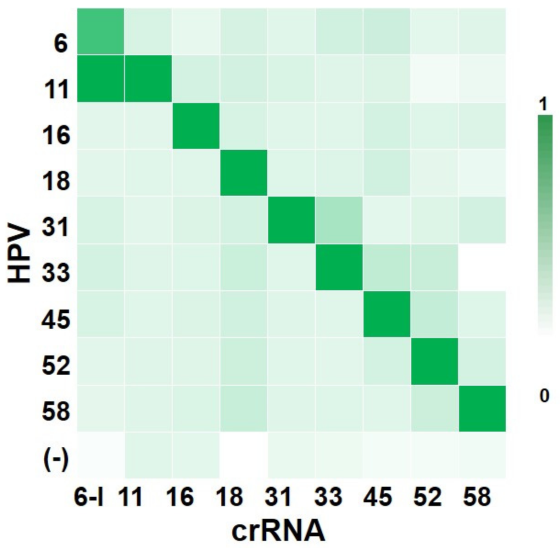

Supplementary Fig. 5 A matrix-based assay for testing the cross-reactivity of the nine crRNAs initially designed against the nine HPV subtypes. The results displayed that the initial crRNA of HPV-6 (crRNA 6-I) had cross-talk with the plasmid of HPV-11. Source data are provided as a Source Data file.

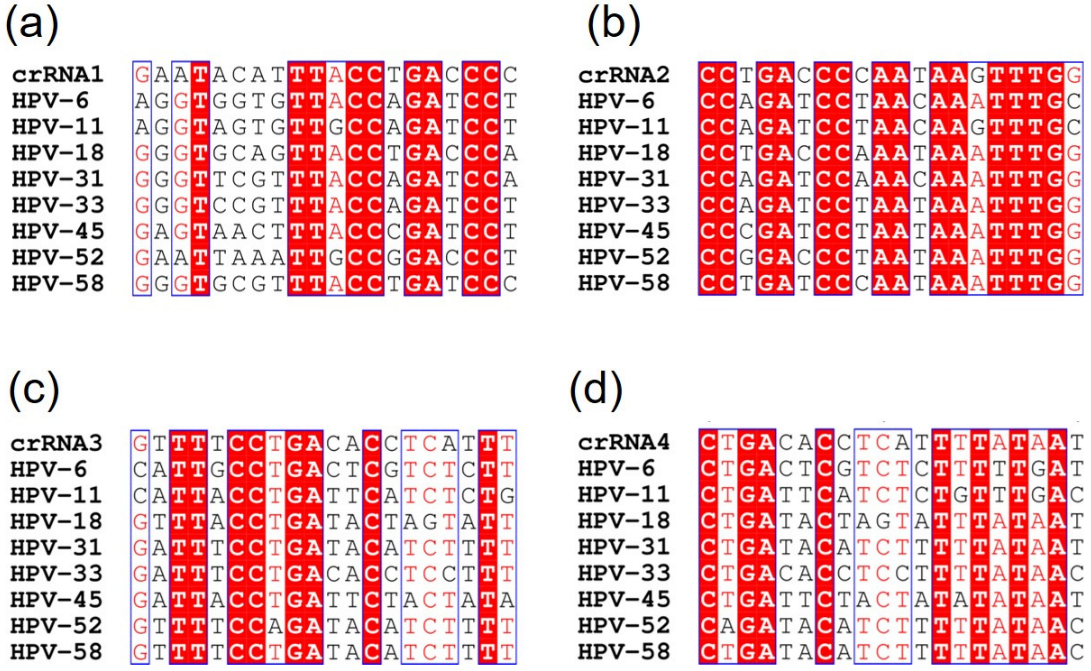

Supplementary Fig. 6 Alignment of the designed HPV-16 crRNAs with the other 8 HPV L1 genes. (a-d) Sequence comparison with crRNA1 (a), crRNA2 (b), crRNA3 (c), crRNA4 (d) and the other L1 genes.

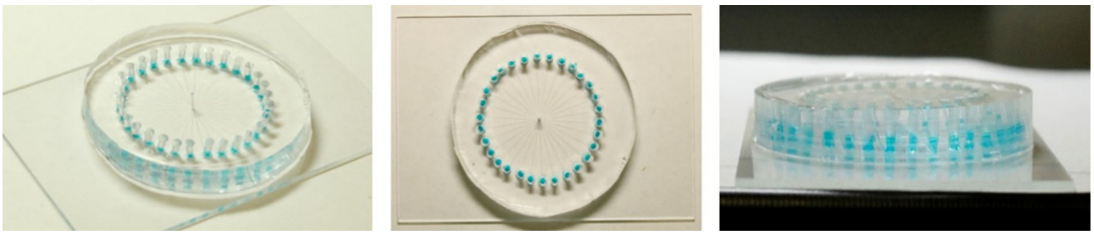

Supplementary Fig. 7 Photographs of the SS-Chip.

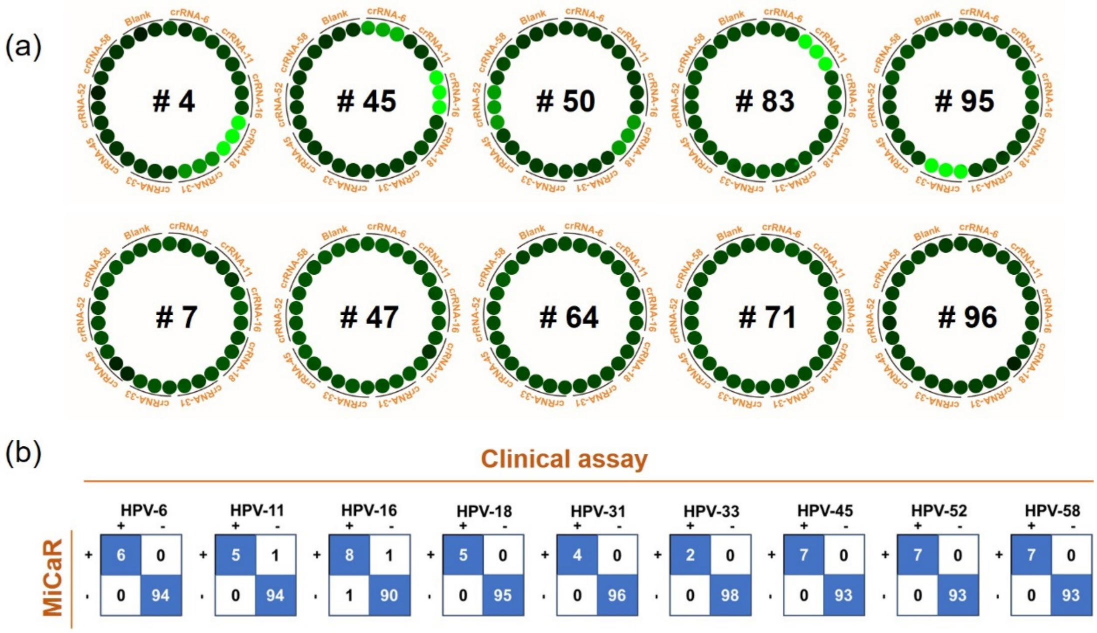

MiCaR	HPV-6 +	HPV-6 -	HPV-11 +	HPV-11 -	HPV-16 +	HPV-16 -	HPV-18 +	HPV-18 -	HPV-31 +	HPV-31 -	HPV-33 +	HPV-33 -	HPV-45 +	HPV-45 -	HPV-52 +	HPV-52 -	HPV-58 +	HPV-58 -
+	6	0	5	1	8	1	5	0	4	0	2	0	7	0	7	0	7	0
-	0	94	0	94	1	90	0	95	0	96	0	98	0	93	0	93	0	93

Supplementary Fig. 8 Testing patient samples on MiCaR. (a) Typical on-chip detection results shown with the original images arranged in a circle. To be noted, #4, #45, #50, #83, and #95 are positive samples, and #7, #47, #64, #71, and #96 are negative samples. These results were in consistent with those obtained in clinic. (b) Detailed testing results for each HPV subtype obtained using MiCaR and the clinical laboratory assay.

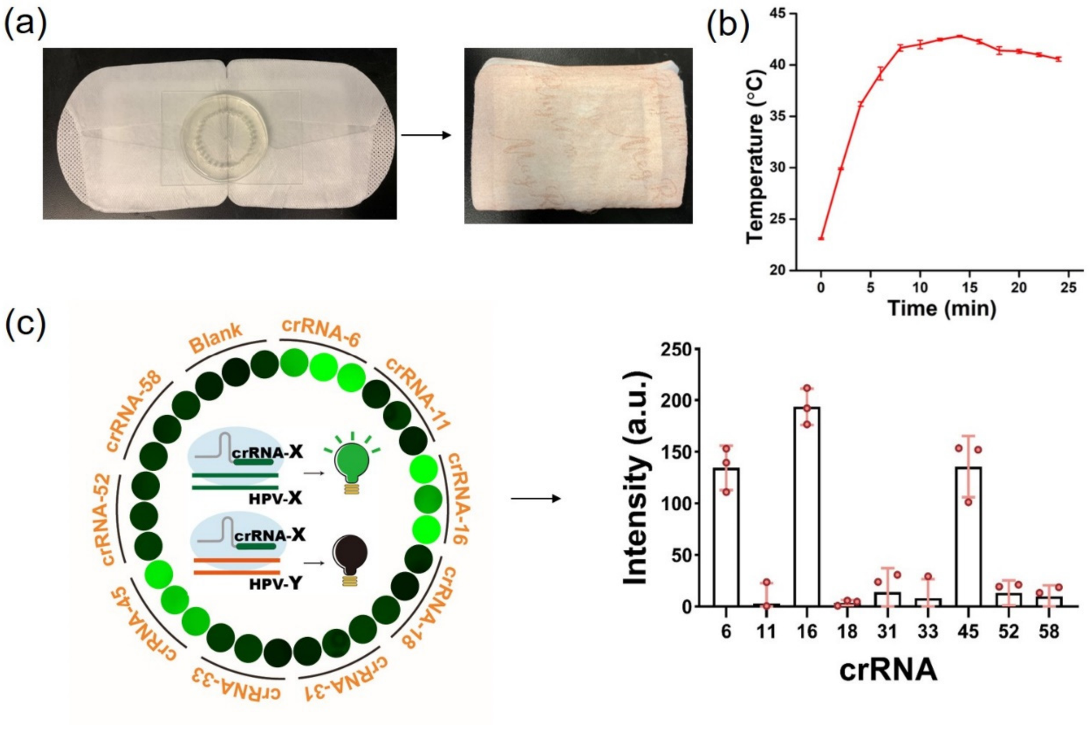

Supplementary Fig. 9 Demonstration of MiCaR as a tool for potential point-of-care testing. (a) Heating the SS-Chip by using a steam eye mask. Heat can be produced when the carbonyl iron powder inside the mask is rapidly oxidized after contacting with oxygen in the air. (b) Temperature on the surface of the eye mask measured with an infrared thermometer. The steam mask can heat the SS-Chip at ~40 ℃ for 20 min. Values represent the mean ± SD and n=3 biologically independent experiments. Source data are provided as a Source Data file. (c) Detection results of Sample #53. The results obtained in this point-of-care way were highly consistent with those obtained on a normal laboratory dry bath. Values represent the mean ± SD and n=3 biologically independent experiments. Source data are provided as a Source Data file.

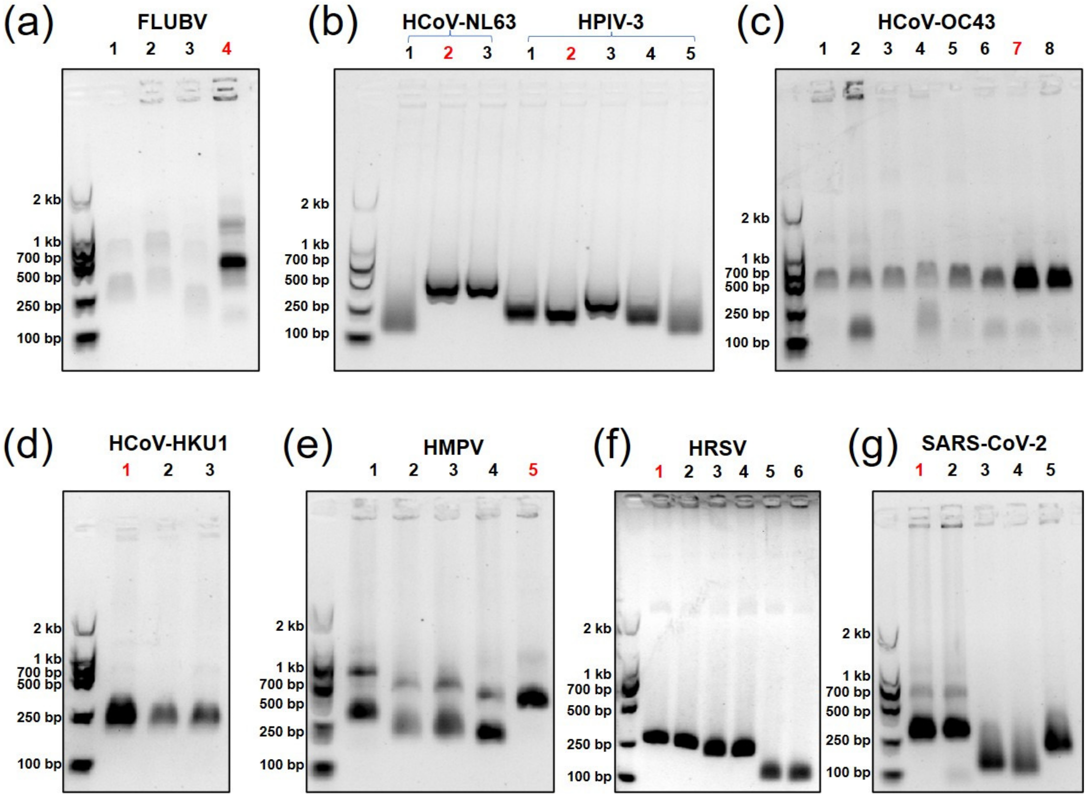

Supplementary Fig. 10 Evaluation of the RPA primers for the 8 respiratory viruses. Multiple pairs of primers were designed and tested for each of the eight viruses. The primer producing a relatively denser band in the gel image was selected as the optimum one, labelled as red fonts on the top of the gel images. Agarose gel imaging results for (a) FLUBV, (b) HPIV-3, (c) HCoV-OC43, (d) HCoV-HKU1, (e) HMPV, (f) HRSV, and (g) SARS-CoV-2. Each experiment was repeated at least two times independently.

Supplementary Table 1. Analysis of the sequence similarity between the L1 gene of any two HPV subtypes.

	HPV-6	HPV-11	HPV-16	HPV-18	HPV-31	HPV-33	HPV-45	HPV-52	HPV-58
**HPV-6**	NA	78.44	67.83	62.12	66.61	64.50	64.13	66.08	66.11
**HPV-11**	78.44	NA	65.96	58.60	69.82	67.39	61.87	64.25	63.52
**HPV-16**	67.83	65.96	NA	62.74	73.77	69.90	66.73	72.78	74.58
**HPV-18**	62.12	58.60	62.74	NA	59.80	58.91	76.98	61.36	61.49
**HPV-31**	66.61	69.82	73.77	59.80	NA	74.52	63.54	68.97	70.16
**HPV-33**	64.50	67.39	69.90	58.91	74.52	NA	62.22	72.63	80.63
**HPV-45**	64.13	61.87	66.73	76.98	63.54	62.22	NA	65.74	66.05
**HPV-52**	66.08	64.25	72.78	61.36	68.97	72.63	65.74	NA	78.58
**HPV-58**	66.11	63.52	74.58	61.49	70.16	80.63	66.05	78.58	NA

Supplementary Table 2. Sequence of HPV primers (Round 1).

Name		Sequence
HPV-6-1	Forward	CCTCCTAACCCTGTATCCAAAGTTGTTGCC
HPV-6-1	Reverse	CCTCCTAACCCTGTATCCAAAGTTGTTGCC
HPV-11a	Forward	CCAAGGTTGTTGCCACGGATGCGTATGTTA
HPV-11a	Reverse	CTGACCAGGATTACCACCATACCCACCAC
HPV-11b	Forward	CCTTTAGGCGTTGGTGTTAGTGGGCATCCATTG
HPV-11b	Reverse	CATCCGATTTATTGGTTTGTAAGTCTGCAA
HPV-16	Forward	CTGTCCCAGTATCTAAGGTTGTAAGCACGG
HPV-16	Reverse	CTAATGGCTGACCACGACCTACCTCAACAC
HPV-18	Forward	CACTGGGCTAAAGGCACTGCTTGTAAATCG
HPV-18	Reverse	CAACTGGGAGTCAGAGGTAACAATAGAGCC
HPV-31	Forward	CTGTCCCAGTGTCTAAAGTTGTAAGCACGG
HPV-31	Reverse	CACTAATACCTACACCTAATGGCTGCCCGC
HPV-33	Forward	CTTGAAATAGGTAGAGGGCAGCCATTAGGC
HPV-33	Reverse	CCTCAATAATAGTATTTATAAGTTCTAAAGGTGG
HPV-45-1	Forward	CCAGTGGCTCTATTATTACTTCTGATTCTC
HPV-45-1	Reverse	CCAAACTTGTAGTAGGTGGTGGAGGGACAC
HPV-52	Forward	CCTGTCTCTAAGGTTGTAAGCACTGATGAG
HPV-52	Reverse	CCCACTAATACCCACACCTAAAGGCTGTCC
HPV-58	Forward	CTCCTGTGCCTGTGTCTAAGGTTGTAAGCA
HPV-58	Reverse	CCAATGGCTGTCCTCTACCTATTTCAAGGC

Supplementary Table 3. Sequence of HPV primers (Round 2).

Name		Sequence
HPV-6a	Forward	CCTAACCCTGTATCCAAAGTTGTTGCCACGG
HPV-6a	Reverse	CTTACACCCACACCTAATGGCTGTCCC
HPV-6b	Forward	CAGCCATTAGGTGTGGGTGTAAGTGGACATCC
HPV-6b	Reverse	CTGGTAATAAGTTCTAAGGGCGGGCAGTCACC
HPV-6c	Forward	CCATTAGGTGTGGGTGTAAGTGGACATCCTTTCC
HPV-6c	Reverse	CTGGTAATAAGTTCTAAGGGCGGGCAGTCACC
HPV-45a	Forward	CCCTTCTCCCAGTGGCTCTATTATTACTTC
HPV-45a	Reverse	CCAAACTTGTAGTAGGTGGTGGAGGGACAC
HPV-45b	Forward	CAGGATACAAAGTGCGAGGTTCCATTAGAC
HPV-45b	Reverse	CAGAAGTAATAATAGAGCCACTGGGAGAAGGG
HPV-45c	Forward	CAACCTGGTGACTGTCCTCCTTTGGAACTT
HPV-45c	Reverse	CAGAAGTAATAATAGAGCCACTGGGAGAAGGG

Supplementary Table 4. Sequence similarity between the primer-amplified region of any two subtypes.

	6a	6b	11a	11b	16	18	31	33	45a	45b	45c	52	58	Average
**6a**	NA	NA	66.13	33.33	67.85	31.82	73.73	33.44	31.27	38.76	31.58	66.36	66.14	49.13
**6b**	NA	NA	29.20	60.67	33.23	23.52	34.27	59.93	29.92	36.23	27.54	31.66	31.68	36.17
**11a**	66.13	29.20	NA	NA	52.11	37.62	56.92	32.32	36.81	29.83	40.90	54.69	50.00	44.23
**11b**	33.33	60.67	NA	NA	35.76	34.52	39.76	49.13	38.34	32.82	36.97	37.13	40.85	39.93
**16**	67.85	33.23	52.11	35.76	NA	29.67	75.24	33.33	35.83	35.00	36.27	70.28	78.55	48.59
**18**	31.82	23.52	37.62	34.52	29.67	NA	31.59	26.25	37.35	52.72	71.39	31.19	30.88	36.54
**31**	73.73	34.27	56.92	39.76	75.24	31.59	NA	34.85	36.17	33.44	37.11	72.14	72.64	49.82
**33**	33.44	59.93	32.32	49.13	33.33	26.25	34.85	NA	34.14	35.02	32.27	35.45	31.68	36.48
**45a**	31.27	29.92	36.81	38.34	35.83	37.35	36.17	34.14	NA	NA	NA	36.07	34.58	35.05
**45b**	38.76	36.23	29.83	32.82	35.00	52.72	33.44	35.02	NA	NA	NA	34.56	36.48	36.49
**45c**	31.58	27.54	40.90	36.97	36.27	71.39	37.11	32.27	NA	NA	NA	35.77	33.42	38.32
**52**	66.36	31.66	54.69	37.13	70.28	31.19	72.14	35.45	36.07	34.56	35.77	NA	72.09	48.12
**58**	66.14	31.68	50.00	40.85	78.55	30.88	72.64	31.68	34.58	36.48	33.42	72.09	NA	46.62

Supplementary Table 5. HPV primer sequences used in the final panel.

Name		Sequence
HPV-6b	Forward	CAGCCATTAGGTGTGGGTGTAAGTGGACATCC
HPV-6b	Reverse	CTGGTAATAAGTTCTAAGGGCGGGCAGTCACC
HPV-11b	Forward	CCTTTAGGCGTTGGTGTTAGTGGGCATCCATTG
HPV-11b	Reverse	CATCCGATTTATTGGTTTGTAAGTCTGCAA
HPV-16	Forward	CTGTCCCAGTATCTAAGGTTGTAAGCACGG
HPV-16	Reverse	CTAATGGCTGACCACGACCTACCTCAACAC
HPV-18	Forward	CACTGGGCTAAAGGCACTGCTTGTAAATCG
HPV-18	Reverse	CAACTGGGAGTCAGAGGTAACAATAGAGCC
HPV-31	Forward	CTGTCCCAGTGTCTAAAGTTGTAAGCACGG
HPV-31	Reverse	CACTAATACCTACACCTAATGGCTGCCCGC
HPV-33	Forward	CTTGAAATAGGTAGAGGGCAGCCATTAGGC
HPV-33	Reverse	CCTCAATAATAGTATTTATAAGTTCTAAAGGTGG
HPV-45a	Forward	CCCTTCTCCCAGTGGCTCTATTATTACTTC
HPV-45a	Reverse	CCAAACTTGTAGTAGGTGGTGGAGGGACAC
HPV-52	Forward	CCTGTCTCTAAGGTTGTAAGCACTGATGAG
HPV-52	Reverse	CCCACTAATACCCACACCTAAAGGCTGTCC
HPV-58	Forward	CTCCTGTGCCTGTGTCTAAGGTTGTAAGCA
HPV-58	Reverse	CCAATGGCTGTCCTCTACCTATTTCAAGGC

Supplementary Table 6. Sequence of crRNAs tested initially.

Name		Sequence
HPV-6	crRNA-I	UAAUUUCUACUAAGUGUAGAUUCAUGCCAGCAGUUCUAGAC
HPV-11	crRNA	UAAUUUCUACUAAGUGUAGAUAACAUUGUGUACCCUUACCC
HPV-16	crRNA	UAAUUUCUACUAAGUGUAGAUGAAUACAUUUACCUGACCCC
HPV-18	crRNA	UAAUUUCUACUAAGUGUAGAUGAAGAUGGUGAUAUGGUAGA
HPV-31	crRNA	UAAUUUCUACUAAGUGUAGAUGGCCUGUGUUGGUUUAGAGG
HPV-33	crRNA	UAAUUUCUACUAAGUGUAGAUCUUGGAUGUAAGCCUCCAAC
HPV-45	crRNA	UAAUUUCUACUAAGUGUAGAUUUGGCAUAAUCAGUUGUUUG
HPV-52	crRNA	UAAUUUCUACUAAGUGUAGAUGUUCCCAAGGUGUCUGGCCU
HPV-58	crRNA	UAAUUUCUACUAAGUGUAGAUCAUCAAGAGUCCCAAUAACA

Supplementary Table 7. Sequence of crRNAs finally used.

Name		Sequence
HPV-6	crRNA	UAAUUUCUACUAAGUGUAGAUCCUUUACCCCAAUGCUCGCCC
HPV-11	crRNA	UAAUUUCUACUAAGUGUAGAUAACAUUGUGUACCCUUACCC
HPV-16	crRNA	UAAUUUCUACUAAGUGUAGAUGAAUACAUUUACCUGACCCC
HPV-18	crRNA	UAAUUUCUACUAAGUGUAGAUGAAGAUGGUGAUAUGGUAGA
HPV-31	crRNA	UAAUUUCUACUAAGUGUAGAUGGCCUGUGUUGGUUUAGAGG
HPV-33	crRNA	UAAUUUCUACUAAGUGUAGAUCUUGGAUGUAAGCCUCCAAC
HPV-45	crRNA	UAAUUUCUACUAAGUGUAGAUUUGGCAUAAUCAGUUGUUUG
HPV-52	crRNA	UAAUUUCUACUAAGUGUAGAUGUUCCCAAGGUGUCUGGCCU
HPV-58	crRNA	UAAUUUCUACUAAGUGUAGAUCAUCAAGAGUCCCAAUAACA

Supplementary Table 8. Information of the clinic samples tested in this work. All the samples were di-identified during the collection. All the participants were female and the ages range from 17 to 77. Please note, the samples were collected in multiple times on different dates. And the sample labeling is based on the on-chip testing order, not the original order.

Sample	Date	HPV-6	11	16	18	31	33	45	52	58
1	2020/9/23	-	-	-	-	-	-	+	-	-
2	2020/9/24	-	-	-	-	-	-	-	-	-
3	2020/10/28	-	-	-	-	-	-	-	-	-
4	2020/10/29	-	-	-	+	+	-	-	-	-
5	2020/10/29	-	-	-	-	-	-	-	-	-
6	2020/11/9	-	-	-	+	-	-	-	-	-
7	2020/11/10	-	-	-	-	-	-	-	-	-
8	2020/11/11	-	-	-	-	-	-	-	-	-
9	2020/11/15	-	+	-	-	-	-	-	-	-
10	2020/11/23	-	-	-	-	-	-	-	-	-
11	2020/12/6	-	-	-	-	+	-	-	-	-
12	2020/12/13	-	-	-	-	-	-	-	-	-
13	2020/12/14	-	-	-	-	-	-	-	-	-
14	2020/12/14	-	-	-	-	-	-	-	-	-
15	2020/12/22	-	-	-	-	-	-	+	-	-
16	2020/12/27	-	-	-	-	-	-	-	-	-
17	2020/12/27	-	-	-	-	-	-	-	-	-
18	2020/12/29	-	-	-	-	-	-	-	-	-
19	2020/12/29	-	-	-	-	-	-	+	-	-
20	2021/1/6	-	-	-	-	-	-	-	-	-
21	2021/1/8	+	-	-	-	-	-	-	-	-
22	2021/1/10	-	-	-	-	-	-	-	-	-
23	2021/1/10	-	-	-	-	-	-	+	-	-
24	2021/1/13	-	-	-	-	-	-	-	-	-
25	2021/1/13	+	-	-	-	-	-	-	-	-
26	2021/1/13	-	-	-	-	-	-	-	-	-
27	2021/1/14	-	-	-	-	+	-	-	-	-
28	2021/5/9	-	-	-	-	-	-	-	-	-
29	2021/5/19	-	-	-	-	-	-	-	-	-
30	2021/5/9	-	+	-	-	-	-	-	+	-
31	2021/5/19	-	-	-	-	-	-	-	-	-
32	2021/5/20	-	-	-	-	-	-	+	-	-
33	2021/5/20	-	-	-	-	-	-	-	-	-
34	2021/5/23	-	-	-	-	-	-	-	-	-
35	2021/5/27	+	-	-	-	-	-	-	-	-
36	2021/5/31	-	-	-	-	+	-	-	-	-
37	2021/5/23	-	-	-	-	-	-	-	-	-
38	2020/12/27	-	-	+	-	-	-	-	+	-
39	2020/12/27	-	-	-	-	-	-	+	-	-
40	2021/5/23	-	-	-	-	-	-	-	-	-
41	2020/12/29	-	+	-	-	-	-	-	-	-
42	2020/12/29	-	+	-	-	-	-	-	-	-
43	2021/5/23	-	-	-	-	-	-	-	-	-
44	2021/1/6	-	-	-	-	-	-	-	-	+
45	2021/1/8	+	-	+	-	-	-	-	-	-
46	2021/5/23	-	-	-	-	-	-	-	-	-
47	2021/5/23	-	-	-	-	-	-	-	-	-
48	2021/5/23	-	-	-	-	-	-	-	-	-
49	2021/1/10	-	-	-	-	-	+	-	-	-
50	2021/1/10	-	-	-	+	-	-	-	+	-
51	2021/1/13	-	-	-	-	-	-	-	-	+
52	2021/1/13	-	-	+	-	-	-	-	-	-
53	2021/1/13	+	-	+	-	-	-	+	-	-
54	2021/5/23	-	-	-	-	-	-	-	-	-
55	2021/5/23	-	-	-	-	-	-	-	-	-
56	2021/1/14	-	-	+	-	-	-	-	-	-
57	2021/5/23	-	-	-	-	-	-	-	-	-
58	2021/5/23	-	-	-	-	-	-	-	-	-
59	2021/5/23	-	-	-	-	-	-	-	-	-
60	2021/5/9	-	-	+	-	-	-	-	-	-
61	2021/5/19	-	-	-	-	-	-	-	+	-
62	2021/5/23	-	-	-	-	-	-	-	-	-
63	2021/5/23	-	-	-	-	-	-	-	-	-
64	2021/5/23	-	-	-	-	-	-	-	-	-
65	2021/5/23	-	-	-	-	-	-	-	-	-
66	2021/5/9	-	-	+	-	-	-	-	-	-
67	2021/5/23	-	-	-	-	-	-	-	-	-
68	2021/5/19	-	-	+	-	-	-	-	-	-
69	2021/5/23	-	-	-	-	-	-	-	-	-
70	2021/5/20	-	-	-	-	-	+	-	-	+
71	2021/5/21	-	-	-	-	-	-	-	-	-
72	2021/5/21	-	-	-	-	-	-	-	-	-
73	2021/5/20	-	-	-	-	-	-	-	+	-
74	2021/5/23	-	-	-	-	-	-	-	+	-
75	2021/5/27	-	-	-	-	-	-	-	-	+
76	2021/5/31	-	-	-	-	-	-	-	-	+
77	2021/5/21	-	-	-	-	-	-	-	-	-
78	2021/5/21	-	-	-	-	-	-	-	-	-
79	2021/5/21	-	-	-	-	-	-	-	-	-
80	2021/5/21	-	-	-	-	-	-	-	-	-
81	2021/5/21	-	-	-	-	-	-	-	-	-
82	2021/5/21	-	-	-	-	-	-	-	-	-
83	2021/1/3	-	-	-	+	-	-	-	-	-
84	2021/1/5	-	-	+	-	-	-	-	-	-
85	2021/5/21	-	-	-	-	-	-	-	-	-
86	2021/1/10	-	-	-	-	-	-	-	-	+
87	2021/5/21	-	-	-	-	-	-	-	-	-
88	2021/5/21	-	-	-	-	-	-	-	-	-
89	2021/5/9	-	-	-	+	-	-	-	-	-
90	2021/5/21	-	-	-	-	-	-	-	-	-
91	2021/5/21	-	-	-	-	-	-	-	-	+
92	2021/5/21	-	-	-	-	-	-	-	-	-
93	2021/5/21	-	-	-	-	-	-	-	-	-
94	2021/5/26	-	-	-	-	-	-	-	+	-
95	2021/5/30	-	-	-	-	-	+	-	-	-
96	2021/5/21	-	-	-	-	-	-	-	-	-
97	2021/5/21	-	-	-	-	-	-	-	-	-
98	2021/5/30	+	-	-	-	-	-	-	-	-
99	2021/5/31	-	+	-	-	-	-	-	-	-
100	2021/5/21	-	-	-	-	-	-	-	-	-

Supplementary Table 9. RPA primers designed for the 8 respiratory viruses. The optimum primers finally used were shown as bold fonts.

Name			Sequence
FLUBV	1	Forward	CAACGGCACTAAACACAACAATAACCTCCTTC
		Reverse	CCTGTCTTTTACTTTCATTGGTATTCTCTTTATG
	2	Forward	CATAAAGAGAATACCAATGAAAGTAAAAGACAGG
		Reverse	CCTTCTTTTCATTTCCACCCACGGGCAAACCAC
	3	Forward	CATAAAGAGAATACCAATGAAAGTAAAAGACAGG
		Reverse	CTGTCTCTGGTTATTCTTTCAGTCATAGCC
	**4**	**Forward**	**CTATGACTGAAAGAATAACCAGAGACAGCCC**
		**Reverse**	**CCATCCCATAAGTATTCCTTGTTTCCAATG**
HCoV-NL63	1	Forward	CTTGGTGATAGTGGTAAACTTCTTAGTGAAC
		Reverse	CATAATCCAAAACAAACAACCAACTTGCTC
	**2**	**Forward**	**CAAGTTGGTTGTTTGTTTTGGATTATGCCC**
		**Reverse**	**CACTATGAAAATCAACATCAACAAAACAGAC**
	3	Forward	CAAGTTGGTTGTTTGTTTTGGATTATGCCC
		Reverse	CACTATGAAAATCAACATCAACAAAACAGACAG
HCoV-OC43	1	Forward	ATGTTTGAGGACGCAGAGGAGAAGTTGGATAACCC
		Reverse	TTACACACACTTCTACGCCGAAACAAACCC
	2	Forward	ATGTTTGAGGACGCAGAGGAGAAGTTGGATAACCC
		Reverse	GTTACACACACTTCTACGCCGAAACAAACCC
	3	Forward	TGTTTGAGGACGCAGAGGAGAAGTTGGATAACCC
		Reverse	TTACACACACTTCTACGCCGAAACAAACCC
	4	Forward	TGTTTGAGGACGCAGAGGAGAAGTTGGATAACCC
		Reverse	GTTACACACACTTCTACGCCGAAACAAACCC
	5	Forward	GTTTGAGGACGCAGAGGAGAAGTTGGATAACCC
		Reverse	TTACACACACTTCTACGCCGAAACAAACCC
	6	Forward	GTTTGAGGACGCAGAGGAGAAGTTGGATAACCC
		Reverse	GTTACACACACTTCTACGCCGAAACAAACCC
	**7**	**Forward**	**TTTGAGGACGCAGAGGAGAAGTTGGATAACCC**
		**Reverse**	**TTACACACACTTCTACGCCGAAACAAACCC**
	8	Forward	TTTGAGGACGCAGAGGAGAAGTTGGATAACCC
		Reverse	GTTACACACACTTCTACGCCGAAACAAACCC
HRSV	**1**	**Forward**	**CTAGTGAAACAAATATCCACACCCAAAGGACCC**
		**Reverse**	**CATGTGTTGGGTTGAGTGTTTTCATAGTGAG**
	2	Forward	CAAATATCCACACCCAAAGGACCCTCATTAAGAG
		Reverse	CATGTGTTGGGTTGAGTGTTTTCATAGTGAG
	3	Forward	CTCACTATGAAAACACTCAACCCAACACATGAC
		Reverse	GTGATGACTAACAGTAATCCTGAGTAAGGG
	4	Forward	CTCACTATGAAAACACTCAACCCAACACATGAC
		Reverse	TGTGATGACTAACAGTAATCCTGAGTAAGGG
	5	Forward	CTGACAACAAAGGAGCATTCAAATACATAAAGCC
		Reverse	CGTGTAGCTGTGTGCTTCCAATTTGTTGTAAC
	6	Forward	CAACAAAGGAGCATTCAAATACATAAAGCC
		Reverse	CGTGTAGCTGTGTGCTTCCAATTTGTTGTAAC

Name			Sequence
HCoV-HKU1	**1**	**Forward**	**CCCAATCATCTGGTGTTATTCCTGAAAATCC**
		**Reverse**	**CCTGAATAAACCAAATCATCATAAGACTTGACTG**
	2	Forward	CCCAATCATCTGGTGTTATTCCTGAAAATCCTGTG
		Reverse	CTGAATAAACCAAATCATCATAAGACTTGACTG
	3	Forward	CCAATCATCTGGTGTTATTCCTGAAAATCCTGTG
		Reverse	CTACACCTGAATAAACCAAATCATCATAAGACTTG
SARS-CoV-2	**1**	**Forward**	**CTGTCTATCCAGTTGCGTCACCAAATGAATGC**
		**Reverse**	**CATTATGGTATTCGGCAAGACTATGCTCAGGTCC**
	2	Forward	CTGTCTATCCAGTTGCGTCACCAAATGAATGC
		Reverse	CAGATTCATTATGGTATTCGGCAAGACTATGC
	3	Forward	CAGAAGTAGGACCTGAGCATAGTCTTGCCG
		Reverse	CAACATAAGAGAACACACAGCCTCCAAAGG
	4	Forward	CCTGAGCATAGTCTTGCCGAATACCATAATG
		Reverse	CAACATAAGAGAACACACAGCCTCCAAAGG
	5	Forward	CATAGTCTTGCCGAATACCATAATGAATCTGGC
		Reverse	CCTTCGGAACCTTCTCCAACAACACCTGTATGG
HPIV-3	1	Forward	CGAGATGGAACGAATCAAAGATAAATACGGG
		Reverse	CCATCTCTTTTGCTTTGACTGTTCTTCTCACTTC
	**2**	**Forward**	**CGAGATGGAACGAATCAAAGATAAATACGGG**
		**Reverse**	**CAACCATCTCTTTTGCTTTGACTGTTCTTCTCAC**
	3	Forward	CGAGATGGAACGAATCAAAGATAAATACGGG
		Reverse	CGAACAGCATTCCTTTTCTTAGTCTACTGG
	4	Forward	CTGGGCTTCATCAGTAGAGATTACAAGAGTGG
		Reverse	TGATTATCCTTTATTTGCTCTGTCTTAGTGGG
	5	Forward	CCAACCTTCTTTACCTGGCGAGTTCAGATAC
		Reverse	TGATTATCCTTTATTTGCTCTGTCTTAGTGGG
HMPV	1	Forward	CAGTTTCTGCTGACCAGTTGGCAAGAGAGG
		Reverse	CTTGCTCACAAAATCTTTCAGTTCTCTCAC
	2	Forward	CTACTACCCAAATGAGAAGGACTGTGAAACAAGG
		Reverse	ACAAGCAACCAGAGCCCCAAGAGGAGATAG
	3	Forward	CTACCCAAATGAGAAGGACTGTGAAACAAGGGG
		Reverse	ACAAGCAACCAGAGCCCCAAGAGGAGATAG
	4	Forward	CCCAAATGAGAAGGACTGTGAAACAAGGGG
		Reverse	ACAAGCAACCAGAGCCCCAAGAGGAGATAG
	**5**	**Forward**	**CTATCTCCTCTTGGGGCTCTGGTTGCTTGTTAC**
		**Reverse**	**CAATGATGAAGCCAGTGTTCCCTTTCTCTGC**

Supplementary Table 10. crRNAs designed for recognizing the target sequence of the 8 respiratory viruses.

Name	Sequence
FLUBV crRNA	UAAUUUCUACUAAGUGUAGAUCAACAAAAUAGCCAGAUUAG
HCoV-NL63 crRNA	UAAUUUCUACUAAGUGUAGAUAAAAAGGUGAGUGUUGUAUU
HCoV-OC43 crRNA	UAAUUUCUACUAAGUGUAGAUCUCCACCACUGCGCAAAAGC
HRSV crRNA	UAAUUUCUACUAAGUGUAGAUACAGGGUGUGGUUACAUCAU
HCoV-HKU1 crRNA	UAAUUUCUACUAAGUGUAGAUUAUGGUUAUUCUGUCACACC
SARS-CoV-2 crRNA	UAAUUUCUACUAAGUGUAGAUACUAAAGAAGGUGCCACUAC
HPIV-3 crRNA	UAAUUUCUACUAAGUGUAGAUUAACUCGGGUCACUAUCAAG
HMPV crRNA	UAAUUUCUACUAAGUGUAGAUAGAACAUUGAAAACAGCCAG
