# Supplementary material for: Microfluidic space coding for multiplexed nucleic acid detection via CRISPR-Cas12a and recombinase polymerase amplification
Source: Nat Commun. 2022 Oct 29;13:6480. doi: 10.1038/s41467-022-34086-y (PMC9617605; doi:10.1038/s41467-022-34086-y)
Supplement: Supplementary file 3 — Description of additional supplementary files [file 41467_2022_34086_MOESM3_ESM.pdf]

Description of additional supplementary files

Supplementary Data 1. Sequence alignment of 9 types of HPV L1 genes.

Supplementary Data 2. Sequence alignment of amplification region.

Supplementary Data 3. Sequences of amplification region and HPV L1 genes.

Supplementary Data 4. Sequence alignment of Top 5 amplicons of the 9 HPV subtypes.

Supplementary Data 5. Sequences of RVP primers, crRNAs and genes.

Supplementary Movie 1. Sample injection.
