# Supplementary material for: Microfluidic space coding for multiplexed nucleic acid detection via CRISPR-Cas12a and recombinase polymerase amplification
Source: Nat Commun. 2022 Oct 29;13:6480. doi: 10.1038/s41467-022-34086-y (PMC9617605; doi:10.1038/s41467-022-34086-y)
Supplement: Supplementary file 4 — Supplementary Data 2 [file 41467_2022_34086_MOESM4_ESM.pdf]

Supplementary Data 2

Sequence alignment of amplification region

```
        1        10        20        30        40        50        60
HPV-6   CTGCTTTACCTACAGGCCCTGTTTTCATTACAGGTTCTGGATTTTATTTGCATCCTGCAT
HPV-11  ............................................................

                 70        80        90       100       110       120
HPV-6   GGTATTTTGCACGTAAACGCCGTAAACGTATTCCCTTATTTTTTTCAGATGTGGCGGCCT
HPV-11  ..............................................GAATGTGGCGGCCT

               130       140       150       160       170       180
HPV-6   AGCGACAGCACAGTATATGTGCCTCCTCCTAACCCTGTATCCAAAGTTGTTGCCACGGAT
HPV-11  AGCGACAGCACAGTATATGTGCCTCCTCCCAACCCTGTATCCAAGGTTGTTGCCACGGAT

               190       200       210       220       230       240
HPV-6   GCTTATGTTACTCGCACCAACATATTTTATCATGCCAGCAGTTCTAGACTTCTTGCAGTG
HPV-11  GCGTATGTTAAACGCACCAACATATTTTATCATGCCAGCAGTTCTAGACTCCTTGCTGTG

               250       260       270       280       290       300
HPV-6   GGTCATCCTTATTTTTCCATAAAACGGGCTAACAAAACTGTTGTGCCAAAGGTGTCAGGA
HPV-11  GGACATCCATATTACTCTATCAAAAAAGTTAACAAAACAGTTGTACCAAAGGTGTCTGGA

               310       320       330       340       350       360
HPV-6   TATCAATACAGGGTATTTAAGGTGGTGTTACCAGATCCTAACAAATTTGCATTGCCTGAC
HPV-11  TATCAATATAGAGTGTTTAAGGTAGTGTTGCCAGATCCTAACAAGTTTGCATTACCTGAT

               370       380       390       400       410       420
HPV-6   TCGTCTCTTTTTGATCCCACAACACAACGTTTGGTATGGGCATGCACAGGCCTAGAGGTG
HPV-11  TCATCTCTGTTTGACCCCACTACACAGCGTTTAGTATGGGCGTGCACAGGGTTGGAGGTA

               430       440       450       460       470       480
HPV-6   GGCAGGGGACAGCCATTAGGTGTGGGTGTAAGTGGACATCCTTTCCTAAATAAATATGAT
HPV-11  GGCAGGGGTCAACCTTTAGGCGTTGGTGTTAGTGGGCATCCATTGCTAAACAAATATGAT

               490       500          510       520       530
HPV-6   GATGTTGAAAATTCAGGGAG...TGGTGGTAACCCTGGACAGGATAACAGGGTTAATGTT
HPV-11  GATGTAGAAAATAGTGGTGGGTATGGTGGTAATCCTGGTCAGGATAATAGGGTTAATGTA

        540       550       560       570       580       590
HPV-6   GGTATGGATTATAAACAAACACAATTATGCATGGTTGGATGTGCCCCCCCTTTGGGCGAG
HPV-11  GGTATGGATTATAAACAAACCCAGCTATGTATGGTGGGCTGTGCTCCACCGTTAGGTGAA

        600       610       620       630       640       650
HPV-6   CATTGGGGTAAAGGTAAACAGTGTACTAATACACCTGTACAGGCTGGTGACTGCCCGCCC
HPV-11  CATTGGGGTAAGGGTACACAATGTTCAAATACCTCTGTACAAAATGGTGACTGCCCCCCG

        660       670       680       690       700       710
HPV-6   TTAGAACTTATTACCAGTGTTATACAGGATGGCGATATGGTTGACACAGGCTTTGGTGCT
HPV-11  TTGGAACTTATTACCAGTGTTATACAGGATGGGGACATGGTTGATACAGGCTTTGGTGCT
```

```
       720       730       740       750       760       770
HPV-6  ATGAATTTTGCTGATTTGCAGACCAATAAATCAGATGTTCCTATTGACATATGTGGCACT
HPV-11 ATGAATTTTGCAGACTTACAAACCAATAAATCGGATGTTCCCCTTGATATTTGTGGAACT

       780       790       800       810       820       830
HPV-6  ACATGTAAATATCCAGATTATTTACAAATGGCTGCAGACCCATATGGTGATAGATTATTT
HPV-11 GTCTGCAAATATCCTGATTATTTGCAAATGGCTGCAGACCCTTATGGTGATAGGTTGTTT

       840       850       860       870       880       890
HPV-6  TTTTTTCTACGGAAGGAACAAATGTTTGCCAGACATTTTTTTAACAGGGCTGGCGAGGTG
HPV-11 TTTTATTTGCGAAAGGAACAAATGTTTGCTAGACACTTTTTTAATAGGGCCGGTACTGTG

       900       910       920       930       940       950
HPV-6  GGGGAACCTGTGCCTGATACTCTTATAATTAAGGGTAGTGGAAATCGAACGTCTGTAGGG
HPV-11 GGGGAACCTGTGCCTGATGACCTGTTGGTAAAAGGGGGTAATAACAGATCATCTGTAGCT

       960       970       980       990      1000      1010
HPV-6  AGTAGTATATATGTTAACACCCCAAGCGGCTCTTTGGTGTCCTCTGAGGCACAATTGTTT
HPV-11 AGTAGTATTTATGTACATACACCTAGTGGCTCATTGGTGTCTTCAGAGGCTCAATTATTT

      1020      1030      1040      1050      1060      1070
HPV-6  AATAAGCCATATTGGCTACAAAAAGCCCAGGGACATAACAATGGTATTTGTTGGGGTAAT
HPV-11 AATAAACCATATTGGCTTCAAAAGGCTCAGGGACATAACAATGGTATTTGCTGGGGAAAC

      1080      1090      1100      1110      1120      1130
HPV-6  CAACTGTTTGTTACTGTGGTAGATACCACACGCAGTACCAACATGACATTATGTGCATCC
HPV-11 CACTTGTTTGTTACTGTGGTAGATACCACACGCAGTACAAATATGACACTATGTGCATCT

      1140      1150      1160      1170      1180      1190
HPV-6  GTAACTACATCGTCCACATACACCAATTCTGATTATAAAGAGTACATGCGTCATGTGGAA
HPV-11 GTGTCTAAATCTGCTACATACACTAATTCAGATTATAAGGAATACATGCGCCATGTGGAG

      1200      1210      1220      1230      1240      1250
HPV-6  GAGTATGATTTACAATTTATTTTTCAATTATGTAGCATTACATTGTCTGCTGAAGTAATG
HPV-11 GAGTTTGATTTACAGTTTATTTTTCAATTGTGTAGCATTACATTATCTGCAGAAGTCATG

      1260      1270      1280      1290      1300      1310
HPV-6  GCCTATATTCACACAATGAATCCCTCTGTTTTGGAAGACTGGAACTTTGGGTTATCGCCT
HPV-11 GCCTATATACACACAATGAATCCTTCTGTTTTGGAGGACTGGAACTTTGGTTTATCGCCT

      1320      1330      1340      1350      1360      1370
HPV-6  CCCCCAAATGGTACATTAGAAGATACCTATAGGTATGTGCAGTCACAGGCCATTACCTGT
HPV-11 CCACCAAATGGTACACTGGAGGATACTTATAGATATGTACAGTCACAGGCCATTACCTGT

      1380      1390      1400      1410      1420      1430
HPV-6  CAAAAGCCCACTCCTGAACAGCAAAAGCCAGATCCCTATAAGAACCTTAGTTTTTGGGAG
HPV-11 CAGAAACCCACACCTGAAAAAGAAAAACAGGATCCCTATAAGGATATGAGTTTTTGGGAG
```

```
         1440      1450      1460      1470      1480      1490
           .         .         .         .         .         .
HPV-6    GTTAATTTAAAAGAAAAGTTTTCTAGTGAATTGGATCAGTATCCTTTGGGACGCAAGTTT
HPV-11   GTTAACTTAAAAGAAAAGTTTTCAAGTGAATTAGATCAGTTTCCCCTTGGACGTAAGTTT

         1500      1510      1520      1530      1540      1550
           .         .         .         .         .         .
HPV-6    TTGTTACAAAGTGGATATAGGGGACGGTCCTCTATTCGTACCGGTGTTAAGCGCCCTGCT
HPV-11   TTATTGCAAAGTGGATATCGAGGACGGACGTCTGCTCGTACAGGTATAAAGCGCCCAGCT

         1560      1570      1580      1590      1600      1610
           .         .         .         .         .         .
HPV-6    GTTTCCAAAGCCTCTGCTGCCCCTAAACGTAAGCGCGCCAAAACCAAAAGGTAA
HPV-11   GTGTCTAAGCCCTCTACAGCCCCCAAACGAAAACGTACCAAAACCAAAAAGTAA
```

```
     1        10        20        30        40        50        60        70        80        90
6a   CCTAACCCTGTATCCAAAGTTGTTGCCACGGATGCTTATGTTACTCGCACCAACATATTTTATCATGCCAGCAGTTCTAGACTTCTTGCAGTGGGTCA
11a  .............CCAAGGTTGTTGCCACGGATGCGTATGTTAACGCACCAACATATTTTATCATGCCAGCAGTTCTAGACTCCTTGCTGTGGGACA

    100       110       120       130       140       150       160       170       180       190
6a   TCCTTATTTTTCCATAAAACGGGCTAACAAAACTGTTGTGCCAAAGGTGTCAGGATATCAATACAGGGTATTTAAGGTGGTGTTACCAGATCCTAACA
11a  TCCATATTACTCTATCAAAAAAGTTAACAAAACAGTTGTACCAAAGGTGTCTGGATATCAATATAGAGTGTTTAAGGTAGTGTTGCCAGATCCTAACA

       200       210       220       230       240       250       260       270       280       290
6a   AATTTGCATTGCCTGACTCGTCTCTTTTTGATCCCACAACACAACGTTTGGTATGGGCATGCACAGGCCTAGAGGTGGGCAGGGGACAGCCATTAGGT
11a  AGTTTGCATTACCTGATTCATCTCTGTTTGACCCCACTACACAGCGTTTAGTATGGGCGTGCACAGGGTTGGAGGTAGGCAGGGGTCAACCTTTAGGC

         300
6a   GTGGGTGTAAG......................................................................
11a  GTTGGTGTTAGTGGGCATCCATTGCTAAACAAATATGATGATGTAGAAAATAGTGGTGGGTATGGTGGTAATCCTGGTCAG
```

```
                                 1        10        20        30        40        50        60        70
6a   ........................CCTAACCCTGTATCCAAAGTTGTTGCCACGGATGCTTATGTTACTCGCACCAACATATTTTATCATGCCAGCAG
11b  CCTTTAGGCGTTGGTGTTAGTGGGCATCCATTGCTAAACAAATATGATGATGTAGAAAATAGTGGTGGGTATGGTGGTAATCCTGGTCAGGATAATAG

         80        90       100       110       120       130       140       150       160       170
6a   TTCTAGACTTCTTGCAGTGGGTCATCCTTATTTTTCCATAAAACGGGCTAACAAAACTGTTGTGCCAAAGG..TGTCAGGATATCAATACAGGGTATT
11b  GGTTAA...TGTAGGTATGGATTATAAACAAACCCAGCTATGTATGGTGGGCTGTGCTCCACCGTTAGGTGAACATTGGGGTAAGGGTACACAATGTT

            180       190       200       210       220       230       240       250       260
6a   TAAGGTGGTGT.TACCAGATCCTAACAAATTTGCATTGCCTGAC.TCGTCTCTTTTTGATCCCACAACACAACGTTTGGTATGGCATGCACAGGCCT
11b  CAAATACCTCTGTACAAAATGGTGACTGCCCCCCGTTGGAACTTATTACCAGTGTTATACAGGATGGGGACATGGTTGATACAGGCTTTGGTGCTATG

       270       280       290       300
6a   AGAGGTGGGCAGGGGACAGCCATTAGGTGTGGGTGTAAG
11b  AATTTTGCAGACTTACAAACCAATAAAT.CGGATG....
```

```
       1        10        20        30        40        50        60        70        80        90
6a     CCTAACCCTGTATCCAAAGTTGTTGCCACGGATGCTTATGTTACTCGCACCAACATATTTTATCATGCCAGCAGTTCTAGACTTCTTGCAGTGGGTCA
16     .CTGTCCCAGTATCTAAGGTTGTAAGCACGGATGAATATGTTGCACGCACAAACATATATTATCATGCAGGAACATCCAGACTACTTGCAGTTGGACA

     100       110       120            130       140       150       160       170       180       190
6a     TCCTTATTTTTCCATAAAACGGGCTAACAA.....AACTGTT.GTGCCAAAGGTGTCAGGATATCAATACAGGGTATTTAAGGTGGTGTTACCAGATC
16     TCCCTATTTTCCTATTAAAAAACCTAACAATAACAAAATATTAGTTCCTAAAGTATCAGGATTACAATACAGGGTATTTAGAATACATTTACCTGACC

              200       210       220       230       240       250       260       270       280
6a     CTAACAAATTTGCATTGCCTGACTCGTCTCTTTTTGATCCCACAACACAACGTTTGGTATGGGCATGCACAGGCCTAGAGGTGGGCAGGGGACAGCCA
16     CCAATAAGTTTGGTTTTCCTGACACCTCATTTTATAATCCAGATACACAGCGGCTGGTTTGGGCCTGTGTAGGTGTTGAGGTAGGTCGTGGTCAGCCA

     290       300
6a     TTAGGTGTGGGTGTAAG
16     TTAG.............
```

```
                                                                1        10        20        30        40
6a    ......................................................CCTAACCCTGTATCCAAAGTTGTTGCCACGGATGCTTATGTTAC
18    CACTGGGCTAAAGGCACTGCTTGTAAATCGCGTCCTTTATCACAGGGCGATTGCCCCCCTTTAGAACTTAAAAACACAGTTTTGGAAGATGGTGATAT

           50        60             70         80        90        100       110       120       130
6a    ..TCGCACCAACATATTTTATCATG....CCAGCA.GTTCTAGACTTCTTGCAGTGGGTCATCCTTATTTTCCATAAAACGGGCTAACAAAA..CTG
18    GGTAGATACTGGATATGGTGCCATGGACTTTAGTACATTGCAAGATACTAAATGTGAGGTACCATTGGATATTTGTCAGTCTATTTGTAAATATCCTG

         140       150       160       170       180       190          200       210       220
6a    TTGTGCCAAAGGTGTCAGGATATCAATACAGGGTATTTAAGGTGGTGTTACCAGATCCTAACAAA...TTTGCATTGCCTGACTCGTCTCTTTTTGAT
18    ATTATTTACAAATGTCTGCAGATCCTTATGGGGATTCCATGTTTTTTGCTTACGGCGTGAGCAGCTTTTTGCTAGGCATTTTTGGAATAGAGCAGGT

      230           240       250       260        270       280       290       300
6a    CCCACA....ACACAACGTTTGGTATGGGCATGCAC.AGGCCTAGAGGTGGGCAGGGACAGCCATTAGGTGTGGGTGTAAG................
18    ACTATGGGTGACACTGTGCCTCA.ATCCTTATATATTAAAGGCACAGGTATGCGTGCTTCACCTGGCAGCTGTGTGTATTCTCCCTCTCCAAGTGGCT

6a    ..........................
18    CTATTGTTACCTCTGACTCCCAGTTG
```

```
      1        10        20        30        40        50        60
6a    CCTAACCCTGTATCCAAAGTTGTTGCCACGGATGCTTATGTTACTCGCACCAACATATTT
31    .CTGTCCCAGTGTCTAAAGTTGTAAGCACGGATGAATATGTAACACGAACCAACATATAT

               70        80        90       100       110
6a    TATCATGCCAGCAGTTCTAGACTTCTTGCAGTGGGTCATCCTTATTTTTCCATA...AAA

31    TATCACGCCAGGCAGTGCTAGGCTGCTTACAGTAGGCCATCCATATTATTCCATACCTAAA

      120              130       140       150       160       170
6a    CGGG......CTAACAAAACTGTTGTGCCAAAGGTGTCAGGATATCAATACAGGGTATTT
31    TCTGACAATCCTAAAAAAATAGTTGTACCAAAGGTGTCAGGATTACAATATAGGGTATTT

            180       190       200       210       220       230
6a    AAGGTGGTGTTACCAGATCCTAACAAATTTGCATTGCCTGACTCGTCTCTTTTTGATCCC
31    AGGGTTCGTTTACCAGATCCAAACAAATTTGGATTTCCTGATACATCTTTTTATAATCCT

            240       250       260       270       280       290
6a    ACAACACAACGTTTGGTATGGGCATGCACAGGCCTAGAGGTGGGCAGGGGACAGCCATTA
31    GAAACTCAACGCTTAGTTTGGGCCTGTGTTGGTTTAGAGGTAGGTCGCGGGCAGCCATTA

            300
6a    GGTGTGGGTGTAAG..
31    GGTGTAGGTATTAGTG
```

```
     1        10        20        30        40        50        60
6a   CCTAACCCTGTATCCAAAGTTGTTGCCACGGATGCTTATGTTACTCGCACCAACATATTT
33   ............CTTGAAATAGGTAGAGGGCAGCCATTAGGCGTTGGCATAAGTGGTCAT

              70        80        90       100       110       120
6a   TATCATGCCAGCAGTTCTAGACTTCTTGCAGTGGGTCATCCTTATTTTTCCATAAAACGG
33   CCTTTATTAAACAAATTTGATGACACTGAAACCAGTAACAAGTATCCTGGACAACCGGGT

             130       140       150       160       170       180
6a   GCTAACAAAACTGTTGTGCCAAAGGTGTCAGGATATCAATACAGGGTATTTAAGGTGGTG
33   GCTGATAATAGGGA.ATGTTTATCCATGGATTATAAACAAACACAGTTATGTTTACTTGG

             190       200       210       220       230       240
6a   TTACCAGATCCTAACAAATTTGCATTGCCTGACTCGTCTCTTTTTGATCCCACAACACAA
33   ATGTAAGCCTCCAACAGGGGAACATTGGGGTAAAGGTGTTGCTTGTAC....TAATGCAG

             250       260       270       280       290       300
6a   CGTTTGGTATGGGCATGCACAGGCCTAGAGGTGGGCAGGGGACAGCCATTAGGTGTGGGT
33   CACCTGCCAATGAT.TGTCCACCTTTAGAACTTATAAA..TACTATTATTGAGG......

6a   GTAAG
33   .....
```

```
               1         10        20        30        40        50
6a      .CCTAACCCTGTATCCAAAGTTGTTGCCACGGATGCTTA..TGTTACTCGCACCA.ACAT
45a     CCCTTCTCCCAGTGGCTCTATTATTACTTCTGATTCTCAATTATTTAATAAGCCATATTG

           60        70        80        90       100       110
6a      ATTTTATCATGCCAGCAGTTCTAGACTTCTTGCAGTGGGTCATCCTTATTTTTCCATAAA
45a     GTTACATAAGGCCCAGGGCCATAACAATGGTATTTGTTGGCATAATCAGTTGTTTGTTAC

          120       130       140       150       160       170
6a      ACGGGCTAACAAAACTGTTGTGCCAAAGGTGTCAGGATAT..CAATACAGGGTATTTAAG
45a     TGTAGTGGACACTACCCGCAGTACTAATTTAACATTATGTGCCTCTACACAAAATTCTGT

            180       190       200       210       220       230
6a      GTGGTGT.TACCAGATCCTAACAAATTTGCATTGCCTGACTCGTCTCTTTTTGATCCCAC
45a     GCCAAGTACATATGACCCTACTAAGTTTAAGCAGTATAGTAGACATGTGGAGGAATATGA

             240       250       260       270       280       290
6a      AACACAACGTTTGGTATGGGCATGCACAGGCCTAGAGGTGGGCAGGGGACAGCCATTAGG
45a     TTTACAGTTTATTTTTCAGTTGTGCACTATTACTTTAACTGCAGAGGTTATGTCATATAT

             300
6a      TGTGGGTGTAAG................................................
45a     CCATAGTATGAATAGTAGTATATTAGAAAATTGGAATTTTGGTGTCCCTCCACCACCTAC

6a      ...........
45a     TACAAGTTTGG
```

```
       1        10        20        30        40        50
6a     CCTAACCCTGTATCCAAAGTTGTTGCCACGGATG..CTTATGTTACTCGCACCAACATAT
45b    .......CAGGATACAAAGTGCGAGGTTCCATTAGACATTTGTCAATCCATCTGTAAATA

        60        70        80        90       100       110
6a     TTTATCATGCCAGCAGTTCTAGACTTCTTGCAGTGGGTCATCCTTATTTTTCCATAAAAC
45b    TCCAGATTATTTGCAAATGTCTGCTGATCCCTATGGGGATTCTATGTTTTTTTGCCTACG

       120       130       140       150       160       170
6a     GGGCTAACAAAACTGTTGTGCCAAAGGTGTCAGGATATCAATACAGGGTATTTAAGGTGG
45b    CCGTGAACAA..CTGTT.TGCAAGACATTTTGGA.......ATAGGGCAGGTGTTATGG

       180       190       200       210       220       230
6a     TGTTACCAGATCCTAACAAATTTGCATTGCCTGACTCGTCTCTTTTTGATCCCACAACAC
45b    .GTGACACAGTACCTACGGACCTATATATTAAAGGCACTAGCGCTAATATGCGTGAAACC

       240       250       260       270       280       290
6a     AACGTTTGGTATGGGCATGCACAGGCCTAGAGGTGGGCAGGGGACAGCCATTAGGTGTGG
45b    CCTGGCAGTTGTGTGTATTCCCCT.TCTCCCAGTGGCTCTATTATTACTTCTG.......

       300
6a     GTGTAAG
45b    .......
```

```
                         1        10        20        30        40
6a      ...............CCTAACCCTGTATCCAAAGTTGTTGCCACGGATGCTTATGTTACT
45c     CAACCTGGTGACTGTCCTCCTTTGGAACTTAAAAACACCATTATTGAGGATGGTGATAT.

           50        60        70        80        90       100
6a      CGCACCAACATATTTTATCATGCCAGCAGTTCTAGACTTCTTGCAGTGGGTCATCCTTAT
45c     ..GGTGGATACAGGTTATGGGGCAATGGATTTTAG.TACATTGCAGGATACAAAGTGCGA

          110       120       130       140       150       160
6a      TTTTCCATAAAACGGGCTAACAAAACTGTTGTGCCAAAGGTGTCAGGATATCAATACAGG
45c     GGTTCCATTAGACAT.TTGTCAATCCATCTGTAAATATCCAGATTATTTGCAAATGTCTG

          170       180       190       200       210       220
6a      GTATTTAAGGTGGTGTTACCAGATCCTAACAAATTTGCATTGCCTGACTCGTCTCTTTTT
45c     CTGATCCCTATGGGGATTCTATGTTTTTTTGCCTACGCCGTGAACAACTGTTTGCAAGAC

          230       240       250       260        270       280
6a      GATCCCACAACACAACGTTTGGTATGGGCATGCACAG.GCCTAGAGGTGGGCAGGGGACA
45c     ATTTTTGGAATAGGGCAGGTGTTATGGGTG.ACACAGTACCTACGGACCTATATATTAAA

           290       300
6a      GCCATTAGGTGTGGGTGTAAG.......................................
45c     GGCACTAG.CGCTAATATGCGTGAAACCCCTGGCAGTTGTGTGTATTCCCCTTCTCCCAG

6a      ....................
45c     TGGCTCTATTATTACTTCTG
```

```
      1       10        20        30        40        50        60
6a    CCTAACCCTGTATCCAAAGTTGTTGCCACGGATGCTTATGTTACTCGCACCAACATATTT
52    ......CCTGTCTCTAAGGTTGTAAGCACTGATGAGTATGTGTCTCGCACAAGCATCTAT

              70        80        90       100       110       120
6a    TATCATGCCAGCAGTTCTAGACTTCTTGCAGTGGGTCATCCTTATTTTTCCATAAAACGG
52    TATTATGCAGGCAGTTCTCGATTACTAACAGTAGGACATCCCTATTTTTCTATTAAAAAC

                        130       140       150       160
6a    GC...............TAACAAAACTGTTGTGCCAAAGGTGTCAGGATATCAATACAGG
52    ACCAGTAGTGGTAATGGTAAAAAAGTTTTAGTTCCCAAGGTGTCTGGCCTGCAATACAGG

         170       180       190       200       210       220
6a    GTATTTAAGGTGGTGTTACCAGATCCTAACAAATTTGCATTGCCTGACTCGTCTCTTTTT
52    GTATTTAGAATTAAATTGCCGGACCCTAATAAATTTGGTTTTCCAGATACATCTTTTTAT

         230       240       250       260       270       280
6a    GATCCCACAACACAACGTTTGGTATGGGCATGCACAGGCCTAGAGGTGGCAGGGGACAG
52    AACCCAGAAACCCAAAGGTTGGTGTGGGCCTGTACAGGCTTGGAAATTGGTAGGGGACAG

         290       300
6a    CCATTAGGTGTGGGTGTAAG....
52    CCTTTAGGTGTGGGTATTAGTGGG
```

```
                                                       1         10        20
                                                       .         .         .
6b   .......................................CAGCCATTAGGTGTGGGTGTA
11a  CCAAGGTTGTTGCCACGGATGCGTATGTTAAACGCACCAACATATTTTATCATGCCAGCA

             30        40        50        60        70
             .         .         .         .         .
6b   AGTGGACATCCTTTCCT.....AAATAAATATGATGATGTTGAAAA..TTCAGGGAGTGG
11a  GTTCTAGACTCCTTGCTGTGGGACATCCATATTACTCTATCAAAAAAGTTAACAAAACAG

          80        90       100       110       120       130
          .         .         .         .         .         .
6b   TGGTAACCCTGGA..CAGGATAACAGGGTTAATGTTGGTATGGATTATAAACAAACACAA
11a  TTGTACCAAAGGTGTCTGGATATCAATATAGAGTGTTTAAGGTAGTGTTGCCAGATCCTA

           140       150       160       170       180       190
             .         .         .         .         .         .
6b   TTATGCATGGTTGGATGTGCCCCCCCTTTGGGCGAGCATTGGGGTAAAGGTAAACAGTGT
11a  ACAAGTTTGCATT.ACCTGATTCATCTCTGTTTGACCCCACTACACAGCGTTTAGTATGG

           200       210       220       230       240
             .         .         .         .         .
6b   ACTAATACACCTGTACAGGCTGGTGACTGCCCGCCCTTAGAACTTATTACCAG.......
11a  GCGTGCACAGGGTTGGAGGTAGGCAGGGGTCAACCTTTAGGCGTTGGTGTTAGTGGGCAT

6b   ............................................................
11a  CCATTGCTAAACAAATATGATGATGTAGAAAATAGTGGTGGGTATGGTGGTAATCCTGGT

6b   ...
11a  CAG
```

```
            1        10        20        30        40        50        60
6b          CAGCCATTAGGTGTGGGTGTAAGTGGACATCCTTTCCTAAATAAATATGATGATGTTGAA
11b         ...CCTTTAGGCGTTGGTGTTAGTGGGCATCCATTGCTAAACAAATATGATGATGTAGAA

                   70        80        90       100       110
6b          AATTCAGGGAGG...TGGTGGTAACCCTGGACAGGATAACAGGGTTAATGTTGGTATGGAT
11b         AATAGTGGTGGGTATGGTGGTAATCCTGGTCAGGATAATAGGGTTAATGTAGGTATGGAT

            120       130       140       150       160       170
6b          TATAAACAAACACAATTATGCATGGTTGGATGTGCCCCCCCTTTGGGCGAGCATTGGGGT
11b         TATAAACAAACCCAGCTATGTATGGTGGGCTGTGCTCCACCGTTAGGTGAACATTGGGGT

            180       190       200       210       220       230
6b          AAAGGTAAACAGTGTACTAATACACCTGTACAGGCTGGTGACTGCCCGCCCTTAGAACTT
11b         AAGGGTACACAATGTTCAAATACCTCTGTACAAAATGGTGACTGCCCCCCGTTGGAACTT

            240
6b          ATTACCAG....................................................
11b         ATTACCAGTGTTATACAGGATGGGGACATGGTTGATACAGGCTTTGGTGCTATGAATTTT

6b          ............................
11b         GCAGACTTACAAACCAATAAATCGGATG
```

```
6b   ............................................................
16   CTGTCCCAGTATCTAAGGTTGTAAGCACGGATGAATATGTTGCACGCACAAACATATATT

             1        10        20        30        40
6b   ........CAGCCATTAGGTGTGGGTGTAAGTGGACATCC...TTTCCTAAATAAATATG
16   ATCATGCAGGAACATCCAGACTACTTGCAGTTGGACATCCCTATTTTCCTATTAAAAAAC

     50        60        70        80        90        100
6b   ATGATGTTGAAAATTCAGGGAGTGGTGGTAACCCTGGACAGGATAACAGGGTTAATGTTG
16   CTAACAATAACAAAATATTAGTTCCTAAAGTATCAGGATTACAATACAGGGTAT...TTA

     110       120       130       140       150       160
6b   GTATGGATTATAAACAAACACAATTATGCATGGTTGGATGTGCCCCCCTTTGGGCGAGC
16   GAATACATT..TACCTGACCCCAATAAGTTTGGTTTTCCTGACACCTCATTTTATAATCC

     170       180       190       200       210       220
6b   ATTGGGGTAAAGGTAAACAGTGTACTAATACACCTGTACAGGCTGGTGACTGCCCGCCCT
16   AGATACACAGCGGCTGGTT.TGGGCCTGTGTAGGTGTTGAGGTAGGTCGTGGTCAGCCAT

     230       240
6b   TAGAACTTATTACCAG
16   TAG.............
```

```
        1         10        20        30        40        50
6b    ....CAGCCATTAGGTGTGGGTGTAAGTGGACATCCTTTCCTAAATAAATATGATGATGT
18    CACTGGGCTAAAGGCACTGCTTGTAAATCG.CGTCCTTTATCACAGGGCGATTGCCCCCC

       60        70        80        90        100       110
6b    TGAAAATTCAGGGAGTGGTGGTAACCCTGGACAGGATAACAGGGTTAATGTTGG.TATGG
18    TTTAGAACTTAAAAACACAGTT...TTGGAAGATGGTGATATGGTAGATACTGGATATGG

        120       130       140       150       160       170
6b    .ATTATAAACAAACACA.ATTATGCATGGTTGGATGTGCCCCCCCTTTGGGCGAGCATTG
18    TGCCATGGACTTTAGTACATTGCAAGATACTAAATGTGAGGTACCATTGGATATTTGTCA

        180       190       200       210       220       230
6b    GGGTAAAGGTAAACAGTGTACTAATACACCTGTACAGGCTGGTGACTGCCCGCCCTTAGA
18    GTCTATTTGTAAATATCCTGATTATTTACAAATGTCTGCAGATCCTTATGGGGATTCCAT

        240
6b    ACTTATTACCAG................................................
18    GTTTTTTTGCTTACGGCGTGAGCAGCTTTTTGCTAGGCATTTTTGGAATAGAGCAGGTAC

6b    ............................................................
18    TATGGGTGACACTGTGCCTCAATCCTTATATATTAAAGGCACAGGTATGCGTGCTTCACC

6b    ............................................................
18    TGGCAGCTGTGTGTATTCTCCCTCTCCAAGTGGCTCTATTGTTACCTCTGACTCCCAGTT

6b    .
18    G
```

```
6b    ............................................................
31    CTGTCCCAGTGTCTAAAGTTGTAAGCACGGATGAATATGTAACACGAACCAACATATATT

                1         10         20             30        40
6b    ..........CAGCCATTAGGTGTGGGTGTAAG....TGGACATCCTTTCCTAAATAAAT
31    ATCACGCAGGCAGTGCTAGGCTGCTTACAGTAGGCCATCCATATTATTCCATACCTAAAT

        50         60        70        80        90        100
6b    ATGATGATGTTGAAAATTCAGGGAGTGGTGGTAACCCTGGACAGGATAACAGGGTTAATG
31    CTGACAATCCTAAAAA....AATAGTTGTACCAAAGGTGT.CAGGATTACAATAT.AGGG

       110       120        130       140       150       160
6b    TTGGTATGGATTATAAAC.AAACACAATTATGCATGGTTGGATGTGCCCCCCCTTTGGGC
31    TATTTAGGGTTCGTTTACCAGATCCAAACAAATTTGGATTTCC.TGATACATCTTTTTAT

        170       180       190       200       210       220
6b    GAGCATTGGGGTAAAGGTAAACAGTGTACTAATACACCTGTACAGGCTGGTGACTGCCCG
31    AATCCTGAAACTCAACGCTTAGTTTGGGCCTGTGTTGGTTTAGAGGTAGGTCGCGGGCAG

        230       240
6b    CCCTTAGAACTTATTACCAG..
31    CCATTAGGTGTAGGTATTAGTG
```

```
                        1       10        20        30        40
6b    ..................CAGCCATTAGGTGTGGGTGTAAGTGGACATCCTTTCCTAAAT
33    CTTGAAATAGGTAGAGGGCAGCCATTAGGCGTTGGCATAAGTGGTCATCCTTTATTAAAC

            50        60           70        80        90
6b    AAATATGATGATGTTGAAA...ATTCAGGGAGTGGTGGTAACCCTGGACAGGATAACAGG
33    AAATTTGATGACACTGAAACCAGTAACAAGTATCCTGGACAACCGGGTGCTGATAATAGG

    100       110       120       130       140       150
6b    GTTAATGTTGGTATGGATTATAAACAAACACAATTATGCATGGTTGGATGTGCCCCCCCT
33    GAATGTTTATCCATGGATTATAAACAAACACAGTTATGTTTACTTGGATGTAAGCCTCCA

    160       170       180       190       200       210
6b    TTGGGCGAGCATTGGGGTAAAGGTAAACAGTGTACTAATACACCTGTACAGGCTGGTGAC
33    ACAGGGGAACATTGGGGTAAAGGTGTTGCTTGTACTAATGCA...GCACCTGCCAATGATT

    220       230       240
6b    TGCCCGCCCTTAGAACTTATTACCAG...........
33    TGTCCACCTTTAGAACTTATAAATACTATTATTGAGG
```

```
6b     ............................................................
45a    CCCTTCTCCCAGTGGCTCTATTATTACTTCTGATTCTCAATTATTTAATAAGCCATATTG

                                                     1        10
6b     ..............................................CAGCCATTAGGTGT
45a    GTTACATAAGGCCCAGGGCCATAACAATGGTATTTGTTGGCATAATCAGTTGTTTGTTAC

          20          30        40        50              60
6b     GGGTGTAAGTGG.ACATCCTTTCCTAAATAAATATGATGA.....TGTTGAAAATTCAGG
45a    TGTAGTGGACACTACCCGCAGTACTAATTTAACATTATGTGCCTCTACACAAAATTCTGT

         70        80        90        100       110       120
6b     G...AGTGGTGGTAACCCTGGACAGGATAACAGGGTTAATGTTGGTATGGATTATAAACA
45a    GCCAAGTACATATGACCCTACTAAGTTTAAGCAGTATAGTAGACATGTGGAGGAATATGA

        130       140       150       160         170       180
6b     AACACAATTATGCATGGTTGGATGTGCCCCCCCTTTGG..GCGAGCATTGGGGTAAAGGT
45a    TTTACAGTTTATTTTTCAGTTGTGCACTATTACTTTAACTGCAGAGGTTATGTCATATAT

          190           200       210       220       230
6b     AAACAGTGT....ACTAATACACCTGTACAGGCTGGTGACTGCCCGCCCTTAGAACTTAT
45a    CCATAGTATGAATAGTAGTATATTAGAAAATTGGAATTTTGGTGTCCCTCCACCACCTAC

       240
6b     TACCAG.....
45a    TACAAGTTTGG
```

```
                1        10        20        30        40        50
6b   .CAGCCATTAGGTGTG.GGTGTAAGTGGACATCCTTTCTAAATAAATATGATGATGTTG
45b  CAGGATACAAAGTGCGAGGTTCCATTAGACAT..TTGTCAATCCATCTGTAAATATCCAG

     60        70        80        90        100       110
6b   AAAATTCAGGGAGTGGTGGTAACCTGGACAGGATAACAGGTTA.......ATGTTGGT
45b  ATTATTTGCAAATGTCTGCTGATCCCTATGGGGATTCTATGTTTTTTGCCTACGCCGTG

        120       130       140       150       160       170
6b   ATGGATTATAAACAA.ACACAATTATGCATGGTTGGATGTGCCCCCCCTTTGGGCGAGCA
45b  AACAACTGTTTGCAAGACATTTTTGGAATAGGGCAGGTGTTATGGGTGACACAGTACCTA

         180       190           200       210       220
6b   TTGGGGTAAAGGTAAACAGT....GTACTAATACACCTGTACAGGCTGGTGACTGCCCGC
45b  CGGACCTATATATTAAAGGCACTAGCGCTAATATGCGTGAAACCCCTGGCAGTTGTGTGT

      230       240
6b   CCTTAGAACTTATTACCAG.................
45b  ATTCCCCTTCTCCCAGTGGCTCTATTATTACTTCTG
```

```
                                                 1        10        20
6b    .....................................CAGCCATTAGGTGTGGGTGTA.A
45c   CAACCTGGTGACTGTCCTCCTTTGGAACTTAAAAACACCATTATTGAGGATGGTGATATG

           30        40        50        60        70
6b    GTGGACATCCTTTCCTA...AATAAATATGATGATGTTGAAAATTCAGGAGTGGTGGTA
45c   GTGGATACAGGTTATGGGGCAATGGATTTTAGTACATTGCAGGATACAAAGTGCGAGGTT

     80        90        100       110       120       130
6b    ACCCTGGACAGGATAACAGGGTTAATGTTGGTAT..GGATTATAAACAAACACAATTATG
45c   CCATTAGACATT.TGTCAATCCATCTGTAAATATCCAGATTATTTGCAAATGTCTGCTGA

      140       150       160       170          180       190
6b    CATGGTTGGATGTGCCCCCCCTTTGGGCGAGCATTG.GGGTAAAGGTAAACAGTGTACTA
45c   TCCCTATGGGGATTCTATGTTTTTTTGCCTACGCCGTGAACAACTGTTTGCAAGACATTT

       200       210            220       230       240
6b    ATACACCTGTACAGGCT.....GGTGACTGCCCGCCCTTAGAACTTATTACCAG......
45c   TTGGAATAGGGCAGGTGTTATGGGTGACACAGTACCTACGGACCTATATATTAAAGGCAC

6b    ............................................................
45c   TAGCGCTAATATGCGTGAAACCCCTGGCAGTTGTGTGTATTCCCCTTCTCCCAGTGGCTC

6b    ..............
45c   TATTATTACTTCTG
```

```
6b      ............................................................
52      CCTGTCTCTAAGGTTGTAAGCACTGATGAGTATGTGTCTCGCACAAGCATCTATTATTAT

             1         10         20         30         40         50
6b      .....CAGCCATTAGGTGTGGGT.GTAAGTGGACATCCTTTCCTAAATAAATATGATGAT
52      GCAGGCAGTTCTCGATTACTAACAGTAGGACATCCCTATTTTTCTATTAAAAACACCAGT

             60         70         80         90        100        110
6b      GTTGAAAAT..TCAGGGAGTGGTGGTAACCCTGGACAGGATAACAGGGTTAATGTTGGTA
52      AGTGGTAATGGTAAAAAAGTTTTAGTTCCCAAGGTGTCTGGCCTGCAATACAGGGTATTT

              120        130        140        150        160        170
6b      TGGATTATA..AACAAACACAATTATGCATGGTTGGATGTGCCCCCCCTTTGGGCGAGCA
52      AGAATTAAATTGCCGGACCCTAATAAATTTGGTTTTCCAGATACATCTTTTTATAACCCA

                180        190        200        210        220        230
6b      TTGGGGTAAAGGTAAACAGTGTACTAATACACCTGTACAGGCTGGTGACTGCCCGCCCTT
52      GAAACCCAAAGGTTGGT.GTGGGCCTGTACAGGCTTGGAAATTGGTAGGGGACAGCCTTT

                240
6b      AGAACTTATTACCAG....
52      AGGTGTGGGTATTAGTGGG
```

```
                 1       10        20        30           40
6b    ...........CAGCCATTAGGTGTGGGTGTAAGTGGACATCCTTTCC...TAAATAAAT
58    CTCCTGTGCCTGTGTCTAAGGTTGTAAGCACTGATGAATATGTGTCACGCACAAGCATTT

        50        60        70        80        90       100
6b    ATGATGATGTTGAAAATTCAGGGAGTGGTGGTAACCCTGGACAGGATAACAGGGTTAATG
58    ATTATTATGCTGGCAGTTCCCGACTTTTGGCTGTTGGCAATCCATATTTTTCCATCAAGA

       110       120       130       140       150       160
6b    TTGGTATGGATTATAAACAAACACAATTATGCATGGTTGGATGTGCCCCCCCTTTGGGCG
58    GTCCCAATAACAATAAAAAAGTATTAGTTCCCAAGGTATCAGGCTTACAGTATAGGGTCT

       170       180       190          200       210       220
6b    AGCATTGGGGTAAAGGTAAACAGTGTA...CTAATACACCTGTACAGGCTGGTGACTGCC
58    TTAGGGTGCGTTTACCTGATCCCAATAAATTTGGTTTTCCTGATACATCTTTTTATAACC

          230       240
6b    CGCCCTTAGAACTTATTACCAG......................................
58    CTGATACACAACGTTTGGTCTGGGCATGTGTAGGCCTTGAAATAGGTAGAGGACAGCCAT

6b    ...
58    TGG
```

```
                     1         10        20        30        40
11a   ............CCAAGGTTGTTGCCACGGATGCGTATGTTAAACGCACCAACATATTTT
16    CTGTCCCAGTATCTAAGGTTGTAAGCACGGATGAATATGTTGCACGCACAAACATATATT

      50         60        70        80        90        100
11a   ATCATGCCAGCAGTTCTAGACTCCTTGCTGTGGGACATCCATATTACTCTATCAAAAAAG
16    ATCATGCAGGAACATCCAGACTACTTGCAGTTGGACATCCCTATTTTCCTATTAAAAAAC

     110             120       130       140       150       160
11a   TTAACAA......AACAGTTGTACCAAAGGTGTCTGGATATCAATATAGAGTGTTTAAGG
16    CTAACAATAACAAAATATTAGTTCCTAAAGTATCAGGATTACAATACAGGGTATTTAGAA

            170       180       190       200       210       220
11a   TAGTGTTGCCAGATCCTAACAAGTTTGCATTACCTGATTCATCTCTGTTTGACCCCACTA
16    TACATTTACCTGACCCCAATAAGTTTGGTTTTCCTGACACCTCATTTTATAATCCAGATA

            230       240       250       260       270       280
11a   CACAGCGTTTAGTATGGGCGTGCACAGGGTTGGAGGTAGGCAGGGGTCAACCTTTAGGCG
16    CACAGCGGCTGGTTTGGGCCTGTGTAGGTGTTGAGGTAGGTCGTGGTCAGCCATTAG...

            290       300       310       320       330       340
11a   TTGGTGTTAGTGGGCATCCATTGCTAAACAAATATGATGATGTAGAAAATAGTGGTGGGT
16    ............................................................

            350       360
11a   ATGGTGGTAATCCTGGTCAG
16    ....................
```

```
                                                  1         10
11a   .........................................CCAAGGTTGTTGCCACGGA
18    CACTGGGCTAAAGGCACTGCTTGTAAATCGCGTCCTTTATCACAGGGCGATTGCCCCCCT

      20        30        40        50          60          70
11a   TGCGTATGTTAAACGCACCAACATATTTTATCATGCCA..GCAGTT.CTAGACTCCTTGC
18    TTAGAAC.TTAAAAACACAGTTTTGGAAGATGGTGATATGGTAGATACTGGATATGGTGC

        80        90        100       110       120       130
11a   TGTGGGACATCCATATTACTCTATCAAAAAAGTTAACAAAACAGTTGTACCAAAGGT.GT
18    CATGG.ACTTTAGTACATTGCAAGATACTAAATGTGAGGTACCATTGGATATTTGTCAGT

        140       150       160       170       180       190
11a   CTGGATATCAATATAGAGTGTTAAGGTAGTGTTGCCAGATCCTAACAAGTTTGCATTAC
18    CTATTTGTAAATATCCTGATTATTTACAAATGTCTGCAGATCCTTATGGGGATTCCATGT

        200       210       220       230       240       250
11a   CTGATTCATCTCTGTTTGACCCACTACACAGCGTTTAGTATGGCGTGCACAGGGT...
18    TTTTTGCTTACGGCGTGAGCA.GCTTTTTGCTAGGCATTTTTGGAATAGAGCAGGTACT

            260       270       280        290       300
11a   .TGGAGGTAGGCAGGGGTCAACCTTTAGGCGTT...GGTGTTAGTGGGCATCCATTGCTA
18    ATGGGTGACACTGTGCCTCAATCCTTATATATTAAAGGCACAGGTATGCGTGCTTCACCT

      310       320       330       340       350       360
11a   AACAAATATGATGATGTAGAAAATAGTGGTGGGTATGGTGGTAATCCTGGTCAG......
18    GGCAGCTGTGTGTATTCTCCCTCTCCAAGTGGCTCTATTGTTACCTCTGACTCCCAGTTG
```

```
                        1         10        20        30        40
11a   ............CCAAGGTTGTTGCCACGGATGCGTATGTTAAACGCACCAACATATTTT
31    CTGTCCCAGTGTCTAAAGTTGTAAGCACGGATGAATATGTAACACGAACCAACATATATT

      50        60        70        80        90        100
11a   ATCATGCCAGCAGTTCTAGACTCCTTGCTGTGGACATCCATATTACTCTAT........
31    ATCACGCAGGCAGTGCTAGGCTGCTTACAGTAGGCCATCCATATTATTCCATACCTAAAT

                110       120       130       140       150
11a   .CAAAAAAGTTAACAAAACAGTTGTACCAAAGGTGTCTGGATATCAATATAGAGTGTTTA
31    CTGACAATCCTAAAAAAATAGTTGTACCAAAGGTGTCAGGATTACAATATAGGGTATTTA

     160       170       180       190       200       210
11a   AGGTAGTGTTGCCAGATCCTAACAAGTTTGCATTACCTGATTCATCTCTGTTGACCCA
31    GGGTTCGTTTACCAGATCCAAACAAATTTGGATTTCCTGATACATCTTTTTATAATCCTG

     220       230       240       250       260       270
11a   CTACACAGCGTTTAGTATGGGCGTGCACAGGGTTGGAGGTAGGCAGGGGTCAACCTTTAG
31    AAACTCAACGCTTAGTTTGGGCCTGTGTTGGTTTAGAGGTAGGTCGCGGGCAGCCATTAG

     280       290       300       310       320       330
11a   GCGTTGGTGTTAGTGGGCATCCATTGCTAAACAAATATGATGATGTAGAAAATAGTGGTG
31    GTGTAGGTATTAGTG.............................................

     340       350       360
11a   GGTATGGTGGTAATCCTGGTCAG
31    .......................
```

```
        1        10        20        30        40        50        60
        .         .         .         .         .         .         .
11a     CCAAGGTTGTTGCCACGGATGCGTATGTTAAACGCACCAACATATTTTATCATGCCAGCA
33      ............................................CTTGAAATAGGTAGAG

                 70        80        90       100       110       120
                  .         .         .         .         .         .
11a     GTTCTAGACTCCTTGCTGTGGGACATCCATATTACTCTATCAAAAAAGTTAACAAAACAG
33      GGCAGCCATTAGGCGTTGGCATAAGTGGTCATC.CTTTATTAAACAAATTTG.ATGACAC

                130       140       150       160       170       180
                  .         .         .         .         .         .
11a     TTGTACCAAAGGTGTCTGGATATCAATATAGAGTGTTTAAGGTAGTGTTGCCAGATCCTA
33      TGAAACCAGTAACAAGTATCCTGGACAACCGGGTGCTGATAATAGGGAATGTTTATCC..

                190       200       210       220       230       240
                  .         .         .         .         .         .
11a     ACAAGTTTGCATTACCTGATTCATCTCTGTTTGACCCACTACACAGCGTTTAGTATGGG
33      ATGGATT...ATAAACAAACACAGTTATGTTT.ACTTGGATGTA.AGCCTCCAACAGGGG

                250       260       270       280       290       300
                  .         .         .         .         .         .
11a     CGTGCACAGGGTTGGAGGTAGGCAGGGGTCAACCTTTAGGCGTTGGTGTTAGTGGGCATC
33      AACATTGGGGTAAAGGTGTTGCTTGTACTAATGCAGCACCTGCCAATGATTGTCCACCTT

                310       320       330       340       350       360
                  .         .         .         .         .         .
11a     CATTGCTAAACAAATATGATGATGTAGAAAATAGTGGTGGGTATGGTGGTAATCCTGGTC
33      TAGAACTTA.TAAATACTATTATTGAGG................................

11a     AG
33      ..
```

```
                    1         10        20        30        40
11a   ..............CCAAGGTTGTTGCCACGGATGCGTA..TGTTAAACGCACCA.ACAT
45a   CCCTTCTCCCAGTGGCTCTATTATTACTTCTGATTCTCAATTATTTAATAAGCCATATTG

           50        60        70        80        90       100
11a   ATTTTATCATGCCAGCAGTTCTAGACTCCTTGCTGTGGACATCCATATTACTCTATCAA
45a   GTTACATAAGGCCCAGGGCCATAACAATGGTATTTGTTGGCATAATCAGTTGTTTGTTAC

          110       120       130       140       150
11a   AAAAGTTAACAAAACA.GTTGTACCAA...AGGTGTCTGGATATCAATATAGAGTGTTTA
45a   TGTAGTGGACACTACCCGCAGTACTAATTTAACATTATGTGCCTCTACACAAAAT.TCTG

    160       170       180       190       200       210
11a   AGGTAGTGTTGCCAGATCCTAACAAGTTTGCATTACCTGATTCATCTCTGTTTGACCCCA
45a   TGCCAAGTACATATGACCCTACTAAGTTTAAGCAGTATAGTAGACATGTGGAGGAATATG

    220       230       240       250       260       270
11a   CTACACAGCGTTTAGTATGGGCGTGCACAGGGTTGGAGGTAGGCAGGGGTCAACCTTTAG
45a   ATTTACAGTTTATTTTTCAGTTGTGCACTATTACTTTAACTGCAGAGGTTATGTCATATA

    280       290       300       310       320       330
11a   GCGTTGGTGTTAGTGGGCATCCATTGCTAAACAAATATGATGATGTAGAAAATAGTGGTG
45a   TCCATAGTATGAATAGTAGTATATTAGAAAATTGGAATTTTGGTGTCCCTC.CACCACCT

    340       350       360
11a   GGTATGGTGGTAATCCTGGTCAG
45a   ACTACAAGTTTGG..........
```

```
     1        10        20        30        40        50        60
11a  CCAAGGTTGTTGCCACGGATGCGTATGTTAAACGCACCAACATATTTTATCATGCCAGCA
45b  ............................................................

              70        80        90       100       110       120
11a  GTTCTAGACTCCTTGCTGTGGGACATCCATATTACTCTATCAAAAAAGTTAACAAAACAG
45b  ...............CAGGATACAAAGTGCGAGGTTCCATTAGACAT.TTGTCAATCCAT

             130       140       150       160       170       180
11a  TTGTACCAAAGGTGTCTGGATATCAATATAGAGTGTTTAAGGTAGTGTTGCCAGATCCTA
45b  CTGTAAATATCCAGATTATTTGCAAATGTCTGCTGATCCCTATGGGGATTCTATGTTTTT

             190       200       210       220       230       240
11a  ACAAGTTTGCATTACCTGATTCATCTCTGTTTGACCCCACTACACAGCGTTTAGTATGGG
45b  TTGCCTACGCCGTGAACAACTGTTTGCAAGACATTTTTGGAATAGGGCAGGTGTTATGGG

             250       260       270       280       290       300
11a  CGTGCACAGGGTTGGAGGTAGGCAGGGGTCAACCTTTAGGCGTTGGTGTTAGTGGGCATC
45b  TG.ACACAGTACCTACGG.ACCTATATATTAA.....AGGCACTAGCGCTAATATGCGTG

             310       320       330       340       350       360
11a  CATTGCTAAACAAATATGATGATGTAGAAAATAGTGGTGGGTATGGTGGTAATCCTGGTC
45b  AAACCCCTGGCAGTTGTGTGTATTCCCCTTCTCCCAGTGGCTCTATTATTACTTCTG...

11a  AG
45b  ..
```

```
        1        10        20        30        40        50
11a  ..CCAAGGTTGTTGCCACGGATGCGTATGTTAAACGCACCAACATATTTTATCATGCCA.
45c  CAACCTGGTGACTGTC.CTCCTTTGGAACTTAAAAACACCATTATTGAGGATGGTGATAT

        60        70        80        90       100       110
11a  .GCAGTTCTAGACTCCTTGCTGTGGGACATCCATATTACTCTATCAAAAAGTTAACAAA
45c  GGTGGATACAGGTTATGGGGCAATGGATTTTAGTACATTGCAGGATACAAAGTGCGAGGT

       120       130       140       150       160       170
11a  ACAGTTGTACCAAAGGTG.TCTGGATATCAATATAGAGTGTTTAAGGTAGTGTTGCCAGA
45c  TCCATTAGACATTTGTCAATCCATCTGTAAATATCCAGATTATTTGCAAATGTCTGCTGA

        180       190       200       210       220       230
11a  TCCTAACAAG...TTTGCATTACCTGATTCATCTCTGTTTGACCCACTACACAGCGTTT
45c  TCCCTATGGGGATTCTATGTTTTTTTGCCTACGCCGTGAACAACTGTTTGCAAGACATTT

             240       250       260       270       280
11a  ..AGTATGGGC...GTGCACAGGGTTGGA.GGTAGGCAGGGGTCAACCTTT...AGGCGT
45c  TTGGAATAGGGCAGGTGTTATGGGTGACACAGTACCTACGGACCTATATATTAAAGGCAC

          290       300       310       320       330       340
11a  TGGTGTTAGTGGGCATCCATTGCTAAACAAATATGATGATGTAGAAAATAGTGGTGGGTA
45c  TAGCGCTAATATGCGTGAAACCCCTGGCAGTTGTGTGTATTCCCCTTCTCCCAGTGGCTC

          350       360
11a  TGGTGGTAATCCTGGTCAG
45c  TATTATTACTTCTG.....
```

```
                1        10        20        30        40        50
                .         .         .         .         .         .
11a   .......CCAAGGTTGTTGCCACGGATGCGTATGTTAAACGCACCAACATATTTTATCAT
52    CCTGTCTCTAAGGTTGTAAGCACTGATGAGTATGTGTCTCGCACAAGCATCTATTATTAT

             60        70        80        90       100       110
              .         .         .         .         .         .
11a   GCCAGCAGTTCTAGACTCCTTGCTGTGGGACATCCATATTACTCTATCAAAAAAGTTAAC
52    GCAGGCAGTTCTCGATTACTAACAGTAGGACATCCCTATTTTTCTATTAAAAACACCAGT

                      120        130       140       150
                        .          .         .         .
11a   A............AAACAGTTGTA...CCAAAGGTGTCTGGATATCAATATAGAGTGTTT
52    AGTGGTAATGGTAAAAAAGTTTTAGTTCCCAAGGTGTCTGGCCTGCAATACAGGGTATTT

      160       170       180       190       200       210
        .         .         .         .         .         .
11a   AAGGTAGTGTTGCCAGATCCTAACAAGTTTGCATTACCTGATTCATCTCTGTTTGACCCC
52    AGAATTAAATTGCCGGACCCTAATAAATTTGGTTTTCCAGATACATCTTTTTATAACCCA

      220       230       240       250       260       270
        .         .         .         .         .         .
11a   ACTACACAGCGTTTAGTATGGGCGTGCACAGGGTTGGAGGTAGGCAGGGGTCAACCTTTA
52    GAAACCCAAAGGTTGGTGTGGGCCTGTACAGGCTTGGAATTGGTAGGGGACAGCCTTTA

      280       290       300       310       320       330
        .         .         .         .         .         .
11a   GGCGTTGGTGTTAGTGGGCATCCATTGCTAAACAAATATGATGATGTAGAAAATAGTGGT
52    GGTGTGGGTATTAGTGGG..........................................

      340       350       360
        .         .         .
11a   GGGTATGGTGGTAATCCTGGTCAG
52    ........................
```

```
                    1          10        20        30        40
11a  ...............CCAAGGTTGTTGCCACGGATGCGTATGTTAAACGCACCAACATAT
58   CTCCTGTGCCTGTGTCTAAGGTTGTAAGCACTGATGAATATGTGTCACGCACAAGCATTT

       50        60        70        80        90        100
11a  TTTATCATGCCAGCAGTTCTAGACTCCTTGCTGTGGGACATCCATATTACTCTATCAAAA
58   ATTATTATGCTGGCAGTTCCCGACTTTTGGCTGTTGGCAATCCATATTTTTCCATCAAGA

              110       120       130       140       150
11a  .........AAGTTAACAAAACAGTTGTACCAAAGGTGTCTGGATATCAATATAGAGTGT
58   GTCCCAATAACAATAAAAAAGTATTAGTTCCCAAGGTATCAGGCTTACAGTATAGGGTCT

     160       170       180       190       200       210
11a  TTAAGGTAGTGTTGCCAGATCCTAACAAGTTTGCATTACCTGATTCATCTCTGTTTGACC
58   TTAGGGTGCGTTTACCTGATCCCAATAAATTTGGTTTTCCTGATACATCTTTTTATAACC

     220       230       240       250       260       270
11a  CCACTACACAGCGTTTAGTATGGGCGTGCACAGGGTTGGAGGTAGGCAGGGGTCAACCTT
58   CTGATACACAACGTTTGGTCTGGGCATGTGTAGGCCTTGAAATAGGTAGAGGACAGCCAT

     280       290       300       310       320       330
11a  TAGGCGTTGGTGTTAGTGGGCATCCATTGCTAAACAAATATGATGATGTAGAAAATAGTG
58   TGG.........................................................

     340       350       360
11a  GTGGGTATGGTGGTAATCCTGGTCAG
58   ..........................
```

```
      1        10        20        30        40        50        60
11b   CCTTTAGGCGTTGGTGTTAGTGGGCATCCATTGCTAAACAAATATGATGATGTAGAAAT
16    .............CTGTCCCAGTATCTAAGGTTGTAAGCACGGATGAATATGTTGCACGC

              70        80        90       100       110
11b   AGTGGTGGGTATGGTGGT..AATCCTGGTCAGGATAATAGGGTTAATGTAGGTATGGATT
16    ACAAACATATATTATCATGCAGGAACATCCAGACTACTTGCAGTTGGACATCCCTATTTT

     120       130       140       150       160       170
11b   ATAAACAAACCCAGCTATGTATGGTGGGCTGTGCTCCACCGTTAGGTGAACATTGGGGTA
16    CCTATTAAAAA.ACCTAACAATAACAAAATATTAGTTCCTAAAGTATCAGGATTACAATA

      180       190       200       210       220       230
11b   .AGGGTACACAATGTTCAAATACCTCTGTACAAAATGGTGACTGCCCCC..CGTTGGAAC
16    CAGGGTATTTAGAATACATTTACCTGACCCCAATAAGTTTGGTTTTCCTGACACCTCATT

        240       250       260       270       280       290
11b   TTATTACCAGTGTTATACAGGATGGGGACATGGTTGATACAGGCTTTGGTGCTATGAATT
16    TTATAATCCA.GATACACAGCGGCTGGTTTGGGCCTGTGTAGGTGTTGAGG...TAGGTC

        300       310       320
11b   TTGCAGACTTACAAACCAATAAATCGGATG
16    GTGGTCAGCCATTAG...............
```

```
                1        10        20        30          40          50
11b   ......CCTTTAGGCGTTGGTGTTAGTGGGCATCCATTGCTAAA...CAAATATGATGAT
18    CACTGGGCTAAAGGCACTGCTTGTAAATCGCGTCCTTTATCACAGGGCGATTGCCCCCCT

                60        70        80        90       100       110
11b   GTAGAAAATAGTGGTGGGTATGGTGGTAATCCTGGTCAGGATAATAGGGTTAATGTAGGT
18    TTAGAACTTAAAAACACAGTTTTGGAAGATGGTGATATGGTAGATACTGGATATGGTGCC

               120        130       140       150       160       170
11b   ATGGATTATAA.ACAAACCCAGCTATGTATGGTGGCTGTGCTCCACCGTTAGGTGAACA
18    ATGGACTTTAGTACATTGCAAGATACTAAATGTGAGGTACCATTGGATATTTG.TCAGTC

                180       190       200       210       220       230
11b   TTGGGGTAAGGGTACACAATGTTCAAATACCTCTGTACAAAATGGTGACTGCCCCCCGTT
18    TATTTGTAAATATCCTGATTATTTACAAATGTCTGCAGATCCTTATGGGGATTCCATGTT

                  240       250       260       270       280
11b   GGA..ACTTATTACCAGTGTTATACAGGATGGGGACATGGTTGATACAGGCTTTGGTGCT
18    TTTTTGCTTACGGCGTGAGCAGCTTTTTGCTAGG.CATTTTTGGAATAGAGCA.GGTACT

    290       300       310        320
11b   ATGAATTTTGCAGACTTACAAACCA.ATAAATCGGATG......................
18    ATGGGTGACACTGTGCCTCAATCCTTATATATTAAAGGCACAGGTATGCGTGCTTCACCT

11b   ............................................................
18    GGCAGCTGTGTGTATTCTCCCTCTCCAAGTGGCTCTATTGTTACCTCTGACTCCCAGTTG
```

```
        1        10        20        30        40        50        60
11b     CCTTTAGGCGTTGGTGTTAGTGGGCATCCATTGCTAAACAAATATGATGATGTAGAAAAT
31      ...CTGTCCCAGTGTCTAAAGTTGTAAGCACGGATGAATATGTAACACGAACCAACATAT

                 70        80        90       100       110       120
11b     AGTGGTGGGTATGGTGGTAATCCTGGTCAGGATAATAGGGTTAATGTAGGTATGGATTAT
31      ATTATCACGCA....GGCAGTGCTAGGCTGCTTACAGTAGGCCATCCATAT.TATTCCAT

               130       140       150       160       170
11b     AAACAAACCCAGCTATGTATGGTGGGCTGTGCTCCACCGTTAGGTGAACATTGGGGTA.A
31      ACCTAAATCTGACAATCCTAAAAAAATAGTTGTAC.CAAAGGTGTCAGGATTACAATATA

      180       190       200       210       220       230
11b     GGGTACACAATGTTCAAATACCTCTGTACAAAATGGTGACTGCCCCCCGTTGGAACTTAT
31      GGGTATTTAGGGTTCGTTTACCAG.ATCCAAACAAATTTGGATTTCCTGATACATCTTTT

      240       250       260       270       280       290
11b     TACCAGTGTTATACAGGATGGGGACATGGTTGATACAG.GCTTTGGTGCTATGAATTTTG
31      TATAATCCTGAAACTCAACGCTTAGTTTGGGCCTGTGTTGGTTTAGAGGTAGGTCGCGGG

        300       310       320
11b     CAGACTTACAAACCAATAAATCGGATG
31      CAGCCATTAGGTGTAGGTATTAGTG..
```

```
                            1        10        20        30
11b  .....................CCTTTAGGCGTTGGTGTTAGTGGGCATCCATTGCTAAAC
33   CTTGAAATAGGTAGAGGGCAGCCATTAGGCGTTGGCATAAGTGGTCATCCTTTATTAAAC

     40        50        60        70        80        90
11b  AAATATGATGATGTAGAAAATAGTGGTGGGTATGGTGGTAATCCTGGTCAGGATAATAGG
33   AAATTTGATGACACTGAAACCAGTAACAAGTATCCTGGACAACCGGGTGCTGATAATAGG

    100       110       120       130       140       150
11b  GTTAATGTAGGTATGGATTATAAACAAACCCAGCTATGTATGGTGGCTGTGCTCCACCG
33   GAATGTTTATCCATGGATTATAAACAAACACAGTTATGTTTACTTGGATGTAAGCCTCCA

    160       170       180       190       200       210
11b  TTAGGTGAACATTGGGGTAAGGGTACACAATGTTCAAATACCTCTGTACAAAATGGTGAC
33   ACAGGGGAACATTGGGGTAAAGGTGTTGCTTGTACTAATG...CAGCACCTGCCAATGAT

    220       230       240       250       260       270
11b  TGCCCCCGTTGGAACTTATTACCAGTGTTATACAGGATGGGGACATGGTTGATACAGGC
33   TGTCCACCTTTAGAACTTATAAATACTATTATTGAGG.......................

    280       290       300       310       320
11b  TTTGGTGCTATGAATTTTGCAGACTTACAAACCAATAAATCGGATG
33   ..............................................
```

```
              1        10        20        30        40        50
11b   .......CCTTTAGGCGTTGGTGTTAGT...GGGCATCCATTGCTAAACAAATATGATGA
45a   CCCTTCTCCCAGTGGCTCTATTATTACTTCTGATTCTCAATTATTTAATAAGCCATATTG

                60        70        80        90       100       110
11b   TGTAGAAAATAGTGGTGGGTATGGTGGTAATCCTGGTCAGGATAATAGGGTTAATGTAGG
45a   GTTACATAAGGCCCAGGGCCATAACAATGGTATTTGTTGGCATAATCAGTTGTTTGTTAC

                120       130       140       150       160
11b   TATGG.....ATTATAAACAAACCCAGCTATGTATGGTGGGCTGTGCTCCACCGTTAGGT
45a   TGTAGTGGACACTACCCGCAGTACTAATTTAACATTATGTGCCTCTACACAAAATTCTGT

        170       180       190       200       210       220
11b   GAACATTGGGGTAAGGGTACACAATGTTCAAATACCTCTGTACAAATGGTGACTGCCCC
45a   GCCAAGTACA.TATGACCCTACTAAGTTTAAGCAGTATAGTA.GACATGTGGAGGAATAT

        230       240        250           260       270
11b   CCGTTGGAACTTATTAC.CAGTGTTATAC....AGGATGGGGACATGGTTGATA.CAGGC
45a   GATTTACAGTTTATTTTTCAGTTGTGCACTATTACTTTAACTGCAGAGGTTATGTCATAT

     280       290       300       310       320
11b   TTTGGTGCTATGAATTTTGCAGACTTACAAACCAATAAATCGGATG..............
45a   ATCCATAGTATGAATAGTAGTATATTAGAAAATTGGAATTTTGGTGTCCCTCCACCACCT

11b   .............
45a   ACTACAAGTTTGG
```

```
      1       10        20        30        40        50
6b    .CAGCCATTAGGTGTG.GGTGTAAGTGGACATCCTTTCCTAAATAAATATGATGATGTTG
45b   CAGGATACAAAGTGCGAGGTTCCATTAGACAT..TTGTCAATCCATCTGTAAATATCCAG

      60        70        80        90        100             110
6b    AAAATTCAGGGAGTGGTGGTAACCCTGGACAGGATAACAGGGTTA.......ATGTTGGT
45b   ATTATTTGCAAATGTCTGCTGATCCCTATGGGGATTCTATGTTTTTTTGCCTACGCCGTG

               120        130       140       150       160       170
6b    ATGGATTATAAACAA.ACACAATTATGCATGGTTGGATGTGCCCCCCCTTTGGGCGAGCA
45b   AACAACTGTTTGCAAGACATTTTTGGAATAGGGCAGGTGTTATGGGTGACACAGTACCTA

                180       190              200       210       220
6b    TTGGGGTAAAGGTAAACAGT....GTACTAATACACCTGTACAGGCTGGTGACTGCCCGC
45b   CGGACCTATATATTAAAGGCACTAGCGCTAATATGCGTGAAACCCCTGGCAGTTGTGTGT

        230       240
6b    CCTTAGAACTTATTACCAG.................
45b   ATTCCCCTTCTCCCAGTGGCTCTATTATTACTTCTG
```

```
                       1          10        20        30        40
11b   ...........CCTTTAGGCGTTGGTGTTAGTGGGCATCCATTGCTAAACA.AATATGAT
45c   CAACCTGGTGACTGTCCTCCTTTGGAACTTAAAAACA.CCATTATTGAGGATGGTGATAT

      50        60        70        80        90        100
11b   GATGTAGA.AAATAGTGGTGGGTATGGTGGTAATCCTGGTCAGGATAATAGGGTTAATGT
45c   GGTGGATACAGGTTATGGGGCAATGGATTTTAGTACATTGCAGGATACAAAGTGCGAGGT

      110       120       130       140       150       160
11b   AGGTATGGATTATAAACAAACCCAGCTATGTATGGT.GGGCTGTGCTCCACCGTTAGGTG
45c   TC.CATTAGACATTTGTCAATCCATCTGTAAATATCCAGATTATTTGCAAATGTCTGCTG

       170       180       190       200       210       220
11b   AACATTGGGGTAAGGGTACACAATGTTCAAATACCTCTGTACAAAATGGTGACTGCCCCC
45c   ATCCCTATGGGGATTCTATGTT.TTTTTGCCTACGCCGTGAACAACTGTTTGCAAGACAT

       230              240       250       260       270
11b   CGTTGGAACT.......TATTACCAGTGTTATACAGGATGGGGACATGGTTGATACAGGC
45c   TTTTGGAATAGGGCAGGTGTTATGGGTGACACAGTACCTACGGACCTATATATTAAAGGC

    280       290       300       310       320
11b   TTTGGTGCTATGAATTTTGCAGACTTACAAACCAATAAATCGGATG..............
45c   ACTAGCGCTAATATGCGTGAAACCCCTGGCAGTTGTGTGTATTCCCCTTCTCCCAGTGGC

11b   ................
45c   TCTATTATTACTTCTG
```

```
               1        10        20        30        40        50
11b   ......CCTTTAGGCGTTGGTGTTAGTGGGCATCCATTGC...TAAACAAATATGATGAT
52    CCTGTCTCTAAGGTTGTAAGCACTGATGAGTATGTGTCTCGCACAAGCATCTATTATTAT

                60        70        80        90       100
11b   GTAGAAAATAGTGG..TGGGTATGGTGGTA.ATCCTGGTCAGGATAATAGGGTTAATGTA
52    GCAGGCAGTTCTCGATTACTAACAGTAGGACATCCCTATTTTTCTATTAAAACACCAGT

     110       120       130       140       150       160
11b   GGTATGGATTATAAACAAACCCAGCTATGTATGGTGGGCTGTGCTCCACCGTTAGGTG..
52    AGTGGTAATGGTAAAAAAGTTTAGTTCCCAAGGTGT.CTGGCCTGCAATACAGGGTATT

        170       180       190       200       210       220
11b   .AACATTGGGGTAAGGGTACACAATG..TTCAAATACCTCTGTACAAAATGGTGACTGCC
52    TAGAATTAAATTGCCGGACCCTAATAAATTTGGTTTTCCAGATACATCTTTTTATAACCC

          230       240       250       260       270       280
11b   CCCCGTTGGAACTTATTACCAGTGTTATACAGGATGGGACATGGTTGATACAGGCTTTG
52    AGAAACCCAAAGGTTGGTGTGGGCCTGTACAGGCTTGGAAATTGGTAGGGGACAGCCTTT

          290       300       310       320
11b   GTGCTATGAATTTTGCAGACTTACAAACCAATAAATCGGATG
52    AGGTGTGGGTATTAGTGGG........................
```

```
              1        10        20        30        40        50        60
11b    CCTTTAGGCGTTGGTGTTAGTGGGCATCCATTGCTAAACAAATATGATGATGTAGAAAAT
58     CTCCTGTGCCTGTGTCTAAGGTTGTAAGCACTGATGAATATGTGTCACGC....ACAAGC

                 70        80        90       100       110       120
11b    AGTGGTGGGTATGGTGGTAATCCTGGTCAGGATAATAGGGTTAATGTAGGTATGGATTAT
58     ATTTATTATTATGCTGGCAGTTCCCGACTTTTGGCTGTTGGCAATCCATATTTTT.CCAT

                130       140       150       160       170
11b    AAACAAACCCAGCTATGTATGGTGGGCTGTGCTCCACCGTTAGGTGAACATTGGGGTA.A
58     CAAGAGTCCCAATAACA.ATAAAAAAGTATTAGTTCCCAAGGTATCAGGCTTACAGTATA

     180       190       200       210       220       230
11b    GGGTACACAATGTTCAAATACCTCTGTACAAAATGGTGACTGCCCCCCGTTGGAACTTAT
58     GGGTCTTTAGGGTGCGTTTACCTGATCCCAATAAATTTGGT.TTTCCTGATACATCTTTT

     240       250       260       270       280       290
11b    TACCAGTGTTATACAGGATGG..GGACATGGTTGATACAGGCTTTGGTGCTATGAATTTT
58     TATAACCCTGATACACAACGTTTGGTCTGGGCATGTGTAGGCCTTGAAA.TAGGTAGAGG

       300       310       320
11b    GCAGACTTACAAACCAATAAATCGGATG
58     ACAGCCATTGG.................
```

```
16    ............................................................
18    CACTGGGCTAAAGGCACTGCTTGTAAATCGCGTCCTTTATCACAGGGCGATTGCCCCCCT

                                        1        10        20
16    ..................................CTGTCCCAGTATCTAAGGTTGTAAGC
18    TTAGAACTTAAAAACACAGTTTTGGAAGATGGTGATATGGTAGATACTGGATATGGTGCC

         30        40        50        60        70        80
16    ACGGA..TGAATATGTTGCACGCA..CAAACATATATTATCATGCAGGAACATCCAGACT
18    ATGGACTTTAGTACATTGCAAGATACTAAATGTGAGGTACCATTGGATATTTGTCAGTCT

           90        100       110          120       130
16    ACTTGCAGTTGGACATCCCTATTTTCCTAT...TAAAAAACCTAACAATAACAAAATATT
18    ATTTGTAAATATCC.TGATTATTTACAAATGTCTGCAGATCCTTATGGGGATTCCATGTT

     140       150       160       170       180       190
16    AGTT..CCTAAAGTATCAGGATTACAATACAGGGTATTTAGAATACATTTACCTGACCC
18    TTTTGCTTACGGCGTGAGCAGCTTTTTGCTAGGCATTTTTGGAATAGAGCAGGTACTAT

       200       210       220       230       240       250
16    AATAAGTTTGGTTTTCCTGACACCTCATTTTATAATC.CAGATACACAGCGGCTGGTTTG
18    GGGTGACACTGTGCCTCAATCCTTATATATTAAAGGCACAGGTATGCGTGCTTCACCTGG

        260       270       280        290
16    GGCCTGTGTAGGTGTTGAGGTAGGTCGTGG.TCAGCCATTAG................
18    CAGCTGTGTGTATTCTCCCTCTCCAAGTGGCTCTATTGTTACCTCTGACTCCCAGTTG
```

```
       1        10        20        30        40        50        60
16     CTGTCCCAGTATCTAAGGTTGTAAGCACGGATGAATATGTTGCACGCACAAACATATATT
31     CTGTCCCAGTGTCTAAAGTTGTAAGCACGGATGAATATGTAACACGAACCAACATATATT

                70        80        90       100       110       120
16     ATCATGCAGGAACATCCAGACTACTTGCAGTTGGACATCCCTATTTTCCTATTAAAAAAC
31     ATCACGCAGGCAGTGCTAGGCTGCTTACAGTAGGCCATCCATATTATTCCATACCTAAAT

                 130       140       150       160       170
16     CTAACAAT...AACAAAATATTAGTTCCTAAAGTATCAGGATTACAATACAGGGTATTTA
31     CTGACAATCCTAAAAAAATAGTTGTACCAAAGGTGTCAGGATTACAATATAGGGTATTTA

       180       190       200       210       220       230
16     GAATACATTTACCTGACCCCAATAAGTTTGGTTTTCCTGACACCTCATTTTATAATCCAG
31     GGGTTCGTTTACCAGATCCAAACAAATTTGGATTTCCTGATACATCTTTTTATAATCCTG

       240       250       260       270       280       290
16     ATACACAGCGGCTGGTTTGGGCCTGTGTAGGTGTTGAGGTAGGTCGTGGTCAGCCATTAG
31     AAACTCAACGCTTAGTTTGGGCCTGTGTTGGTTTAGAGGTAGGTCGCGGGCAGCCATTAG

16     ...............
31     GTGTAGGTATTAGTG
```

```
        1        10        20        30        40        50        60
16  CTGTCCCAGTATCTAAGGTTGTAAGCACGGATGAATATGTTGCACGCACAAACATATATT
33  ...........CTTGAAATAGGTAGAGGGCAGCCATTAGGCGTTGGCATAAGTGGTCATC

                 70        80        90       100       110       120
16  ATCATGCAGGAACATCCAGACTACTTGCAGTTGGACATCCCTATTTTCCTATTAAAAAAC
33  CTTTATTAAACAAATTTGATGACACTGAAACCAGTAACAAGTATCCTGGACAACCGGGTG

                130       140       150       160       170       180
16  CTAACAATAACAAAATATTAGTTCCTAAAGTATCAGGATTACAATACAGGGTATTTAGAA
33  CTGATAATAG.GGAATGTTTATCCATGGATTATAAACAAACACAGTTATGTTTACTTGGA

                190       200       210       220       230       240
16  TACATTTACCTGACCCCAATAAGTTTGGTTTTCCTGACACCTCATTTTATAATCCAGATA
33  TGTAAGCCTCCAACAGGGGAACATTGGGGTAA..AGGTGTTGCTTGTACTAATGCAG.CA

                250       260       270       280       290
16  CACAGCGGCTGGTTTGGGCCTGTGTAGGTGTTGAGGTAGGTCGTGGTCAGCCATTAG
33  CCTGCCAATGATTGTCCACCTTTAGAACTTATAAATACTATTATTGAGG........
```

```
                                                    1
16   ....................................................CTGTCCCA
45a  CCCTTCTCCCAGTGGCTCTATTATTACTTCTGATTCTCAATTATTTAATAAGCCATATTG

      10        20        30        40        50        60
16   GT.ATCTAAGGTTGTAAGCACGGATGAATATGTT.GCACGCACAAACATATATTATCATG
45a  GTTACATAAGGCCCAGGGCCATAACAATGGTATTTGTTGGCATAATCAGTTGTT.TGTTA

        70        80        90           100       110          120
16   CAGGAACATCCAGACTACTTGCAGTTGGA...CATCCTATTTTCCTAT.TAAAAAACCT
45a  CTGTAGTGGACA..CTACCCGCAGTACTAATTTAACATTATGTGCCTCTACACAAAATTC

           130       140       150       160       170       180
16   AACAATAACAAAATATTAGTTCCTAAAGTATCAGGATTACAATACAGGGTATTTAGAATA
45a  TGTGCCAAGTACATATGACCCTACTAAGTTTAAGCAGTATAGTAGACATGTGGAGGAATA

               190       200       210       220       230
16   C.ATTTAC...CTGACCCCAATAAGTTTGGTTTTCCTGACACCTCATTTTATAATCCAGA
45a  TGATTTACAGTTTATTTTTCAGTTGTGCACTATTACTTAACTGCAGAGGTTATGTCATA

     240       250       260       270       280       290
16   TACACAGCGGCTGGTTTGGGCCTGTGTAGGTGTTGAGGTAGGTCGTGGTCAGCCATTAG.
45a  TATCCATAGTATGAATAGTAGTATATTAGAAAATTGGAATTTTGGTGTCCCTCCACCACC

16   ..............
45a  TACTACAAGTTTGG
```

```
             1        10        20        30        40        50
16     .CTGTCCCAGTATCTAAGGTTGTAAGCACGGATGAATATGTTGCACGCACAAACATATAT
45b    CAGGATACAAAGTGCGAGGTTCCATTAGACATTTGTCAATCCATCTGTAAATATCCAGAT

       60        70        80        90        100       110
16     TATCATGCAGGAACATCCAGACTACTTGCAGTTGGACATCCCTATTTTCCTATTAAAAAA
45b    TATT.TGCAAATGTCTGCTGATCCCTATGGGGATTCTATGTTTTTTTGCCTACGCCGTGA

      120       130       140       150       160       170
16     CCTAACAATAACAAAATATTAGTTCCTAAAGTATCAGGATTACAAT..ACAGGGTATTTA
45b    ACAACTGTTTGCAAGACATTT.TTGGAATAGGGCAGGTGTTATGGGTGACACAGTACCTA

        180       190       200       210       220       230
16     GAATACATTTACCTGACCCCAATAAGTTTGGTTTTCCTGACACCTCATTTTATAATCCAG
45b    CGGACCTATATATTAAAGGCACTAGCGCTAATATGCGTGAAACCCCTGGCAGTTGTGTGT

        240       250       260       270       280       290
16     ATACACAGCGGCTGGTTTGGGCCTGTGTAGGTGTTGAGGTAGGTCGTGGTCAGCCATTAG
45b    ATTCCCCTTCTCCCAGTGGCTCTATTATTACTTCTG........................
```

```
                                        1         10        20
16     ................................CTGTCCCAGTATCTAAGGTTGTAAGCAC
45c    CAACCTGGTGACTGTCCTCCTTTGGAACTTAAAAACACCATTATTGAGGATGGTGA.TAT

       30        40        50        60        70        80
16     GGATGAATATGTTGCACGCACAAACATATATTATCATGCAGGAACATCCAGACTACTTG.
45c    GG.TGGATACAGGTTATGGGGCAATGGATTTTAGTACATTGCAGGATACAAAGTGCGAGG

          90       100       110        120       130          140
16     ..CAGTTGGACATCCCTATTTTCCTAT.TAAAAAACCTAACAATAACAAAATAT....TA
45c    TTCCATTAGACATTTGTCAATCCATCTGTAAATATCCAGATTATTTGCAAATGTCTGCTG

              150          160       170       180       190
16     GTTCCTAAAG...TATCAGGATTACAATACAGGGTATTTAGAATACATTTACCTGACCCC
45c    ATCCCTATGGGGATTCTATGTTTTTTGCCTACGCCGTGAACAACTGTTTGCAAGACATT

            200       210          220       230       240
16     .....AATAAGTTTGGTTTTCC...TGACACCTCATTTTATAATCCAGATAC.ACAGCGG
45c    TTTGGAATAGGGCAGGTGTTATGGGTGACACAGTACCTACGGACCTATATATTAAAGGCA

     250       260       270       280       290
16     CTGGTTTGGGCCTGTGTAGGTGTTGAGGTAGGTCGTGGTCAGCCATTAG...........
45c    CTAGCGCTAATATGCGTGAAACCCCTGGCAGTTGTGTGTATTCCCCTTCTCCCAGTGGCT

16     ...............
45c    CTATTATTACTTCTG
```

```
      1        10        20        30        40        50        60
16    CTGTCCCAGTATCTAAGGTTGTAAGCACGGATGAATATGTTGCACGCACAAACATATATT
52    .....CCTGTCTCTAAGGTTGTAAGCACTGATGAGTATGTGTCTCGCACAAGCATCTATT

               70        80        90       100       110       120
16    ATCATGCAGGAACATCCAGACTACTTGCAGTTGGACATCCCTATTTTCCTATTAAAAAAC
52    ATTATGCAGGCAGTTCTCGATTACTAACAGTAGGACATCCCTATTTTTCTATTAAAAACA

                    130       140       150       160       170
16    C.........TAACAATAACAAAATATTAGTTCCTAAAGTATCAGGATTACAATACAGGG
52    CCAGTAGTGGTAATGGTAAAAAAGTTTTAGTTCCCAAGGTGTCTGGCCTGCAATACAGGG

            180       190       200       210       220       230
16    TATTTAGAATACATTTACCTGACCCCAATAAGTTTGGTTTTCCTGACACCTCATTTTATA
52    TATTTAGAATTAAATTGCCGGACCCTAATAAATTTGGTTTTCCAGATACATCTTTTTATA

            240       250       260       270       280       290
16    ATCCAGATACACAGCGGCTGGTTTGGGCCTGTGTAGGTGTTGAGGTAGGTCGTGGTCAGC
52    ACCCAGAAACCCAAAGGTTGGTGTGGGCCTGTACAGGCTTGGAAATTGGTAGGGGACAGC

16    CATTAG.................
52    CTTTAGGTGTGGGTATTAGTGGG
```

```
                1        10        20        30        40        50
16    ...CTGTCCCAGTATCTAAGGTTGTAAGCACGGATGAATATGTTGCACGCACAAACATAT
58    CTCCTGTGCCTGTGTCTAAGGTTGTAAGCACTGATGAATATGTGTCACGCACAAGCATTT

         60        70        80        90       100       110
16    ATTATCATGCAGGAACATCCAGACTACTTGCAGTTGGACATCCCTATTTTCCTATTAAAA
58    ATTATTATGCTGGCAGTTCCCGACTTTTGGCTGTTGGCAATCCATATTTTTCCATCAAGA

      120            130       140       150       160       170
16    AACC...TAACAATAACAAAATATTAGTTCCTAAAGTATCAGGATTACAATACAGGGTAT
58    GTCCCAATAACAATAAAAAAGTATTAGTTCCCAAGGTATCAGGCTTACAGTATAGGGTCT

         180       190       200       210       220       230
16    TTAGAATACATTTACCTGACCCCAATAAGTTTGGTTTTCCTGACACCTCATTTTATAATC
58    TTAGGGTGCGTTTACCTGATCCCAATAAATTTGGTTTTCCTGATACATCTTTTTATAACC

         240       250       260       270       280       290
16    CAGATACACAGCGGCTGGTTTGGGCCTGTGTAGGTGTTGAGGTAGGTCGTGGTCAGCCAT
58    CTGATACACAACGTTTGGTCTGGGCATGTGTAGGCCTTGAAATAGGTAGAGGACAGCCAT

16    TAG
58    TGG
```

```
     1        10        20        30        40        50        60
18   CACTGGGCTAAAGGCACTGCTTGTAAATCGCGTCCTTTATCACAGGGCGATTGCCCCCCT
31   .......................................................CTGTC

               70        80        90       100       110       120
18   TTAGAACTTAAAAACACAGTTTTGGAAGATGGTGATATGGTAGATACTGGATATGGTGCC
31   CCAGTGTCTAAAGTTGTAAGCACGGATGAATATGTAACACGAACCAACATATATTATCAC

              130       140       150       160       170
18   ATGGACTTTAGTACATTGCAAGATACTAAATGTGAGGTACCATTGGATATTTGTCAGTC.
31   GCAGGCAGTGCTAGGCTGCTT.ACAGTAGGCCATCCATATTATTCCATACCTAAATCTGA

   180       190       200       210       220       230
18   TATTTGTAAATATCCTGATTATTTACAAATGTCTGCAGATCCTTATGGGGATTCCATGTT
31   CAATCCTAAAAAAATAGTTGTACCAAAGGTGTCAGGATTACAATATAGGGTATTTAGGGT

   240       250       260          270       280       290
18   TTTTTGCTTACGGCGTGAGCAGCTT...TTTGCTAGGCATTTTTGGAATAGAGCAGGTAC
31   TCGTTTACCAGATCCAAACAAATTTGGATTTCCTGATACATCTTTTTATAATCCTGAAAC

      300       310       320       330       340       350
18   TATGGGTGACACTGTGCCTCAATCCTTATATATTAAAGGCACAGGTATGCGTGCTTCACC
31   T.....CAACGCTTAGTTTGGGCCTGTGTTGGTTTAGAG.GTAGGT.CGCGGGC...AGC

      360       370       380       390       400       410
18   TGGCAGCTGTGTGTATTCTCCCTCTCCAAGTGGCTCTATTGTTACCTCTGACTCCCAGTT
31   CATTAGGTGTAGGTATTAGTG.......................................

18   G
31   .
```

```
         1         10        20        30        40        50        60
18       CACTGGGCTAAAGGCACTGCTTGTAAATCGCGTCCTTTATCACAGGGCGATTGCCCCCCT
33       ............................................................

                   70        80        90        100       110       120
18       TTAGAACTTAAAAACACAGTTTTGGAAGATGGTGATATGGTAGATACTGGATATGGTGCC
33       ......................CTTGAAATAGGTAGAGGGCAGCCATTAGGCGT..TGGC

                  130       140       150       160       170       180
18       ATGGACTTTAGTACATTGCAAGATACTAAATGTGAGGTACCATTGGATATTTGTCAGTCT
33       ATAAGTGGTCATCCTTTATTAAA...CAAATTTGATGACACTGAAACCAGTAACAAGTAT

                  190       200       210       220       230       240
18       ATTTGTAAATATCCTGATTATTTACAAATGTCTGCAGATCCTTATGGGGATTCCATGTTT
33       CCTGGACAACCGGGTGCTGATAATAGGGAATGTTTATCCATGGATTATAAACAAACACAG

                  250       260       270       280       290       300
18       TTTTGCTTACGGCGTGAGCAGCTTTTTGCTAGGCATTTTTGGAATAGAGCAGGTACTATG
33       TTATGTTTACTTGGATGTAAGCCTCCAACAGGGAACATTGGGGTAAAGGTGTTGCTTGT

                  310         320       330       340       350
18       GGTGACACTGTGCCT..CAATCCTTATATATTAAAGGCACAGGTATGCGTGCTTCACCTG
33       ACTAATGCAGCACCTGCCAATGATTGTCCACCTTTAGAACTTATAAATACTATTATTGAG

        360       370       380       390       400       410
18       GCAGCTGTGTGTATTCTCCCTCTCCAAGTGGCTCTATTGTTACCTCTGACTCCCAGTTG
33       G..........................................................
```

```
        1        10        20        30        40        50
18      .CACTGGGCTAAAGGCACTGCTTGTAAATCGCGTCCTTTATCACA....GGGCGATTGCC
45a     CCCTTCTCCCAGTGGCTCTATTATTACTTCTGATTCTCAATTATTTAATAAGCCATATTG

           60        70        80        90       100       110
18      CCCCTTTAGAACTTAAAAACACAGTTTTGGAAGATGGTGATATGGTAGATACTGGATATG
45a     GTTACATAAGGCCCAGGGCCATAACAATGGTATTTGTTGGCATAATCAGTTGTTTGT.TA

          120       130       140       150       160       170
18      GTGCCATGGACTTTAGTACATTGCAAGATACTAAATGTGAGGTACCATTGGATATTTGTC
45a     CTGTAGTGGACACTA.CCCGCAGTACTA.ATTTAACATTATGTGCCTCTACACAAAATTC

           180       190       200       210       220       230
18      AGT.CTATTTGTAAATATCCTGATTATTTACAAATGTCTGCAGATCCTTATGGGGATTCC
45a     TGTGCCAAGTACATATGACCCTACTAAGTTTAAGCAGTATAGTAGACATGTGGAGGAAT.

           240       250       260       270       280       290
18      ATGTTTTTTTGCTTACGGCGTGAGCAGCTTTTTGCTAGGCATTTTTGGAATAGAGCAGGT
45a     ATGATTTACAGTTTAT....TTTTCAGTTGTGCACTAT.TACTTTAACTGCAGAGGTTAT

           300       310       320       330       340       350
18      ACTATGGGTGACACTGTGCCTCAATCCTTATATATTAAAGGCACAGGTATGCGTGCTTCA
45a     GTCATATATC.CATAGTA..TGAATAGTAGTATATTAGAAAATTGGAATTTTGGTGTCCC

           360       370       380       390       400       410
18      CCTGGCAGCTGTGTGTATTCTCCCTCTCCAAGTGGCTCTATTGTTACCTCTGACTCCCAG
45a     TCCACCACCTACTACAAGTTTGG.....................................

18      TTG
45a     ...
```

```
       1        10        20        30        40        50        60
18     CACTGGGCTAAAGGCACTGCTTGTAAATCGCGTCCTTTATCACAGGGCGATTGCCCCCCT
45b    ............................................................

               70        80        90       100       110       120
18     TTAGAACTTAAAAACACAGTTTTGGAAGATGGTGATATGGTAGATACTGGATATGGTGCC
45b    ............................................................

              130       140       150       160       170       180
18     ATGGACTTTAGTACATTGCAAGATACTAAATGTGAGGTACCATTGGATATTTGTCAGTCT
45b    ..................CAGGATACAAAGTGCGAGGTTCCATTAGACATTTGTCAATCC

              190       200       210       220       230       240
18     ATTTGTAAATATCCTGATTATTTACAAATGTCTGCAGATCCTTATGGGGATTCCATGTTT
45b    ATCTGTAAATATCCAGATTATTTGCAAATGTCTGCTGATCCCTATGGGGATTCTATGTTT

              250       260       270       280       290       300
18     TTTTGCTTACGGCGTGAGCAGCTTTTTGCTAGGCATTTTTGGAATAGAGCAGGTACTATG
45b    TTTTGCCTACGCCGTGAACAACTGTTTGCAAGACATTTTTGGAATAGGGCAGGTGTTATG

              310       320       330       340             350
18     GGTGACACTGTGCCTCAATCCTTATATATTAAAGGCACAGG......TATGCGTGCTTCA
45b    GGTGACACAGTACCTACGGACCTATATATTAAAGGCACTAGCGCTAATATGCGTGAAACC

          360       370       380       390       400       410
18     CCTGGCAGCTGTGTGTATTCTCCCTCTCCAAGTGGCTCTATTGTTACCTCTGACTCCCAG
45b    CCTGGCAGTTGTGTGTATTCCCCTTCTCCCAGTGGCTCTATTATTACTTCTG........

18     TTG
45b    ...
```

```
       1        10        20        30        40        50        60
18     CACTGGGCTAAAGGCACTGCTTGTAAATCGCGTCCTTTATCACAGGGCGATTGCCCCCCT
45c    .......................................CAACCTGGTGACTGTCCTCCT

                70        80        90       100       110       120
18     TTAGAACTTAAAAACACAGTTTTGGAAGATGGTGATATGGTAGATACTGGATATGGTGCC
45c    TTGGAACTTAAAAACACCATTATTGAGGATGGTGATATGGTGGATACAGGTTATGGGGCA

               130       140       150       160       170       180
18     ATGGACTTTAGTACATTGCAAGATACTAAATGTGAGGTACCATTGGATATTTGTCAGTCT
45c    ATGGATTTTAGTACATTGCAGGATACAAAGTGCGAGGTTCCATTAGACATTTGTCAATCC

               190       200       210       220       230       240
18     ATTTGTAAATATCCTGATTATTTACAAATGTCTGCAGATCCTTATGGGGATTCCATGTTT
45c    ATCTGTAAATATCCAGATTATTTGCAAATGTCTGCTGATCCCTATGGGGATTCTATGTTT

               250       260       270       280       290       300
18     TTTTGCTTACGGCGTGAGCAGCTTTTTGCTAGGCATTTTTGGAATAGAGCAGGTACTATG
45c    TTTTGCCTACGCCGTGAACAACTGTTTGCAAGACATTTTTGGAATAGGGCAGGTGTTATG

               310       320       330       340          350
18     GGTGACACTGTGCCTCAATCCTTATATATTAAAGGCACAGG......TATGCGTGCTTCA
45c    GGTGACACAGTACCTACGGACCTATATATTAAAGGCACTAGCGCTAATATGCGTGAAACC

           360       370       380       390       400       410
18     CCTGGCAGCTGTGTGTATTCTCCCTCTCCAAGTGGCTCTATTGTTACCTCTGACTCCCAG
45c    CCTGGCAGTTGTGTGTATTCCCCTTCTCCCAGTGGCTCTATTATTACTTCTG........

18     TTG
45c    ...
```

```
         1        10        20        30        40        50        60
18       CACTGGGCTAAAGGCACTGCTTGTAAATCGCGTCCTTTATCACAGGGCGATTGCCCCCCT
52       ............................................................

                  70        80        90       100       110       120
18       TTAGAACTTAAAAACACAGTTTTGGAAGATGGTGATATGGTAGATACTGGATATGGTGCC
52       ...............CCTGTCTCTAAGGTTGTAAGCACTGATGAGTATGTGTCTCGCACA

                 130       140       150       160       170       180
18       ATGGACTTTAGTACATTGCAAGATACTAAATGTGAGGTACCATTGGATATTTGTCAGTCT
52       A..GCATCTATTATTATGCAGGCAGTTCTCGATTACTAACAGTAGGACATCC..CTATTT

                 190       200       210       220       230       240
18       ATTTGTAAATATCCTGATTATTTACAAATGTCTGCAGATCCTTATGGGGATTCCATGTTT
52       TTCTATTAAAAACACCAGTAGTGGTAATGGTAAAAAAGTTTT.....AGTTCCCAAGGTG

                 250       260       270         280       290
18       TTTTGCTTACGGCGTGAGCAGCTTTTTGCTAGGCATTT...TTGGAATAGAGCAGGTACT
52       TCTGGCCTGCAATACAGGGTATTTAGAATTAAATTGCCGGACCCTAATAAATTTGGTTTT

         300       310       320       330       340       350
18       ATGGGTGACACTGTGCCTCAATCCTTATATATTAAAGGCACAGGTATGCGTGCTTCACCT
52       CCAGAT.ACATCTTTTTATAACCCAGAAAC.CCAAAGGTTGGTGTGGCCTGTACAGGCT

         360       370       380       390       400       410
18       GGCAGCTGTGTGTATTCTCCCTCTCCAAGTGGCTCTATTGTTACCTCTGACTCCCAGTTG
52       TGGAAATTGGTA.GGGGACAGCCTTTAGGTGTGGGTATTAGTGGG...............
```

```
       1        10        20        30        40        50        60
18     CACTGGGCTAAAGGCACTGCTTGTAAATCGCGTCCTTTATCACAGGGCGATTGCCCCCCT
58     ............................................................

               70        80        90       100       110       120
18     TTAGAACTTAAAAACACAGTTTTGGAAGATGGTGATATGGTAGATACTGGATATGGTGCC
58     ................CTCCTGTGCCTGTGTCTAAGGTTGTAAGCACTGATGAATATGTG

              130       140       150       160       170       180
18     ATGGACTTTAGTACATTGCAAGATACTAAATGTGAGGTACCATTGGATATTTGTCAGTCT
58     TCACGCACAAGCATTTATTATTATGCTGGCAGTTCCCGACTTTTGGCTGTT.GGCAATCC

              190       200       210       220       230       240
18     ATTTGTAAATATCCTGATTATTTACAAATGTCTGCAGATCCTTATGGGGATTCCATGTTT
58     ATATTTTTCCATCAAGAGTCCCAATAACAATAAAAAAGTATT.....AGTTCCCAAGGTA

              250       260       270       280           290
18     TTTTGCTTACGGCGTGAGCAGCTTTTTGCTAGGCATTTTTGG....AATAGAGCAGGTAC
58     TCAGGCTTACAGTAT.AGGGTCTTTAGGGTGCGTTTACCTGATCCCAATAAATTTGGTTT

        300       310       320       330       340       350
18     TATGGGTGACACTGTGCCTCAATCCTTATATATTAAAGGCACAGGTATGCGTGCTTCACC
58     TCCTGAT.ACATCTTTTTATAACCCTGATACAC.AACGTTTGGTCTGGGCATGTGTAGGC

        360       370       380       390       400       410
18     TGGCAGCTGTGTGTATTCTCCCTCTCCAAGTGGCTCTATTGTTACCTCTGACTCCCAGTT
58     CTTGAAATAGGTAGAGGACAGCCATTGG................................

18     G
58     .
```

```
       1        10         20         30         40         50         60
31     CTGTCCCAGTGTCTAAAGTTGTAAGCACGGATGAATATGTAACACGAACCAACATATATT
33     ....................................................CTTGAAAT

                 70         80         90        100        110        120
31     ATCACGCAGGCAGTGCTAGGCTGCTTACAGTAGGCCATCCATATTATTCCATACCTAAAT
33     AGGTAGAGGGCAGCCATTAGGCGTTGGCA.TAAGTGGTC...ATCCTTTATTAAACAAAT

               130        140        150        160        170
31     CTGACAATCCTAAAAA.AATAGTT.GTACCAAAGGTGTCAGGATTACAATATAGGGTATT
33     TTGATGACACTGAAACCAGTAACAAGTATCCTGGACAACCGGGTGCTGATAATAGGGAAT

      180        190        200        210        220        230
31     TAGGGTTCGTTTACCAGATCCAAACAAATTTGGATTTCCTGATACATCTTTTTATAATCC
33     GTTTATCCATGGATTATAAACAAACACAGTTATGTTTACTTGGATGTAAGCCTCCAACAG

      240        250        260        270        280        290
31     TGAAAC.TCAACGCTTAGTTTGGGCCTGTGTTGGTTTAGAGGTAGGTCGCGGGCAGCCAT
33     GGGAACATTGGGGTAAAGGTGTTGCTTGTACTAATGCAGCACCTGCCAATGATTGTCCAC

       300        310
31     TAGGTGTAGGTATTAGTG............
33     CTTTAGAACTTATAAATACTATTATTGAGG
```

```
                                          1         10        20
31     .............................CTG..TCCCAGTGTCTAAAGTTGTAAGCACG
45a    CCCTTCTCCCAGTGGCTCTATTATTACTTCTGATTCTCAATTATTTAATAAGCCATATTG

       30        40             50        60        70        80
31     GATGAATATGTAACACG.....AACCAACATATATTATCACGCAGGCAGTGCTAGGCTGC
45a    GTTACATAAGGCCCAGGGCCATAACAATGGTATTTGTTGGCATAATCAGTTGTTTGTTAC

            90        100       110       120       130       140
31     TTACAGTAGGCATCCATATTATTCCATACCTAAATCTGACAATCCTAAAAAAATAGTTG
45a    T.GTAGTGGACACTACCCGCAGTACTAATTTAACATTATGTGCCTCTACACAAAATTCTG

           150       160          170       180       190       200
31     TACCAAAGGTGTCAGGAT.TAC..AATATAGGGTATTTAGGGTTCGTTTACCAGATCCAA
45a    TGCCAAGTACATATGACCCTACTAAGTTTAAGCAGTATAGTAGACATGTGGAGGA...AT

              210       220       230       240        250       260
31     ACAAATTTGGATTTCCTGATACATCTTTTTATAATCCTGAAACT.CAACGCTTAGTTTGG
45a    ATGATTTACAGTTTATTTTCAGTTGTGCACTATTACTTTAACTGCAGAGGTTATGTCAT

               270       280       290       300        310
31     GCCTGTGTTGGTTTAGAGGTAGGTCGCGGGCAGCCATTAGGTGTAGGTATTAGTG.....
45a    ATATCCATAGTATGAATAGTAGTATATTAGAAAAT.TGGAATTTTGGTGTCCCTCCACCA

31     ................
45a    CCTACTACAAGTTTGG
```

```
       1        10        20        30        40        50
31     CTGTCCCAGTGTCTAAAGTTGTAAGCACGGATGAATATGTAACA.CGAACCAACATATAT
45b    ......CAGGATACAAAGTGCGAGGTTCCATTAGACATTTGTCAATCCATCTGTAAATAT

      60        70        80        90       100       110
31     TATCAC.GCAGGCAGTGCTAGGCTGCTTACAGTAGGCCATCCATATTATTCCATACCTAA
45b    CCAGATTATTTGCAAATGTCTGCTGATCCCTATGGGATTCTATGTTTTTTTGCCTACGC

      120       130       140       150       160       170
31     ATCTGACAATCCTAAAAAAAATAGTTGTACCAAAGGTGTCAGGATTACAAT..ATAGGGTA
45b    CGTGAACAACTGTTTGCAAGACATTTTTGGAATAGGGCAGGTGTTATGGGTGACACAGTA

         180       190       200       210       220       230
31     TTTA.GGGTTCGTTTACCAGATCCAAACAAATTTGGATTTCCTGATACATCTTTTTATAA
45b    CCTACGGACCTATATATTAAAGGCAC.TAGCGCTAATATGCGTGAAACCCCTGGCAGTTG

          240       250       260       270       280       290
31     TCCTGAAACTCAACGCTTAGTTTGGGCCTGTGTTGGTTTAGAGGTAGGTCGCGGGCAGCC
45b    TGTGTATTCCCCTTCTCCCAGTGGCTCTATTATTACTTCTG...................

          300       310
31     ATTAGGTGTAGGTATTAGTG
45b    ....................
```

```
                   1        10        20        30        40
31     ...........CTGTCCCAGTGTCTAAAGTTGTAAGCACGGAT...GAATATGTAACACG
45c    CAACCTGGTGACTGTCCTCCT.TTGGAACTTAAAAACACCATTATTGAGGATGGTGATAT

           50        60        70        80        90       100
31     AACCAACATATATTATCACGCAGGCAGTGCTAGGCTGCTTACAGTAGGCCATCCATATTA
45c    GGTGGATACAGGTTATGGGGCAATGGATTTTAGTACA.TTGCAGGATACAAAGTGCGAGG

          110       120       130            140       150       160
31     TTCCATACCTAAATCTGACAATCC.....TAAAAAAATAGTTGTACCAAAGGTGTCAGGA
45c    TTCCAT.TAGACATTTGTCAATCCATCTGTAAATATCCAGATTATTTGCAAATGTCTGCT

               170       180       190       200       210       220
31     TTACAATATAGGGTATTTAGGGTTCGTTTACCAGATCCAAACAAATTTGGATTTCCTGAT
45c    GATCCCTATGGGG.ATTCTATGTTTTTTTGCCTACGCCGTGAACAACTG...TTTGCAAG

               230       240       250       260       270       280
31     ACATCTTTTTATAATCCTGAAACTCAACGCTTAGTTTGGGCCTGTGTTGGTTTAGAGGTA
45c    ACATTTTTGGAATAGGGCAGGTGTTATGGGTGACACAGTACCTACGGACCTATATATTAA

               290        300       310
31     GGTCGCGGGCAGCCAT.TAGGTGTAGGTATTAGTG..........................
45c    AGGCACTAGCGCTAATATGCGTGAAACCCCTGGCAGTTGTGTGTATTCCCCTTCTCCCAG

31     ....................
45c    TGGCTCTATTATTACTTCTG
```

```
         1       10        20        30        40        50        60
31       CTGTCCCAGTGTCTAAAGTTGTAAGCACGGATGAATATGTAACACGAACCAACATATATT
52       .....CCTGTCTCTAAGGTTGTAAGCACTGATGAGTATGTGTCTCGCACAAGCATCTATT

               70        80        90       100       110       120
31       ATCACGCAGGCAGTGCTAGGCTGCTTACAGTAGGCCATCCATATTATTCCATACCTAAAT
52       ATTATGCAGGCAGTTCTCGATTACTAACAGTAGGACATCCCTATTTTTCTATTAAAAACA

                   130       140       150       160       170
31       C......TGACAATCCTAAAAAAATAGTTGTACCAAAGGTGTCAGGATTACAATATAGGG
52       CCAGTAGTGGTAATGGTAAAAAAGTTTTAGTTCCCAAGGTGTCTGGCCTGCAATACAGGG

           180       190       200       210       220       230
31       TATTTAGGGTTCGTTTACCAGATCCAAACAAATTTGGATTTCCTGATACATCTTTTTATA
52       TATTTAGAATTAAATTGCCGGACCCTAATAAATTTGGTTTTCCAGATACATCTTTTTATA

           240       250       260       270       280       290
31       ATCCTGAAACTCAACGCTTAGTTTGGGCCTGTGTTGGTTTAGAGGTAGGTCGCGGGCAGC
52       ACCCAGAAACCCAAAGGTTGGTGTGGGCCTGTACAGGCTTGGAAATTGGTAGGGGACAGC

           300       310
31       CATTAGGTGTAGGTATTAGTG..
52       CTTTAGGTGTGGGTATTAGTGGG
```

```
              1        10        20        30        40        50
31     ...CTGTCCCAGTGTCTAAAGTTGTAAGCACGGATGAATATGTAACACGAACCAACATAT
58     CTCCTGTGCCTGTGTCTAAGGTTGTAAGCACTGATGAATATGTGTCACGCACAAGCATTT

            60        70        80        90       100       110
31     ATTATCACGCAGGCAGTGCTAGGCTGCTTACAGTAGGCCATCCATATTATTCCATACCTA
58     ATTATTATGCTGGCAGTTCCCGACTTTTGGCTGTTGGCAATCCATATTTTTCCATCAAGA

           120       130       140       150       160       170
31     AATCTGACAATCCTAAAAAAATAGTTGTACCAAAGGTGTCAGGATTACAATATAGGGTAT
58     GTCCCAATAACAATAAAAAAGTATTAGTTCCCAAGGTATCAGGCTTACAGTATAGGGTCT

           180       190       200       210       220       230
31     TTAGGGTTCGTTTACCAGATCCAAACAAATTTGGATTTCCTGATACATCTTTTTATAATC
58     TTAGGGTGCGTTTACCTGATCCCAATAAATTTGGTTTTCCTGATACATCTTTTTATAACC

           240       250       260       270       280       290
31     CTGAAACTCAACGCTTAGTTTGGGCCTGTGTTGGTTTAGAGGTAGGTCGCGGGCAGCCAT
58     CTGATACACAACGTTTGGTCTGGGCATGTGTAGGCCTTGAAATAGGTAGAGGACAGCCAT

           300       310
31     TAGGTGTAGGTATTAGTG
58     TGG...............
```

```
       1        10        20        30        40        50        60
45a    CCCTTCTCCCAGTGGCTCTATTATTACTTCTGATTCTCAATTATTTAATAAGCCATATTG
52     ................................CCTGTCTCTAAGGTTGTAAGCACTGATG

                 70        80        90       100       110
45a    GTTACATAAGGCCCAGGGCCATAACAATGGTATTTGTTGGCA.TAATCAGTTGTTTGTTA
52     AGTATGTGTCTCGCACAAGCAT..CTATTAT.TATGCAGGCAGTTCTCGATTACTAACAG

     120       130       140       150       160       170
45a    CTGTAGTGGACACTACCCGCAGTACTAATTTAACATTATGTGCCTCTACACAAAATTCTG
52     TAGGACATCCCTATTTTTCTATTAAAAACACCAGTAGTGGTAATGGTAAAAAAGTTTTAG

     180       190       200       210       220       230
45a    TGCCAAGTACATATGACCCTACTAAGTTTAAGCAGTATAGTAGACATGTGGAGGAATATG
52     TTCCCAAGGTGTCTGGCCTG...CAATACAGGGTATTTAGAATTAAATTGCCGGACCCTA

     240       250       260       270           280       290
45a    ATTTACAGTTTATTTTTCAGTTGTGCACTATTA....CTTTAACTGCAGAGGTTATGTCA
52     A..TAAATTTGGTTTTCCAGATACATCTTTTTATAACCCAGAAACCCAAAGGTTGGTGTG

         300        310       320       330       340       350
45a    TATATCCATAG.TATGAATAGTAGTATATTAGAAAATTGGAATTTTGGTGTCCCTCCACC
52     GGCCTGTACAGGCTTGGAAATTGGTAGGGGACAGCCTTTAGGTGTGGGTATTAGTGGG..

          360       370
45a    ACCTACTACAAGTTTGG
52     .................
```

```
        1        10        20        30        40        50        60
45a     CCCTTCTCCCAGTGGCTCTATTATTACTTCTGATTCTCAATTATTTAATAAGCCATATTG
58      ........................................................CTCC

                 70        80        90       100       110
45a     GTTACATAAGGCCCAGGGCCATAACAATGGTATTTGT.TGGCATAATCAGTTGTTTGTTA
58      TGTGCCTGTGTCTAAGGTTGTAAGCACTGATGAATATGTGTCACGCACAAGCATTTATTA

      120       130       140       150       160       170
45a     CTGTAGTGGACACTACCCGCAGTACTAATTTAACATTATGTGCCTCTACACAAATTCTG
58      TTATGCTGGCAGTTCCCGACTTTTGGCTGTTGGCAATCCATATTTTTCCATCAAGA...G

      180       190        200       210       220       230
45a     TGCCAAGTACA.TATGACCCTACTAAGTTTAAGCAGTATAGTAGACATGTGGAGGAATAT
58      TCCCAATAACAATAAAAAGTATTAGTTCC...CAAGGTATCAGGCTTACAGTATAGGGT

       240       250       260       270       280       290
45a     GATTTACAGTTTATTTTTCAGTTGTGCACTATTACTTTAACTGCAGAGGTTATGTCATAT
58      C.TTTAGGGTGCGTTTACCTGATCC.CAATAAATTTGGTTTTCCTGATACATCTTTTTAT

       300       310       320       330       340       350
45a     ATCCATAGTATGAATAGTAGTATATTAGAAATTGGAATTTTGGTGTCCCTCCACCACCT
58      AACCCTGATACACAACGTTTGGTCTGGGCATGTGTAGGCCTTGAAATAGGTAGAGGACA.

       360       370
45a     ACTACAAGTTTGG
58      GCCATTGG.....
```

```
        1        10        20        30        40        50
45b    .CAGGATACAAAGTGCGAGGTTCCATTAGACATTTGTCAATCCATCTGTAAATATCCAGA
52     CCTGTCTCTAAGGTTGTAAGCACTGATGAGTATGTGTCT.CGCACAAGCATCTATTATTA

       60        70        80        90        100       110
45b    TTATTTGCAAATGTCTGCTGATCCCTATGGGATTCTATGTTTTTTGCCTACGCCGTGA
52     T.GCAGGCAGTTCTCGATTACTAACAGTAGGACATCCCTATTTTTCTATTAAAAACACCA

      120       130       140       150       160       170
45b    ACAACTGT..TTGCAAGACATTTTTGGAATAGGGCAGGTGTTATGGGTGACACAGTACCT
52     GTAGTGGTAATGGTAAAAAAGTTTTAGTTCCCAAGGTGTCTGGCCTGCAATACAGGGTAT

       180       190       200       210       220       230
45b    ACGGACCTATATATTAAAGGCACTAGCGCTAATATGCGTGAAACCCCTGGCAGTTGTGTG
52     TTAGA.......ATTAAATTGCCGGACCCTAATAAATTTGGTTTTCCAGATACATCTTTT

       240       250       260       270
45b    TATTCCCCTTCTCCCAGTGGCTCTATTATTACTTCTG........................
52     TATAACCCAGAAACCCAAAGGTTGGTGTGGGCCTGTACAGGCTTGGAAATTGGTAGGGGA

45b    ............................
52     CAGCCTTTAGGTGTGGGTATTAGTGGG
```

```
                  1        10        20        30        40        50
45b    .........CAGGATACAAAGTGCGAGGTTCCATTAGACATTTGTCAATCCATCTGTAAA
58     CTCCTGTGCCTGTGTCTAAGGTTGTAAGCACTGATGAATATGTGTCA.CGCACAAGCATT

                60        70        80        90       100       110
45b    TATCCAGATTATTTGCAAATGTCTGCTGATCCCTATGGGATTCTATGTTTTTTTGCCTA
58     TATTATTAT.GCTGGCAGTTCCCGACTTTTGGCTGTTGGCAATCCATATTTTTCCATCAA

               120       130       140       150       160       170
45b    CGCCGTGAACAACTGTTTGCAAGACATTTTTGGAATAGGGCAGGTGTTATGGGTGACACA
58     GAGTCCCAATAACAATAAAAAAGTATTAGTTCCCAAGGTATCAGGCTTACAGT..ATAGG

               180       190       200       210       220       230
45b    GTACCTACGGACCTATATATTAAAGGCACTAGCGCTAATATGCGTGAAACCCCTGGCAGT
58     GTCTTTAGGGTGCGTTTACCTGATCCCAATAAATTTGGTTTCCTGATACATCTTTTTAT

               240       250       260       270
45b    TGTGTGTATTCCCTTCTCCCAGTGGCTCTATTATTACTTCTG..................
58     AACCCTGATACACAACGTTTGGTCTGGGCATGTGTAGGCCTTGAAATAGGTAGAGGACAG

45b    .......
58     CCATTGG
```

```
     1        10        20        30        40        50        60
45c  CAACCTGGTGACTGTCCTCCTTTGGAACTTAAAAACACCATTATTGAGGATGGTGATATG
52   ...............CCTGTCTCTAAGGTTGTAAGCAC...TGATGAGTATGTGTCTCGC

              70        80        90       100       110
45c  GTGGATACAGGTTATGGGGCAATGGATTTTAGTACATTG.CAGGATACAAAGTGCGAGGT
52   ACAAGCATCTATTATTATGCAGGCAGTTCTCGATTACTAACAGTAGGACATCCCTATTTT

   120       130       140       150       160       170
45c  TCCATTAGA..CATTTGTCAATCCATCTGTAAATATCCAGATTATTTGCAAATGTCTGCT
52   TCTATTAAAAACACCAGTAGTGGTAATGGTAAAAAAGTTTTAGTTCCCAAGGTGTCTGGC

     180       190       200       210       220          230
45c  GATCCCTATGGGGATTCTATGTTTTTTTGCCTACGCCGTGAACAACT..GTTTGCAAGAC
52   CTGCAATACAGGGTATTTAGAATTAAATTGCCGGACCCTAATAAATTTGGTTTTCCAGAT

        240       250         260       270        280       290
45c  AT.TTTTGGAATAGGGCAGGTGTT..ATGGTGACACAGTACC.TACGGACCTATATATT
52   ACATCTTTTTATAACCCAGAAACCCAAAGGTTGGTGTGGGCCTGTACAGGCTTGGAAATT

             300       310       320       330       340       350
45c  AA.AGGCACTAGCGCTAATATGCGTGAAACCCCTGGCAGTTGTGTGTATTCCCCTTCTCC
52   GGTAGGGGACAGCCTTTAGGTGTGGGTATTAGTGGG........................

             360       370
45c  CAGTGGCTCTATTATTACTTCTG
52   .......................
```

```
       1        10        20        30        40        50
45c    CAACCTGGTGACTGTCCTCCTTTGGAACTTAAAAACACCATTATTGAGGATGGTGATA.T
58     ............................CTCCTGTGCCTGTGTCTAAGGTTGTAAGCACT

      60        70        80        90       100       110
45c    GGTGGATACAGGTTATGGGGCAATGGATTTTAGTACATTGCAGGATACAAAGTGCGAGGT
58     GATGAATATGTGTCACGCACAAGCATTTATTATTATGCTGGCAGTTCCCGACTTTTGG..

     120       130       140       150       160       170
45c    TCCATTAGACATTTGTCAATCCATCTGTAAATATCCAGATTATTTGCAAATGTCTGCTGA
58     .CTGTTGGCAATCCAT..ATTTTTCCATCAAGAGTCCCAATAACAATAAAAAAGTATTAG

     180       190       200       210       220       230
45c    TCCCTATGGGGATTCTATGTTTTTTTGCCTACGCCGTGAACAACTGTTTGCAAGACATTT
58     TTCCCAAGG...TATCAGGCTTACAGTATAGGGTCTTTAGGGTGCGTTTACCTGATCCC.

     240       250       260       270       280       290
45c    TTGGAATAGGGCAGGTGTTATGGGTGACACAGTACCTACGGACCTATATATTAAAGGCAC
58     ....AATAAATTTGGTTTTCC...TGATACATCTTTTTATAACCCTGATACACAACGT.T

     300       310       320       330       340       350
45c    TAGCGCTAATATGCGTGAAACCCCTGGCAGTTGTGTGTATTCCCCTTCTCCCAGTGGCTC
58     TGGTCTGGGCATGTGTAGGCCTTGAAATAGGTA.GAGGACAGCCATTGG...........

     360       370
45c    TATTATTACTTCTG
58     ..............
```

```
                1       10        20        30        40        50
52      ........CCTGTCTCTAAGGTTGTAAGCACTGATGAGTATGTGTCTCGCACAAGCATCT
58      CTCCTGTGCCTGTGTCTAAGGTTGTAAGCACTGATGAATATGTGTCACGCACAAGCATTT

              60        70        80        90       100       110
52      ATTATTATGCAGGCAGTTCTCGATTACTAACAGTAGGACATCCCTATTTTTCTATTAAAA
58      ATTATTATGCTGGCAGTTCCCGACTTTGGCTGTTGGCAATCCATATTTTTCCATCAAGA

             120       130       140       150       160       170
52      ACACCAGTAGTGGTAATGGTAAAAAAGTTTTAGTTCCCAAGGTGTCTGGCCTGCAATACA
58      GTCCCAATA......ACAATAAAAAAGTATTAGTTCCCAAGGTATCAGGCTTACAGTATA

             180       190       200       210       220       230
52      GGGTATTTAGAATTAAATTGCCGGACCCTAATAAATTTGGTTTTCCAGATACATCTTTTT
58      GGGTCTTTAGGGTGCGTTTACCTGATCCCAATAAATTTGGTTTTCCTGATACATCTTTTT

             240       250       260       270       280       290
52      ATAACCCAGAAACCCAAAGGTTGGTGTGGGCCTGTACAGGCTTGGAAATTGGTAGGGGAC
58      ATAACCCTGATACACAACGTTTGGTCTGGGCATGTGTAGGCCTTGAAATAGGTAGAGGAC

             300       310
52      AGCCTTTAGGTGTGGGTATTAGTGGG
58      AGCCATTGG.................
```

```
                                                                                                     1        10
6a    .....................................................................................CCTAACCCTGTAT
6b    ..................................................................................................
11a   ..................................................................................................
11b   ..................................................................................................
16    ......................................................................................CTGTCCCAGTAT
18    CACTGGGCTAAAGGCACTGCTTGTAAATCGCGTCCTTTATCACAGGGCGATTGCCCCCCTTTAGAACTTAAAAACACAGTTTTGGAAGATGGTGATAT
31    ......................................................................................CTGTCCCAGTGT
33    ..................................................................................................
45a   .............................................................................CCCTTCTCCCAGTGGCTCTAT
45b   ..................................................................................................
45c   .......................................CAACCTGGTGACTGTCCTCCTTTGGAACTTAAAAACACCATTATTGAGGATGGTGATAT
52    ...........................................................................................CCTGTCT
58    ...................................................................................CTCCTGTGCCTGTGT

            20        30        40        50        60        70        80        90       100       110
6a    CCAAAGTTGTTGCCACGGATGCTTATGTTACTCGCACCAACATATTTTATCATGCCAGCAGTTCTAGACTTCTTGCAGTGGGTCATCCTTATTTTTCC
6b    .........................................................CAGCCATTAGGTGTGGGT.GTAAGTGGACATCCTTTTCCTA
11a   CCAAGGTTGTTGCCACGGATGCGTATGTTAAACGCACCAACATATTTTATCATGCCAGCAGTTCTAGACTCCTTGCTGTGGGACATCCATATTACTCT
11b   .............................................................CCTTTAGGCGTTGGT.GTTAGTGGGCATCCATTGCTA
16    CTAAGGTTGTAAGCACGGATGAATATGTTGCACGCACAAACATATATTATCATGCAGGAACATCCAGACTACTTGCAGTTGGACATCCCTATTTTCCT
18    GGTAGATACTGGATATGGTGCCATGGACTTTAGTACATTGCAAGATACTAAATGTGAGGTACCATTGGATATTTGT..CAGTCTATTTGTAAATATCC
31    CTAAAGTTGTAAGCACGGATGAATATGTAACACGAACCAACATATATTATCACGCAGGCAGTGCTAGGCTGCTTACAGTAGGCCATCCATATTATTCC
33    ........................................CTTGAAATAGGTAGAGGGCAGCCATTAGGCGTTGGC.ATAAGTGGTCATCCTTTATTA
45a   TATTACTTCTGATTCTCAATTATTTAATAAGCCATATTGGTTACATAAGGCCCAGGGCCATAACAATGGTATTTGTT.GGCATAATCAGTTGTTTGTT
45b   ........................................CAGGATACAAAGTGCGAGGTTCCATTAGACATTTGT..CAATCCATCTGTAAATATCC
45c   GGTGGATACAGGTTATGGGGCAATGGATTTTAGTACATTGCAGGATACAAAGTGCGAGGTTCCATTAGACATTTGT..CAATCCATCTGTAAATATCC
52    CTAAGGTTGTAAGCACTGATGAGTATGTGTCTCGCACAAGCATCTATTATTATGCAGGCAGTTCTCGATTACTAACAGTAGGACATCCCTATTTTTCT
58    CTAAGGTTGTAAGCACTGATGAATATGTGTCACGCACAAGCATTTATTATTATGCTGGCAGTTCCCGACTTTTGGCTGTTGGCAATCCATATTTTTCC

                120                                130       140       150                 160       170       180
6a    ATAAAACGGGCTAACA...............AAACTGTTGTGCCAAAGGTGTCAGGATATC.....AATACAGGGTATTTAAGGTGGTGTTACCAGAT
6b    AATAAATATGATGATGTTGAAAATTCAGGG.AG...TGGTGGTAACCCTGGACAGGATAAC.....AGGGTTAATGTTGGTATGGATTATAAACAAAC
11a   ATCAAAAAAGTTAACA...............AAACAGTTGTACCAAAGGTGTCTGGATATC.....AATATAGAGTGTTTAAGGTAGTGTTGCCAGAT
11b   AACAAATATGATGATGTAGAAAATAGTGGT.GGGTATGGTGGTAATCCTGGTCAGGATAAT.....AGGGTTAATGTAGGTATGGATTATAAACAAAC
16    ATTAAAAAACCTAACA.........ATAACAAAATATTAGTTCCTAAAGTATCAGGATTAC.....AATACAGGGTATTTAGAATACATTTACCTGAC
18    TGATTATTTACAAATGTCTGCAGATCCTTATGGGGATTCCATGTTTTTTTGCTTACGGCGT.....GAGCAGCTTTTTGCTAGGCATTTTTGGAATAG
31    ATACCTAAATCTGACA......ATCCTAAAAAAATAGTTGTACCAAAGGTGTCAGGATTAC.....AATATAGGGTATTTAGGGTTCGTTTACCAGAT
33    AACAAATTTGATGACACTGAAACCAGTAAC.AAGTATCCTGGACAACCGGGTGCTGATAAT.....AGGGAATGTTTATCCATGGATTATAAACAAAC
45a   ACTGTAGTGGACACTACCCGCAGTACTAATTTAACATTATGTGCCTCTACACAAAATTCTGTGCCAAGTACATATGACCCTACTAAGTTTAAGCAGTA
45b   AGATTATTTGCAAATGTCTGCTGATCCCTATGGGGATTCTATGTTTTTTTGCCTACGCCGT.....GAACAACTGTTTGCAAGACATTTTTGGAATAG
45c   AGATTATTTGCAAATGTCTGCTGATCCCTATGGGGATTCTATGTTTTTTTGCCTACGCCGT.....GAACAACTGTTTGCAAGACATTTTTGGAATAG
52    ATTAAAAACACCAGTAGTGGTAATGGTAAAAAAGTTTTAGTTCCCAAGGTGTCTGGCCTGC.....AATACAGGGTATTTAGAATTAAATTGCCGGAC
58    ATCAAGAGTCCCAATA......ACAATAAAAAAGTATTAGTTCCCAAGGTATCAGGCTTAC.....AGTATAGGGTCTTTAGGGTGCGTTTACCTGAT

        190       200       210       220       230       240       250       260       270       280
6a    CCTAACAAATTTGCATTGCC.TGACTCGTCTCTTTTTGATCCCACAACACAACGTTTGGTATGGGCATGCACAGGCCTAGAGGTGGGCA.GGGGACAG
6b    ACAATTATGCATGGTTGGATGTGCCCCCCCTTTGGGCGAGCATTGGGGTAAAGGTAAACAGTGTACTAATACACCTGTACAGGCTGGTG.ACTGCCCG
11a   CCTAACAAGTTTGCATTACC.TGATTCATCTCTGTTTGACCCCACTACACAGCGTTTAGTATGGGCGTGCACAGGGTTGGAGGTAGGCA.GGGGTCAA
11b   CCAGCTATGTATGGTGGGCTGTGCTCCACCGTTAGGTGAACATTGGGGTAAGGGTACACAATGTTCAAATACCTCTGTACAAAATGGTG.ACTGCCCC
16    CCCAATAAGTTTGGTTTTCC.TGACACCTCATTTTATAATCCAGATACACAGCGGCTGGTTTGGGCCTGTGTAGGTGTTGAGGTAGGTC.GTGGTCAG
18    AGCAGGTACTATGGGTGACA.CTGTGCCTCAATCCTTATATATTAAAGGCACAGG......TATGCGTGCTTCACCTGGCAGCTGTGTGTATTCTCCC
31    CCAAACAAATTTGGATTTCC.TGATACATCTTTTTATAATCCTGAAACTCAACGCTTAGTTTGGGCCTGTGTTGGTTTAGAGGTAGGTC.GCGGGCAG
33    ACAGTTATGTTTACTTGGATGTAAGCCTCCAACAGGGGAACATTGGGGTAAAGGTGTTGCTTGTACTAATGCA...GCACCTGCCAATG.ATTGTCCA
45a   TAGTAGACATGTGGAGGAATATGATTTACAGTTTATTTTTCAGTTGTGCACTATTACTTTAACTGCAGAGGTTATGTCATATATCCATAGTATGAATA
45b   GGCAGGTGTTATGGGTGACA.CAGTACCTACGGACCTATATATTAAAGGCACTAGCGCTAATATGCGTGAAACCCCTGGCAGTTGTGTGTATTCCCCT
45c   GGCAGGTGTTATGGGTGACA.CAGTACCTACGGACCTATATATTAAAGGCACTAGCGCTAATATGCGTGAAACCCCTGGCAGTTGTGTGTATTCCCCT
52    CCTAATAAATTTGGTTTTCC.AGATACATCTTTTTATAACCCAGAAACCCAAAGGTTGGTGTGGGCCTGTACAGGCTTGGAAATTGGTA.GGGGACAG
58    CCCAATAAATTTGGTTTTCC.TGATACATCTTTTTATAACCCTGATACACAACGTTTGGTCTGGGCATGTGTAGGCCTTGAAATAGGTA.GAGGACAG

         290       300
6a    CCATTAGGTGTGGGTGTAAG..............................................................................
6b    CCCTTAGAACTTATTACCAG..............................................................................
11a   CCTTTAGGCGTTGGTGTTAGTGGGCATCCATTGCTAAACAAATATGATGATGTAGAAAATAGTGGTGGGTATGGTGGTAATCCTGGTCAG........
11b   CCGTTGGAACTTATTACCAGTGTTATACAGGATGGGGACATGGTTGATACAGGCTTTGGTGCTATGAATTTTGCAGACTTACAAACCAATAAATCGGA
16    CCATTAG...........................................................................................
18    TCTCCAAGTGGCTCTATTGTTACCTCTGACTCCCAGTTG...........................................................
31    CCATTAGGTGTAGGTATTAGTG............................................................................
33    CCTTTAGAACTTATAAATACTATTATTGAGG...................................................................
45a   GTAGTATATTAGAAAATTGGAATTTTGGTGTCCCTCCACCACCTACTACAAGTTTGG.........................................
45b   TCTCCCAGTGGCTCTATTATTACTTCTG......................................................................
45c   TCTCCCAGTGGCTCTATTATTACTTCTG......................................................................
52    CCTTTAGGTGTGGGTATTAGTGGG..........................................................................
58    CCATTGG...........................................................................................
```

**6a**	..
**6b**	..
**11a**	..
**11b**	TG
**16**	..
**18**	..
**31**	..
**33**	..
**45a**	..
**45b**	..
**45c**	..
**52**	..
**58**	..
