# Supplementary material for: Microfluidic space coding for multiplexed nucleic acid detection via CRISPR-Cas12a and recombinase polymerase amplification
Source: Nat Commun. 2022 Oct 29;13:6480. doi: 10.1038/s41467-022-34086-y (PMC9617605; doi:10.1038/s41467-022-34086-y)
Supplement: Supplementary file 6 — Supplementary Data 4 [file 41467_2022_34086_MOESM6_ESM.pdf]

Sequence alignment of Top 5 amplicons of 9 HPV subtypes

*Sequences in black box are crRNA binding regions.

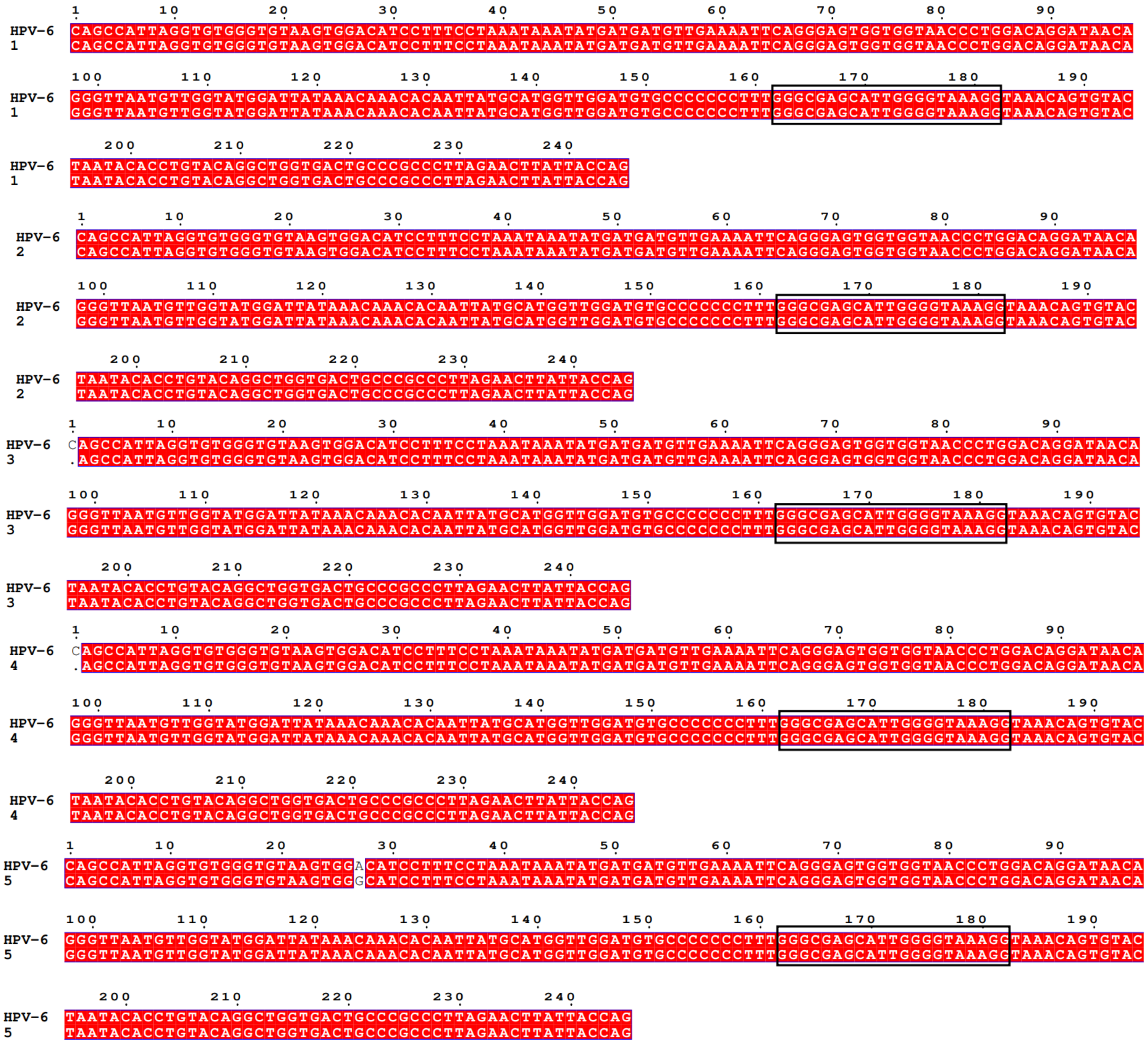
HPV-6 1
CAGCCATTAGGTGTGGGTGTAAGTGGACATCCTTTCCTAAATAAATATGATGATGTTGAAAATTCAGGGAGTGGTGGTAACCCTGGACAGGATAACA
CAGCCATTAGGTGTGGGTGTAAGTGGACATCCTTTCCTAAATAAATATGATGATGTTGAAAATTCAGGGAGTGGTGGTAACCCTGGACAGGATAACA
GGGTTAATGTTGGTATGGATTATAAACAAACACAATTATGCATGGTTGGATGTGCCCCCCCTTTGGGCGAGCATTGGGGTAAAGGTAAACAGTGTAC
GGGTTAATGTTGGTATGGATTATAAACAAACACAATTATGCATGGTTGGATGTGCCCCCCCTTTGGGCGAGCATTGGGGTAAAGGTAAACAGTGTAC
TAATACACCTGTACAGGCTGGTGACTGCCCGCCCTTAGAACTTATTACCAG
TAATACACCTGTACAGGCTGGTGACTGCCCGCCCTTAGAACTTATTACCAG
HPV-6 2
CAGCCATTAGGTGTGGGTGTAAGTGGACATCCTTTCCTAAATAAATATGATGATGTTGAAAATTCAGGGAGTGGTGGTAACCCTGGACAGGATAACA
CAGCCATTAGGTGTGGGTGTAAGTGGACATCCTTTCCTAAATAAATATGATGATGTTGAAAATTCAGGGAGTGGTGGTAACCCTGGACAGGATAACA
GGGTTAATGTTGGTATGGATTATAAACAAACACAATTATGCATGGTTGGATGTGCCCCCCCTTTGGGCGAGCATTGGGGTAAAGGTAAACAGTGTAC
GGGTTAATGTTGGTATGGATTATAAACAAACACAATTATGCATGGTTGGATGTGCCCCCCCTTTGGGCGAGCATTGGGGTAAAGGTAAACAGTGTAC
TAATACACCTGTACAGGCTGGTGACTGCCCGCCCTTAGAACTTATTACCAG
TAATACACCTGTACAGGCTGGTGACTGCCCGCCCTTAGAACTTATTACCAG
HPV-6 3
CAGCCATTAGGTGTGGGTGTAAGTGGACATCCTTTCCTAAATAAATATGATGATGTTGAAAATTCAGGGAGTGGTGGTAACCCTGGACAGGATAACA
.AGCCATTAGGTGTGGGTGTAAGTGGACATCCTTTCCTAAATAAATATGATGATGTTGAAAATTCAGGGAGTGGTGGTAACCCTGGACAGGATAACA
GGGTTAATGTTGGTATGGATTATAAACAAACACAATTATGCATGGTTGGATGTGCCCCCCCTTTGGGCGAGCATTGGGGTAAAGGTAAACAGTGTAC
GGGTTAATGTTGGTATGGATTATAAACAAACACAATTATGCATGGTTGGATGTGCCCCCCCTTTGGGCGAGCATTGGGGTAAAGGTAAACAGTGTAC
TAATACACCTGTACAGGCTGGTGACTGCCCGCCCTTAGAACTTATTACCAG
TAATACACCTGTACAGGCTGGTGACTGCCCGCCCTTAGAACTTATTACCAG
HPV-6 4
CAGCCATTAGGTGTGGGTGTAAGTGGACATCCTTTCCTAAATAAATATGATGATGTTGAAAATTCAGGGAGTGGTGGTAACCCTGGACAGGATAACA
.AGCCATTAGGTGTGGGTGTAAGTGGACATCCTTTCCTAAATAAATATGATGATGTTGAAAATTCAGGGAGTGGTGGTAACCCTGGACAGGATAACA
GGGTTAATGTTGGTATGGATTATAAACAAACACAATTATGCATGGTTGGATGTGCCCCCCCTTTGGGCGAGCATTGGGGTAAAGGTAAACAGTGTAC
GGGTTAATGTTGGTATGGATTATAAACAAACACAATTATGCATGGTTGGATGTGCCCCCCCTTTGGGCGAGCATTGGGGTAAAGGTAAACAGTGTAC
TAATACACCTGTACAGGCTGGTGACTGCCCGCCCTTAGAACTTATTACCAG
TAATACACCTGTACAGGCTGGTGACTGCCCGCCCTTAGAACTTATTACCAG
HPV-6 5
CAGCCATTAGGTGTGGGTGTAAGTGGACATCCTTTCCTAAATAAATATGATGATGTTGAAAATTCAGGGAGTGGTGGTAACCCTGGACAGGATAACA
CAGCCATTAGGTGTGGGTGTAAGTGGGCATCCTTTCCTAAATAAATATGATGATGTTGAAAATTCAGGGAGTGGTGGTAACCCTGGACAGGATAACA
GGGTTAATGTTGGTATGGATTATAAACAAACACAATTATGCATGGTTGGATGTGCCCCCCCTTTGGGCGAGCATTGGGGTAAAGGTAAACAGTGTAC
GGGTTAATGTTGGTATGGATTATAAACAAACACAATTATGCATGGTTGGATGTGCCCCCCCTTTGGGCGAGCATTGGGGTAAAGGTAAACAGTGTAC
TAATACACCTGTACAGGCTGGTGACTGCCCGCCCTTAGAACTTATTACCAG
TAATACACCTGTACAGGCTGGTGACTGCCCGCCCTTAGAACTTATTACCAG

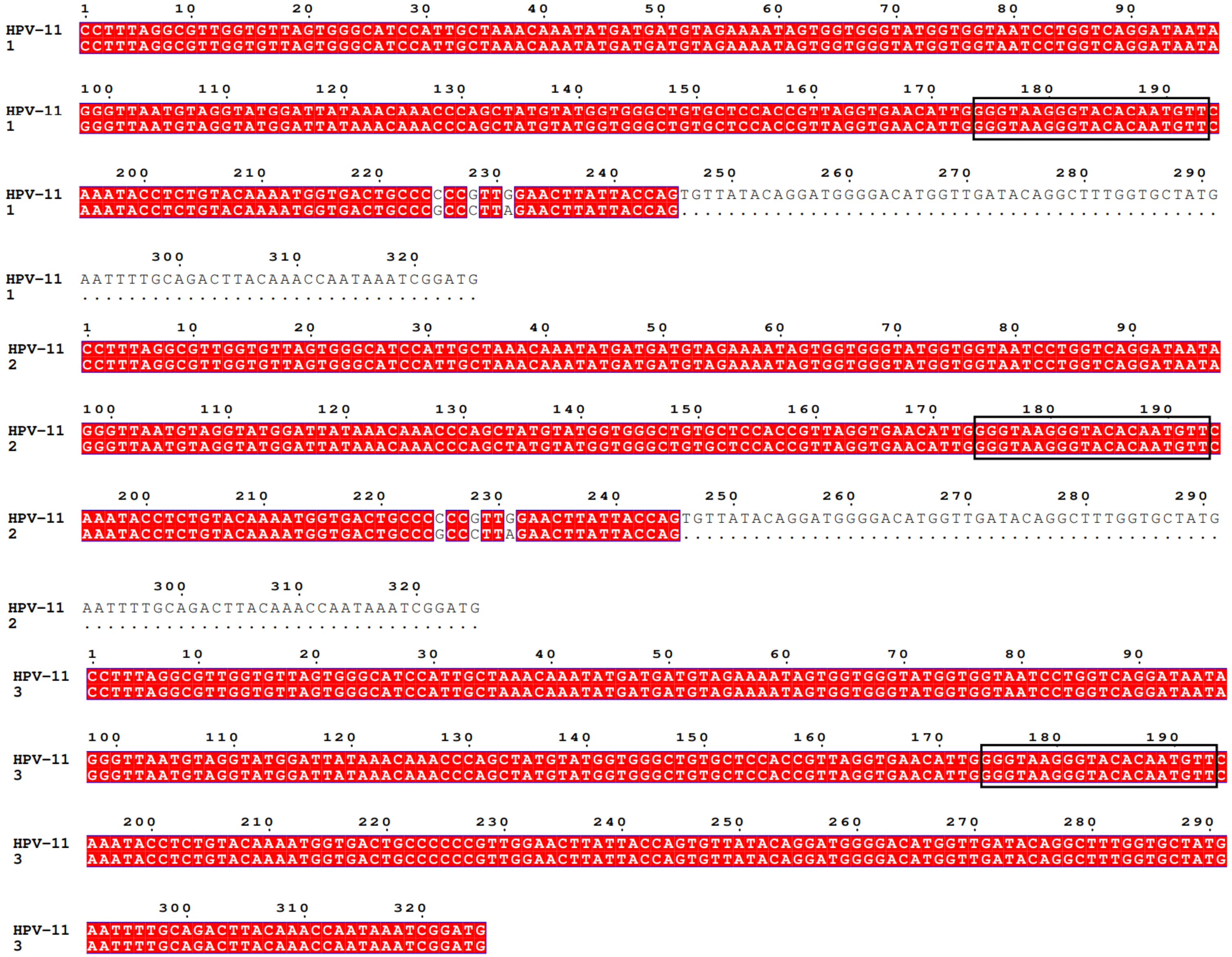
HPV-11 1
CCTTTAGGCGTTGGTGTTAGTGGGCATCCATTGCTAAACAAATATGATGATGTAGAAAATAGTGGTGGGTATGGTGGTAATCCTGGTCAGGATAATA
CCTTTAGGCGTTGGTGTTAGTGGGCATCCATTGCTAAACAAATATGATGATGTAGAAAATAGTGGTGGGTATGGTGGTAATCCTGGTCAGGATAATA
GGGTTAATGTAGGTATGGATTATAAACAAACCCAGCTATGTATGGTGGGCTGTGCTCCACCGTTAGGTGAACATTGGGGTAAGGGTACACAATGTTC
GGGTTAATGTAGGTATGGATTATAAACAAACCCAGCTATGTATGGTGGGCTGTGCTCCACCGTTAGGTGAACATTGGGGTAAGGGTACACAATGTTC
AAATACCTCTGTACAAAATGGTGACTGCCCCCCGTTGGAACTTATTACCAGTGTTATACAGGATGGGGACATGGTTGATACAGGCTTTGGTGCTATG
AAATACCTCTGTACAAAATGGTGACTGCCCGCCCTTAGAACTTATTACCAG
AATTTTGCAGACTTACAAACCAATAAATCGGATG
HPV-11 2
CCTTTAGGCGTTGGTGTTAGTGGGCATCCATTGCTAAACAAATATGATGATGTAGAAAATAGTGGTGGGTATGGTGGTAATCCTGGTCAGGATAATA
CCTTTAGGCGTTGGTGTTAGTGGGCATCCATTGCTAAACAAATATGATGATGTAGAAAATAGTGGTGGGTATGGTGGTAATCCTGGTCAGGATAATA
GGGTTAATGTAGGTATGGATTATAAACAAACCCAGCTATGTATGGTGGGCTGTGCTCCACCGTTAGGTGAACATTGGGGTAAGGGTACACAATGTTC
GGGTTAATGTAGGTATGGATTATAAACAAACCCAGCTATGTATGGTGGGCTGTGCTCCACCGTTAGGTGAACATTGGGGTAAGGGTACACAATGTTC
AAATACCTCTGTACAAAATGGTGACTGCCCCCCGTTGGAACTTATTACCAGTGTTATACAGGATGGGGACATGGTTGATACAGGCTTTGGTGCTATG
AAATACCTCTGTACAAAATGGTGACTGCCCGCCCTTAGAACTTATTACCAG
AATTTTGCAGACTTACAAACCAATAAATCGGATG
HPV-11 3
CCTTTAGGCGTTGGTGTTAGTGGGCATCCATTGCTAAACAAATATGATGATGTAGAAAATAGTGGTGGGTATGGTGGTAATCCTGGTCAGGATAATA
CCTTTAGGCGTTGGTGTTAGTGGGCATCCATTGCTAAACAAATATGATGATGTAGAAAATAGTGGTGGGTATGGTGGTAATCCTGGTCAGGATAATA
GGGTTAATGTAGGTATGGATTATAAACAAACCCAGCTATGTATGGTGGGCTGTGCTCCACCGTTAGGTGAACATTGGGGTAAGGGTACACAATGTTC
GGGTTAATGTAGGTATGGATTATAAACAAACCCAGCTATGTATGGTGGGCTGTGCTCCACCGTTAGGTGAACATTGGGGTAAGGGTACACAATGTTC
AAATACCTCTGTACAAAATGGTGACTGCCCCCCGTTGGAACTTATTACCAGTGTTATACAGGATGGGGACATGGTTGATACAGGCTTTGGTGCTATG
AAATACCTCTGTACAAAATGGTGACTGCCCCCCGTTGGAACTTATTACCAGTGTTATACAGGATGGGGACATGGTTGATACAGGCTTTGGTGCTATG
AATTTTGCAGACTTACAAACCAATAAATCGGATG
AATTTTGCAGACTTACAAACCAATAAATCGGATG

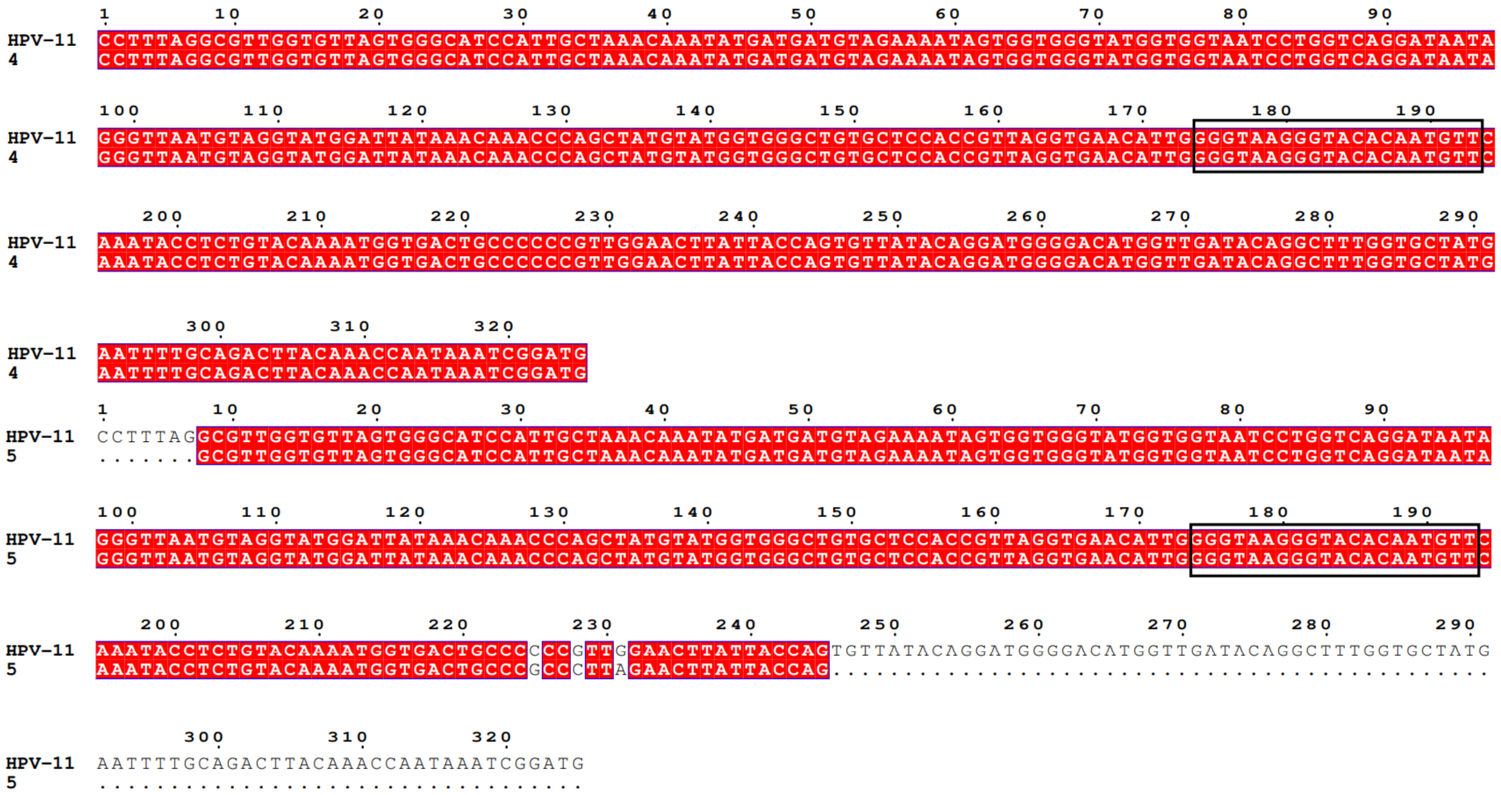
1 10 20 30 40 50 60 70 80 90
HPV-11 CCTTTAGGCGTTGGTGTTAGTGGGCATCCATTGCTAAACAAATATGATGATGTAGAAAATAGTGGTGGGTATGGTGGTAATCCTGGTCAGGATAATA
4 CCTTTAGGCGTTGGTGTTAGTGGGCATCCATTGCTAAACAAATATGATGATGTAGAAAATAGTGGTGGGTATGGTGGTAATCCTGGTCAGGATAATA
100 110 120 130 140 150 160 170 180 190
HPV-11 GGGTTAATGTAGGTATGGATTATAAACAAACCCAGCTATGTATGGTGGGCTGTGCTCCACCGTTAGGTGAACATTGGGGTAAGGGTACACAATGTTC
4 GGGTTAATGTAGGTATGGATTATAAACAAACCCAGCTATGTATGGTGGGCTGTGCTCCACCGTTAGGTGAACATTGGGGTAAGGGTACACAATGTTC
200 210 220 230 240 250 260 270 280 290
HPV-11 AAATACCTCTGTACAAAATGGTGACTGCCCCCCGTTGGAACTTATTACCAGTGTTATACAGGATGGGGACATGGTTGATACAGGCTTTGGTGCTATG
4 AAATACCTCTGTACAAAATGGTGACTGCCCCCCGTTGGAACTTATTACCAGTGTTATACAGGATGGGGACATGGTTGATACAGGCTTTGGTGCTATG
300 310 320
HPV-11 AATTTTGCAGACTTACAAACCAATAAATCGGATG
4 AATTTTGCAGACTTACAAACCAATAAATCGGATG
1 10 20 30 40 50 60 70 80 90
HPV-11 CCTTTAGGCGTTGGTGTTAGTGGGCATCCATTGCTAAACAAATATGATGATGTAGAAAATAGTGGTGGGTATGGTGGTAATCCTGGTCAGGATAATA
5 .......GCGTTGGTGTTAGTGGGCATCCATTGCTAAACAAATATGATGATGTAGAAAATAGTGGTGGGTATGGTGGTAATCCTGGTCAGGATAATA
100 110 120 130 140 150 160 170 180 190
HPV-11 GGGTTAATGTAGGTATGGATTATAAACAAACCCAGCTATGTATGGTGGGCTGTGCTCCACCGTTAGGTGAACATTGGGGTAAGGGTACACAATGTTC
5 GGGTTAATGTAGGTATGGATTATAAACAAACCCAGCTATGTATGGTGGGCTGTGCTCCACCGTTAGGTGAACATTGGGGTAAGGGTACACAATGTTC
200 210 220 230 240 250 260 270 280 290
HPV-11 AAATACCTCTGTACAAAATGGTGACTGCCCCCCGTTGGAACTTATTACCAGTGTTATACAGGATGGGGACATGGTTGATACAGGCTTTGGTGCTATG
5 AAATACCTCTGTACAAAATGGTGACTGCCCGCCCTTAGAACTTATTACCAG...
300 310 320
HPV-11 AATTTTGCAGACTTACAAACCAATAAATCGGATG
5 ....

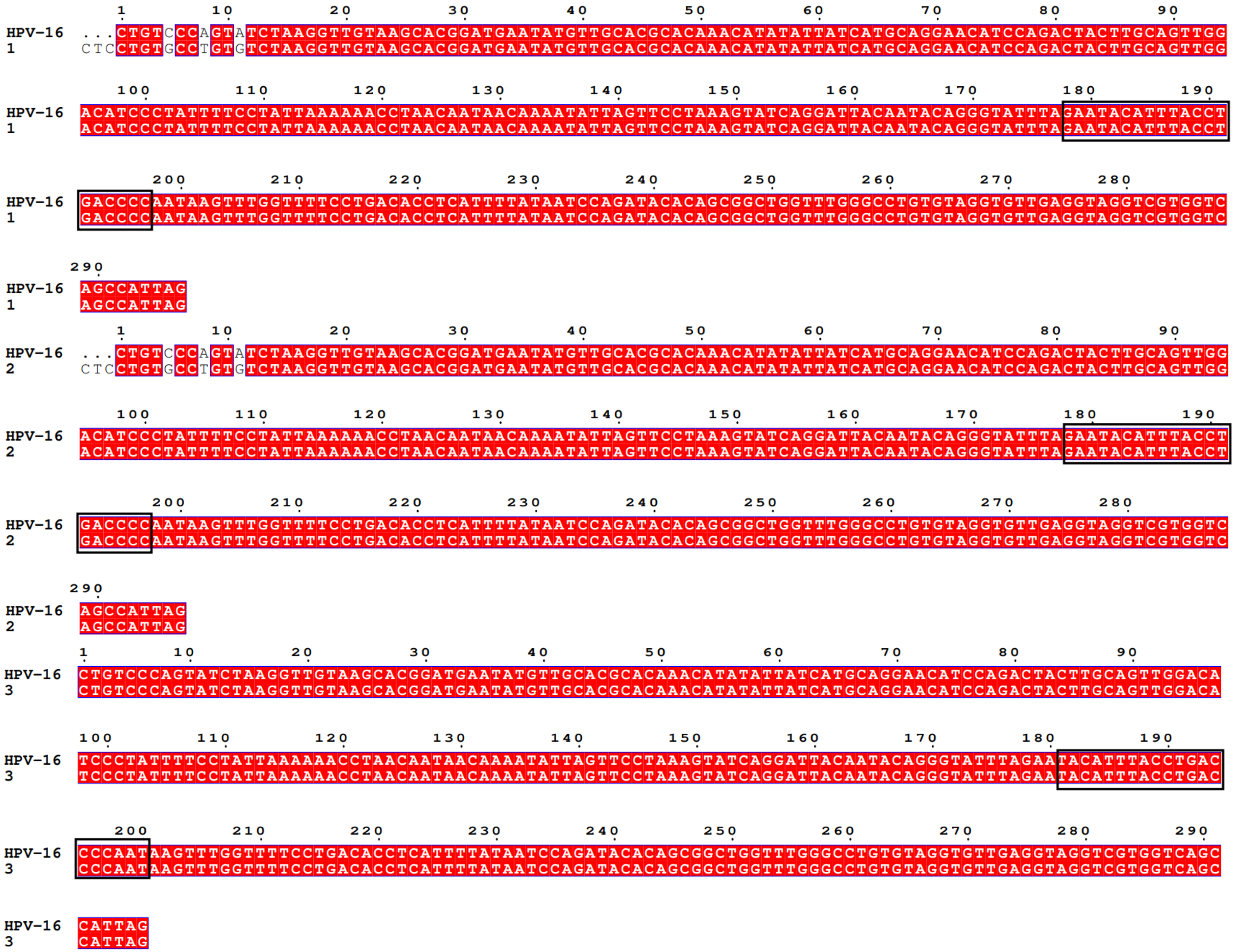

HPV-16 ...CTGTCCCAGTATCTAAGGTTGTAAGCACGGATGAATATGTTGCACGCACAAACATATATTATCATGCAGGAACATCCAGACTACTTGCAGTTGG
1 CTCCTGTGCCTGTGTCTAAGGTTGTAAGCACGGATGAATATGTTGCACGCACAAACATATATTATCATGCAGGAACATCCAGACTACTTGCAGTTGG
HPV-16 ACATCCCTATTTTCCTATTAAAAAACCTAACAATAACAAAATATTAGTTCCTAAAGTATCAGGATTACAATACAGGGTATTTAGAATACATTTACCT
1 ACATCCCTATTTTCCTATTAAAAAACCTAACAATAACAAAATATTAGTTCCTAAAGTATCAGGATTACAATACAGGGTATTTAGAATACATTTACCT
HPV-16 GACCCCAATAAGTTTGGTTTTCCTGACACCTCATTTTATAATCCAGATACACAGCGGCTGGTTTGGGCCTGTGTAGGTGTTGAGGTAGGTCGTGGTC
1 GACCCCAATAAGTTTGGTTTTCCTGACACCTCATTTTATAATCCAGATACACAGCGGCTGGTTTGGGCCTGTGTAGGTGTTGAGGTAGGTCGTGGTC
HPV-16 AGCCATTAG
1 AGCCATTAG
HPV-16 ...CTGTCCCAGTATCTAAGGTTGTAAGCACGGATGAATATGTTGCACGCACAAACATATATTATCATGCAGGAACATCCAGACTACTTGCAGTTGG
2 CTCCTGTGCCTGTGTCTAAGGTTGTAAGCACGGATGAATATGTTGCACGCACAAACATATATTATCATGCAGGAACATCCAGACTACTTGCAGTTGG
HPV-16 ACATCCCTATTTTCCTATTAAAAAACCTAACAATAACAAAATATTAGTTCCTAAAGTATCAGGATTACAATACAGGGTATTTAGAATACATTTACCT
2 ACATCCCTATTTTCCTATTAAAAAACCTAACAATAACAAAATATTAGTTCCTAAAGTATCAGGATTACAATACAGGGTATTTAGAATACATTTACCT
HPV-16 GACCCCAATAAGTTTGGTTTTCCTGACACCTCATTTTATAATCCAGATACACAGCGGCTGGTTTGGGCCTGTGTAGGTGTTGAGGTAGGTCGTGGTC
2 GACCCCAATAAGTTTGGTTTTCCTGACACCTCATTTTATAATCCAGATACACAGCGGCTGGTTTGGGCCTGTGTAGGTGTTGAGGTAGGTCGTGGTC
HPV-16 AGCCATTAG
2 AGCCATTAG
HPV-16 CTGTCCCAGTATCTAAGGTTGTAAGCACGGATGAATATGTTGCACGCACAAACATATATTATCATGCAGGAACATCCAGACTACTTGCAGTTGGACA
3 CTGTCCCAGTATCTAAGGTTGTAAGCACGGATGAATATGTTGCACGCACAAACATATATTATCATGCAGGAACATCCAGACTACTTGCAGTTGGACA
HPV-16 TCCCTATTTTCCTATTAAAAAACCTAACAATAACAAAATATTAGTTCCTAAAGTATCAGGATTACAATACAGGGTATTTAGAATACATTTACCTGAC
3 TCCCTATTTTCCTATTAAAAAACCTAACAATAACAAAATATTAGTTCCTAAAGTATCAGGATTACAATACAGGGTATTTAGAATACATTTACCTGAC
HPV-16 CCCAATAAGTTTGGTTTTCCTGACACCTCATTTTATAATCCAGATACACAGCGGCTGGTTTGGGCCTGTGTAGGTGTTGAGGTAGGTCGTGGTCAGC
3 CCCAATAAGTTTGGTTTTCCTGACACCTCATTTTATAATCCAGATACACAGCGGCTGGTTTGGGCCTGTGTAGGTGTTGAGGTAGGTCGTGGTCAGC
HPV-16 CATTAG
3 CATTAG

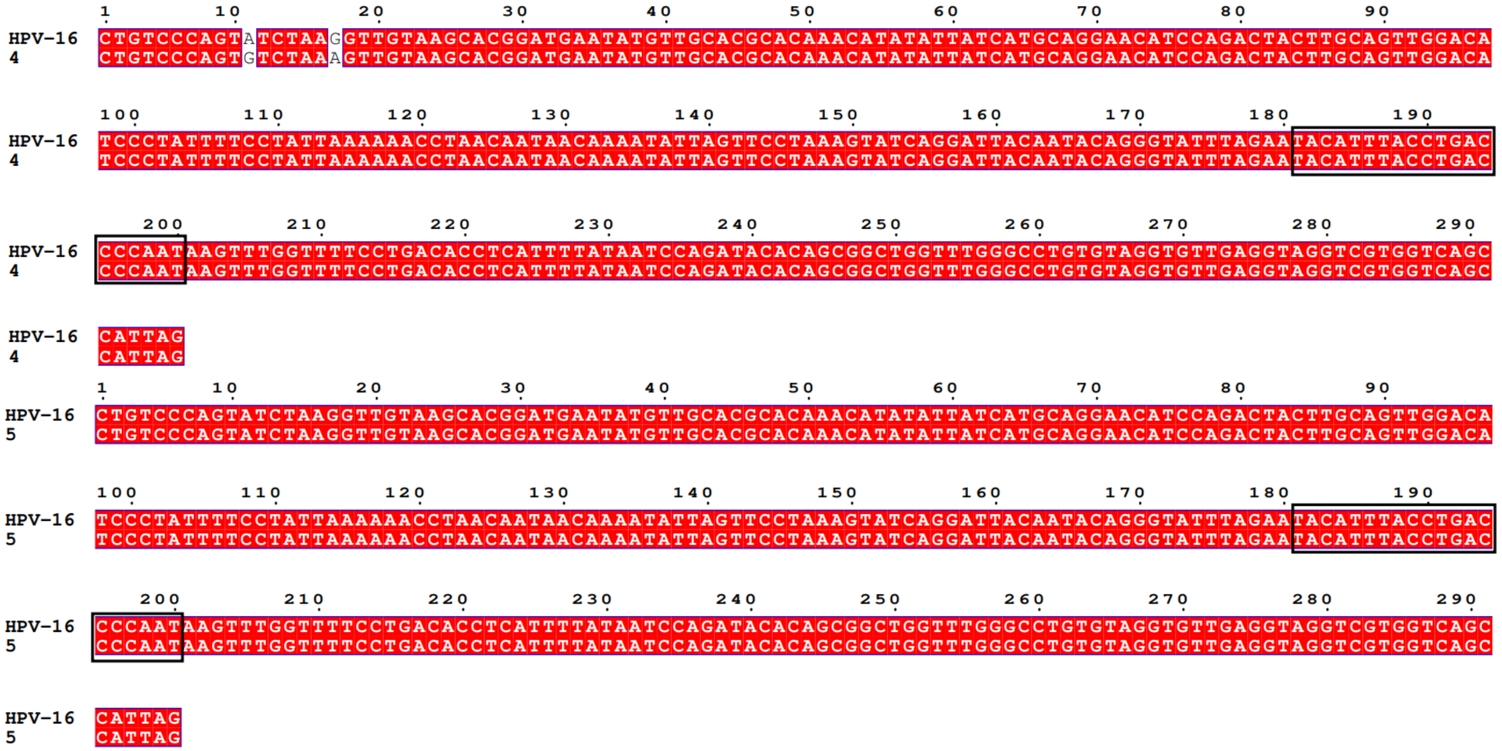

1 10 20 30 40 50 60 70 80 90
HPV-16 CTGTCCCAGTATCTAAGGTTGTAAGCACGGATGAATATGTTGCACGCACAAACATATATTATCATGCAGGAACATCCAGACTACTTGCAGTTGGACA
4 CTGTCCCAGTGTCTAAAGTTGTAAGCACGGATGAATATGTTGCACGCACAAACATATATTATCATGCAGGAACATCCAGACTACTTGCAGTTGGACA
100 110 120 130 140 150 160 170 180 190
HPV-16 TCCCTATTTTCCTATTAAAAAAACCTAACAATAACAAAATATTAGTTCCTAAAGTATCAGGATTACAATACAGGGTATTTAGAATACATTTACCTGAC
4 TCCCTATTTTCCTATTAAAAAAACCTAACAATAACAAAATATTAGTTCCTAAAGTATCAGGATTACAATACAGGGTATTTAGAATACATTTACCTGAC
200 210 220 230 240 250 260 270 280 290
HPV-16 CCCAATAAGTTTGGTTTTCCTGACACCTCATTTTATAATCCAGATACACAGCGGCTGGTTTGGGCCTGTGTAGGTGTTGAGGTAGGTCGTGGTCAGC
4 CCCAATAAGTTTGGTTTTCCTGACACCTCATTTTATAATCCAGATACACAGCGGCTGGTTTGGGCCTGTGTAGGTGTTGAGGTAGGTCGTGGTCAGC
HPV-16 CATTAG
4 CATTAG
1 10 20 30 40 50 60 70 80 90
HPV-16 CTGTCCCAGTATCTAAGGTTGTAAGCACGGATGAATATGTTGCACGCACAAACATATATTATCATGCAGGAACATCCAGACTACTTGCAGTTGGACA
5 CTGTCCCAGTATCTAAGGTTGTAAGCACGGATGAATATGTTGCACGCACAAACATATATTATCATGCAGGAACATCCAGACTACTTGCAGTTGGACA
100 110 120 130 140 150 160 170 180 190
HPV-16 TCCCTATTTTCCTATTAAAAAAACCTAACAATAACAAAATATTAGTTCCTAAAGTATCAGGATTACAATACAGGGTATTTAGAATACATTTACCTGAC
5 TCCCTATTTTCCTATTAAAAAAACCTAACAATAACAAAATATTAGTTCCTAAAGTATCAGGATTACAATACAGGGTATTTAGAATACATTTACCTGAC
200 210 220 230 240 250 260 270 280 290
HPV-16 CCCAATAAGTTTGGTTTTCCTGACACCTCATTTTATAATCCAGATACACAGCGGCTGGTTTGGGCCTGTGTAGGTGTTGAGGTAGGTCGTGGTCAGC
5 CCCAATAAGTTTGGTTTTCCTGACACCTCATTTTATAATCCAGATACACAGCGGCTGGTTTGGGCCTGTGTAGGTGTTGAGGTAGGTCGTGGTCAGC
HPV-16 CATTAG
5 CATTAG

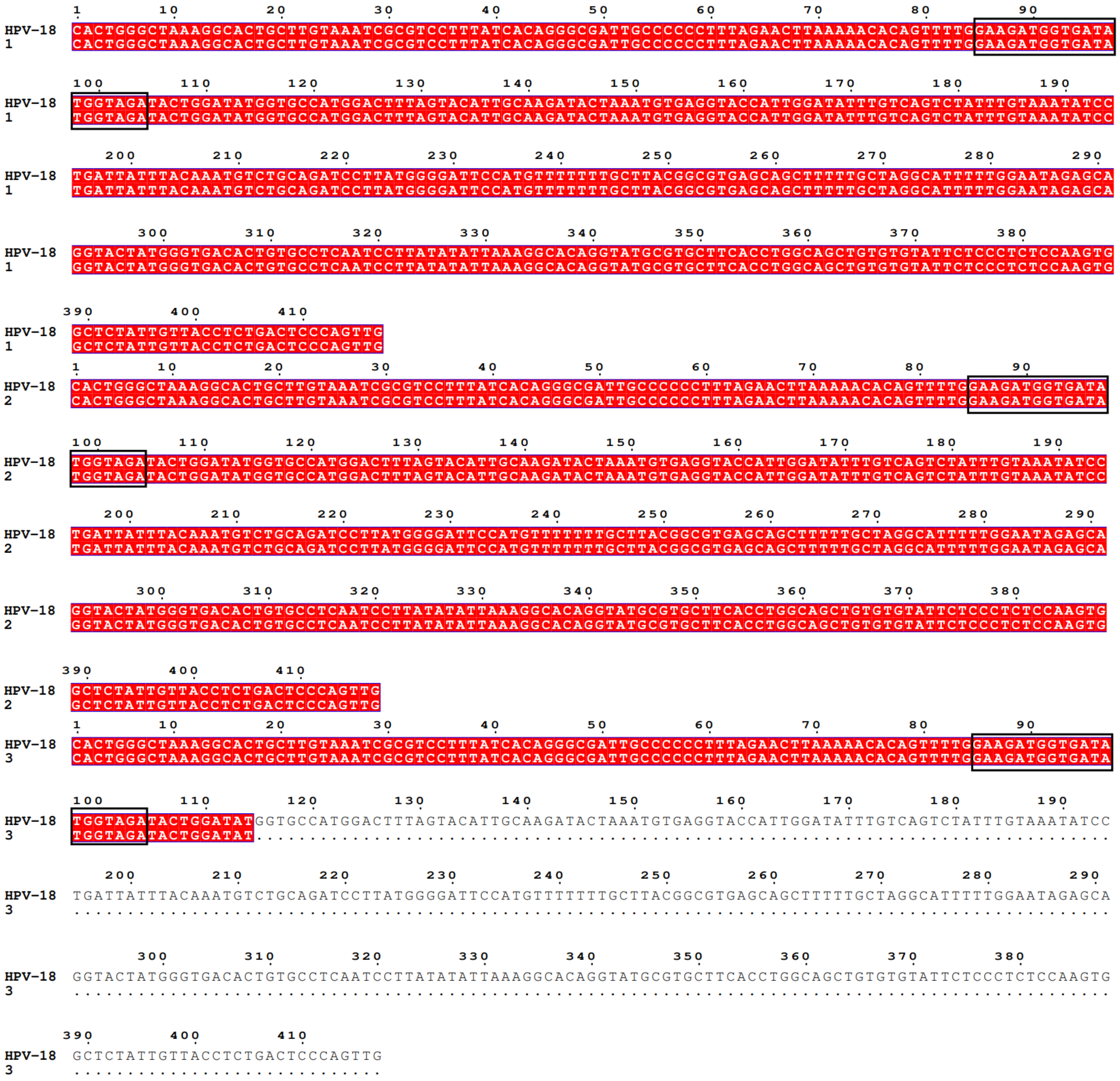

1 10 20 30 40 50 60 70 80 90
HPV-18 CACTGGGCTAAAGGCACTGCTTGTAAATCGCGTCCTTTATCACAGGGCGATTGCCCCCCTTTAGAACTTAAAAACACAGTTTTGGAAGATGGTGATA
1 CACTGGGCTAAAGGCACTGCTTGTAAATCGCGTCCTTTATCACAGGGCGATTGCCCCCCTTTAGAACTTAAAAACACAGTTTTGGAAGATGGTGATA
100 110 120 130 140 150 160 170 180 190
HPV-18 TGGTAGATACTGGATATGGTGCCATGGACTTTAGTACATTGCAAGATACTAAATGTGAGGTACCATTGGATATTTGTCAGTCTATTTGTAAATATCC
1 TGGTAGATACTGGATATGGTGCCATGGACTTTAGTACATTGCAAGATACTAAATGTGAGGTACCATTGGATATTTGTCAGTCTATTTGTAAATATCC
200 210 220 230 240 250 260 270 280 290
HPV-18 TGATTATTTACAAATGTCTGCAGATCCTTATGGGGATTCCATGTTTTTTTGCTTACGGCGTGAGCAGCTTTTTGCTAGGCATTTTTGGAATAGAGCA
1 TGATTATTTACAAATGTCTGCAGATCCTTATGGGGATTCCATGTTTTTTTGCTTACGGCGTGAGCAGCTTTTTGCTAGGCATTTTTGGAATAGAGCA
300 310 320 330 340 350 360 370 380
HPV-18 GGTACTATGGGTGACACTGTGCCTCAATCCTTATATATTAAAGGCACAGGTATGCGTGCTTCACCTGGCAGCTGTGTGTATTCTCCCTCTCCAAGTG
1 GGTACTATGGGTGACACTGTGCCTCAATCCTTATATATTAAAGGCACAGGTATGCGTGCTTCACCTGGCAGCTGTGTGTATTCTCCCTCTCCAAGTG
390 400 410
HPV-18 GCTCTATTGTTACCTCTGACTCCCAGTTG
1 GCTCTATTGTTACCTCTGACTCCCAGTTG
1 10 20 30 40 50 60 70 80 90
HPV-18 CACTGGGCTAAAGGCACTGCTTGTAAATCGCGTCCTTTATCACAGGGCGATTGCCCCCCTTTAGAACTTAAAAACACAGTTTTGGAAGATGGTGATA
2 CACTGGGCTAAAGGCACTGCTTGTAAATCGCGTCCTTTATCACAGGGCGATTGCCCCCCTTTAGAACTTAAAAACACAGTTTTGGAAGATGGTGATA
100 110 120 130 140 150 160 170 180 190
HPV-18 TGGTAGATACTGGATATGGTGCCATGGACTTTAGTACATTGCAAGATACTAAATGTGAGGTACCATTGGATATTTGTCAGTCTATTTGTAAATATCC
2 TGGTAGATACTGGATATGGTGCCATGGACTTTAGTACATTGCAAGATACTAAATGTGAGGTACCATTGGATATTTGTCAGTCTATTTGTAAATATCC
200 210 220 230 240 250 260 270 280 290
HPV-18 TGATTATTTACAAATGTCTGCAGATCCTTATGGGGATTCCATGTTTTTTTGCTTACGGCGTGAGCAGCTTTTTGCTAGGCATTTTTGGAATAGAGCA
2 TGATTATTTACAAATGTCTGCAGATCCTTATGGGGATTCCATGTTTTTTTGCTTACGGCGTGAGCAGCTTTTTGCTAGGCATTTTTGGAATAGAGCA
300 310 320 330 340 350 360 370 380
HPV-18 GGTACTATGGGTGACACTGTGCCTCAATCCTTATATATTAAAGGCACAGGTATGCGTGCTTCACCTGGCAGCTGTGTGTATTCTCCCTCTCCAAGTG
2 GGTACTATGGGTGACACTGTGCCTCAATCCTTATATATTAAAGGCACAGGTATGCGTGCTTCACCTGGCAGCTGTGTGTATTCTCCCTCTCCAAGTG
390 400 410
HPV-18 GCTCTATTGTTACCTCTGACTCCCAGTTG
2 GCTCTATTGTTACCTCTGACTCCCAGTTG
1 10 20 30 40 50 60 70 80 90
HPV-18 CACTGGGCTAAAGGCACTGCTTGTAAATCGCGTCCTTTATCACAGGGCGATTGCCCCCCTTTAGAACTTAAAAACACAGTTTTGGAAGATGGTGATA
3 CACTGGGCTAAAGGCACTGCTTGTAAATCGCGTCCTTTATCACAGGGCGATTGCCCCCCTTTAGAACTTAAAAACACAGTTTTGGAAGATGGTGATA
100 110 120 130 140 150 160 170 180 190
HPV-18 TGGTAGATACTGGATATGGTGCCATGGACTTTAGTACATTGCAAGATACTAAATGTGAGGTACCATTGGATATTTGTCAGTCTATTTGTAAATATCC
3 TGGTAGATACTGGATAT..
200 210 220 230 240 250 260 270 280 290
HPV-18 TGATTATTTACAAATGTCTGCAGATCCTTATGGGGATTCCATGTTTTTTTGCTTACGGCGTGAGCAGCTTTTTGCTAGGCATTTTTGGAATAGAGCA
3 ....
300 310 320 330 340 350 360 370 380
HPV-18 GGTACTATGGGTGACACTGTGCCTCAATCCTTATATATTAAAGGCACAGGTATGCGTGCTTCACCTGGCAGCTGTGTGTATTCTCCCTCTCCAAGTG
3 ....
390 400 410
HPV-18 GCTCTATTGTTACCTCTGACTCCCAGTTG
3 ....

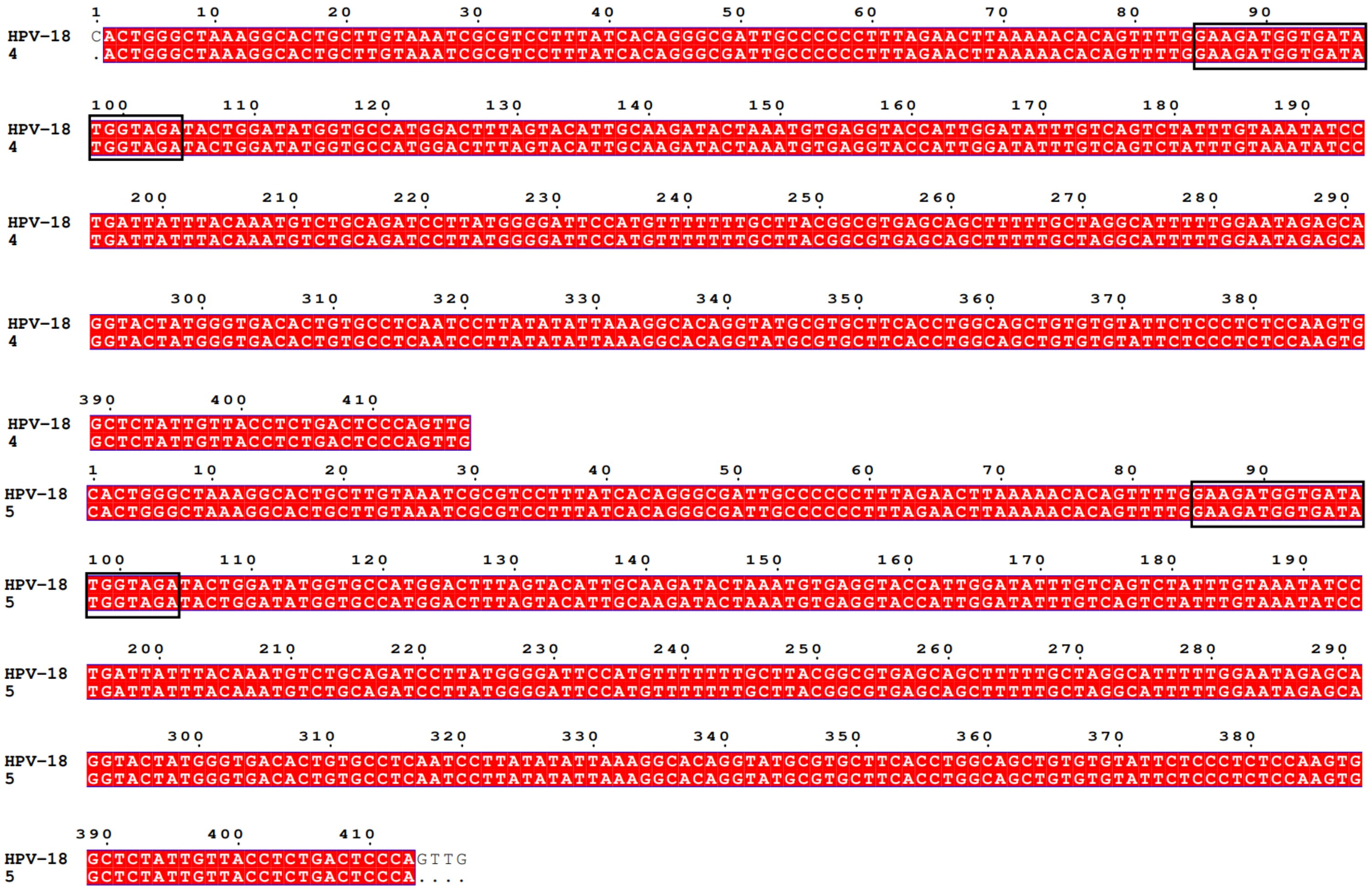

1 10 20 30 40 50 60 70 80 90
HPV-18 CACTGGGCTAAAGGCACTGCTTGTAAATCGCGTCCTTTATCACAGGGCGATTGCCCCCCTTTAGAACTTAAAAACACAGTTTTGGAAGATGGTGATA
4 .ACTGGGCTAAAGGCACTGCTTGTAAATCGCGTCCTTTATCACAGGGCGATTGCCCCCCTTTAGAACTTAAAAACACAGTTTTGGAAGATGGTGATA
100 110 120 130 140 150 160 170 180 190
HPV-18 TGGTAGATACTGGATATGGTGCCATGGACTTTAGTACATTGCAAGATACTAAATGTGAGGTACCATTGGATATTTGTCAGTCTATTTGTAAATATCC
4 TGGTAGATACTGGATATGGTGCCATGGACTTTAGTACATTGCAAGATACTAAATGTGAGGTACCATTGGATATTTGTCAGTCTATTTGTAAATATCC
200 210 220 230 240 250 260 270 280 290
HPV-18 TGATTATTTACAAATGTCTGCAGATCCTTATGGGGATTCCATGTTTTTTTGCTTACGGCGTGAGCAGCTTTTTGCTAGGCATTTTTGGAATAGAGCA
4 TGATTATTTACAAATGTCTGCAGATCCTTATGGGGATTCCATGTTTTTTTGCTTACGGCGTGAGCAGCTTTTTGCTAGGCATTTTTGGAATAGAGCA
300 310 320 330 340 350 360 370 380
HPV-18 GGTACTATGGGTGACACTGTGCCTCAATCCTTATATATTAAAGGCACAGGTATGCGTGCTTCACCTGGCAGCTGTGTGTATTCTCCCTCTCCAAGTG
4 GGTACTATGGGTGACACTGTGCCTCAATCCTTATATATTAAAGGCACAGGTATGCGTGCTTCACCTGGCAGCTGTGTGTATTCTCCCTCTCCAAGTG
390 400 410
HPV-18 GCTCTATTGTTACCTCTGACTCCCAGTTG
4 GCTCTATTGTTACCTCTGACTCCCAGTTG
1 10 20 30 40 50 60 70 80 90
HPV-18 CACTGGGCTAAAGGCACTGCTTGTAAATCGCGTCCTTTATCACAGGGCGATTGCCCCCCTTTAGAACTTAAAAACACAGTTTTGGAAGATGGTGATA
5 CACTGGGCTAAAGGCACTGCTTGTAAATCGCGTCCTTTATCACAGGGCGATTGCCCCCCTTTAGAACTTAAAAACACAGTTTTGGAAGATGGTGATA
100 110 120 130 140 150 160 170 180 190
HPV-18 TGGTAGATACTGGATATGGTGCCATGGACTTTAGTACATTGCAAGATACTAAATGTGAGGTACCATTGGATATTTGTCAGTCTATTTGTAAATATCC
5 TGGTAGATACTGGATATGGTGCCATGGACTTTAGTACATTGCAAGATACTAAATGTGAGGTACCATTGGATATTTGTCAGTCTATTTGTAAATATCC
200 210 220 230 240 250 260 270 280 290
HPV-18 TGATTATTTACAAATGTCTGCAGATCCTTATGGGGATTCCATGTTTTTTTGCTTACGGCGTGAGCAGCTTTTTGCTAGGCATTTTTGGAATAGAGCA
5 TGATTATTTACAAATGTCTGCAGATCCTTATGGGGATTCCATGTTTTTTTGCTTACGGCGTGAGCAGCTTTTTGCTAGGCATTTTTGGAATAGAGCA
300 310 320 330 340 350 360 370 380
HPV-18 GGTACTATGGGTGACACTGTGCCTCAATCCTTATATATTAAAGGCACAGGTATGCGTGCTTCACCTGGCAGCTGTGTGTATTCTCCCTCTCCAAGTG
5 GGTACTATGGGTGACACTGTGCCTCAATCCTTATATATTAAAGGCACAGGTATGCGTGCTTCACCTGGCAGCTGTGTGTATTCTCCCTCTCCAAGTG
390 400 410
HPV-18 GCTCTATTGTTACCTCTGACTCCCAGTTG
5 GCTCTATTGTTACCTCTGACTCCCA....

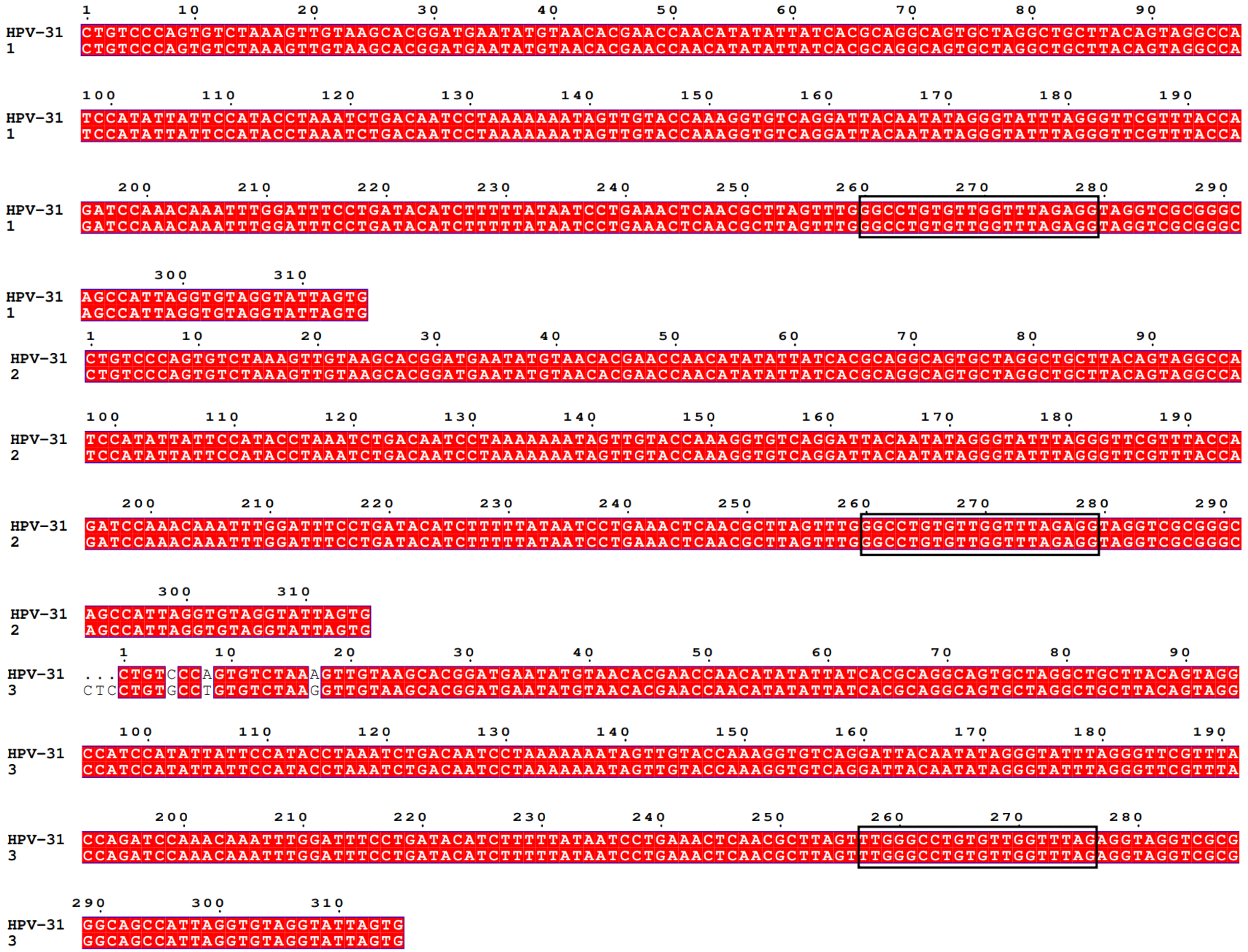
HPV-31 1
CTGTCCCAGTGTCTAAAGTTGTAAGCACGGATGAATATGTAACACGAACCAACATATATTATCACGCAGGCAGTGCTAGGCTGCTTACAGTAGGCCA
CTGTCCCAGTGTCTAAAGTTGTAAGCACGGATGAATATGTAACACGAACCAACATATATTATCACGCAGGCAGTGCTAGGCTGCTTACAGTAGGCCA
TCCATATTATTCCATACCTAAATCTGACAATCCTAAAAAAATAGTTGTACCAAAGGTGTCAGGATTACAATATAGGGTATTTAGGGTTCGTTTACCA
TCCATATTATTCCATACCTAAATCTGACAATCCTAAAAAAATAGTTGTACCAAAGGTGTCAGGATTACAATATAGGGTATTTAGGGTTCGTTTACCA
GATCCAAACAAATTTGGATTTCCTGATACATCTTTTTATAATCCTGAAACTCAACGCTTAGTTTGGGCCTGTGTTGGTTTAGAGGTAGGTCGCGGGC
GATCCAAACAAATTTGGATTTCCTGATACATCTTTTTATAATCCTGAAACTCAACGCTTAGTTTGGGCCTGTGTTGGTTTAGAGGTAGGTCGCGGGC
AGCCATTAGGTGTAGGTATTAGTG
AGCCATTAGGTGTAGGTATTAGTG
HPV-31 2
CTGTCCCAGTGTCTAAAGTTGTAAGCACGGATGAATATGTAACACGAACCAACATATATTATCACGCAGGCAGTGCTAGGCTGCTTACAGTAGGCCA
CTGTCCCAGTGTCTAAAGTTGTAAGCACGGATGAATATGTAACACGAACCAACATATATTATCACGCAGGCAGTGCTAGGCTGCTTACAGTAGGCCA
TCCATATTATTCCATACCTAAATCTGACAATCCTAAAAAAATAGTTGTACCAAAGGTGTCAGGATTACAATATAGGGTATTTAGGGTTCGTTTACCA
TCCATATTATTCCATACCTAAATCTGACAATCCTAAAAAAATAGTTGTACCAAAGGTGTCAGGATTACAATATAGGGTATTTAGGGTTCGTTTACCA
GATCCAAACAAATTTGGATTTCCTGATACATCTTTTTATAATCCTGAAACTCAACGCTTAGTTTGGGCCTGTGTTGGTTTAGAGGTAGGTCGCGGGC
GATCCAAACAAATTTGGATTTCCTGATACATCTTTTTATAATCCTGAAACTCAACGCTTAGTTTGGGCCTGTGTTGGTTTAGAGGTAGGTCGCGGGC
AGCCATTAGGTGTAGGTATTAGTG
AGCCATTAGGTGTAGGTATTAGTG
HPV-31 3
...CTGTCCCAGTGTCTAAAGTTGTAAGCACGGATGAATATGTAACACGAACCAACATATATTATCACGCAGGCAGTGCTAGGCTGCTTACAGTAGG
CTCCTGTGCCTGTGTCTAAGGTTGTAAGCACGGATGAATATGTAACACGAACCAACATATATTATCACGCAGGCAGTGCTAGGCTGCTTACAGTAGG
CCATCCATATTATTCCATACCTAAATCTGACAATCCTAAAAAAATAGTTGTACCAAAGGTGTCAGGATTACAATATAGGGTATTTAGGGTTCGTTTA
CCATCCATATTATTCCATACCTAAATCTGACAATCCTAAAAAAATAGTTGTACCAAAGGTGTCAGGATTACAATATAGGGTATTTAGGGTTCGTTTA
CCAGATCCAAACAAATTTGGATTTCCTGATACATCTTTTTATAATCCTGAAACTCAACGCTTAGTTTGGGCCTGTGTTGGTTTAGAGGTAGGTCGCG
CCAGATCCAAACAAATTTGGATTTCCTGATACATCTTTTTATAATCCTGAAACTCAACGCTTAGTTTGGGCCTGTGTTGGTTTAGAGGTAGGTCGCG
GGCAGCCATTAGGTGTAGGTATTAGTG
GGCAGCCATTAGGTGTAGGTATTAGTG

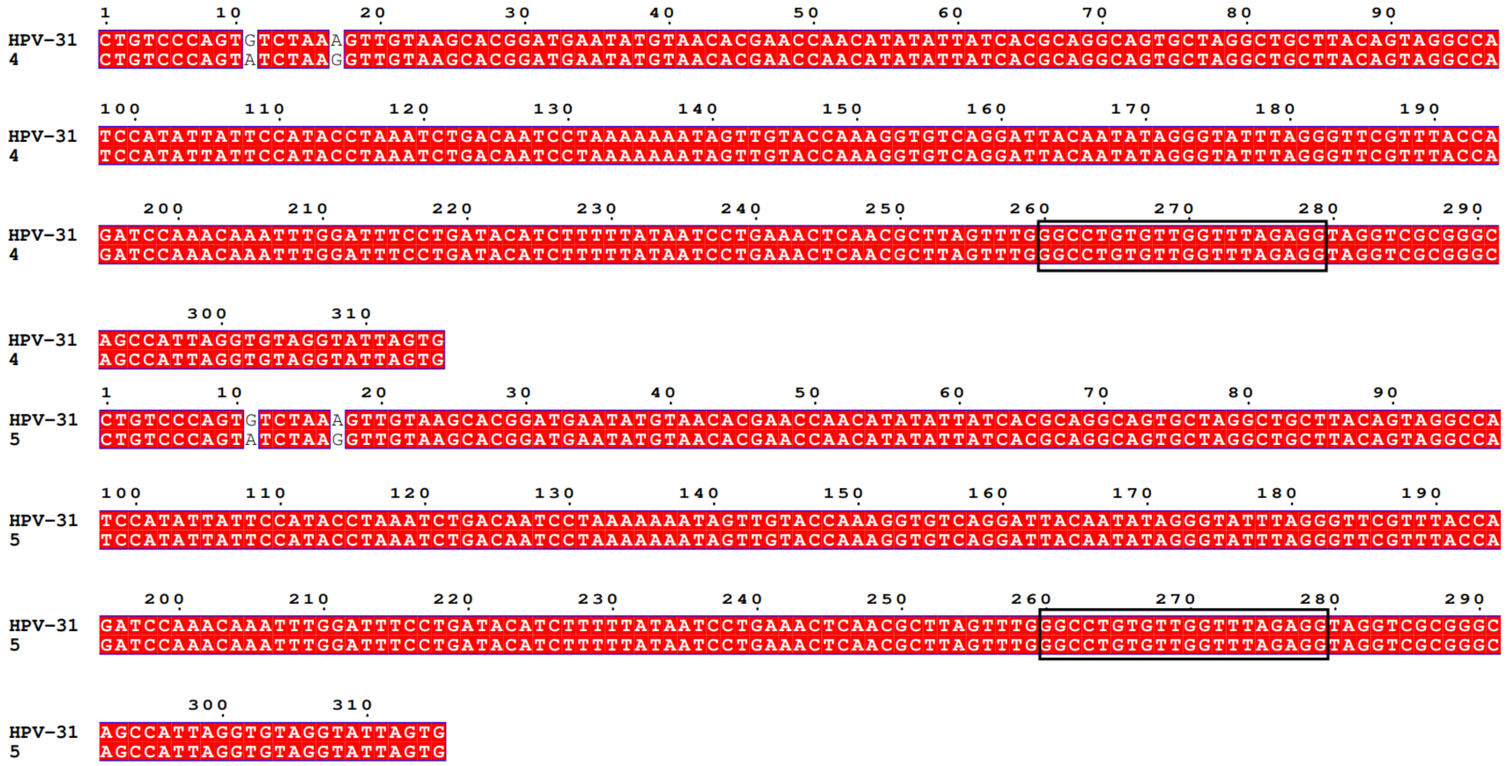
1 10 20 30 40 50 60 70 80 90
HPV-31 CTGTCCCAGTGTCTAAAGTTGTAAGCACGGATGAATATGTAACACGAACCAACATATATTATCACGCAGGCAGTGCTAGGCTGCTTACAGTAGGCCA
4 CTGTCCCAGTATCTAAGGTTGTAAGCACGGATGAATATGTAACACGAACCAACATATATTATCACGCAGGCAGTGCTAGGCTGCTTACAGTAGGCCA
100 110 120 130 140 150 160 170 180 190
HPV-31 TCCATATTATTCCATACCTAAATCTGACAATCCTAAAAAAATAGTTGTACCAAAGGTGTCAGGATTACAATATAGGGTATTTAGGGTTCGTTTACCA
4 TCCATATTATTCCATACCTAAATCTGACAATCCTAAAAAAATAGTTGTACCAAAGGTGTCAGGATTACAATATAGGGTATTTAGGGTTCGTTTACCA
200 210 220 230 240 250 260 270 280 290
HPV-31 GATCCAAACAAATTTGGATTTCCTGATACATCTTTTTATAATCCTGAAACTCAACGCTTAGTTTGGGCCTGTGTTGGTTTAGAGGTAGGTCGCGGGC
4 GATCCAAACAAATTTGGATTTCCTGATACATCTTTTTATAATCCTGAAACTCAACGCTTAGTTTGGGCCTGTGTTGGTTTAGAGGTAGGTCGCGGGC
300 310
HPV-31 AGCCATTAGGTGTAGGTATTAGTG
4 AGCCATTAGGTGTAGGTATTAGTG
1 10 20 30 40 50 60 70 80 90
HPV-31 CTGTCCCAGTGTCTAAAGTTGTAAGCACGGATGAATATGTAACACGAACCAACATATATTATCACGCAGGCAGTGCTAGGCTGCTTACAGTAGGCCA
5 CTGTCCCAGTATCTAAGGTTGTAAGCACGGATGAATATGTAACACGAACCAACATATATTATCACGCAGGCAGTGCTAGGCTGCTTACAGTAGGCCA
100 110 120 130 140 150 160 170 180 190
HPV-31 TCCATATTATTCCATACCTAAATCTGACAATCCTAAAAAAATAGTTGTACCAAAGGTGTCAGGATTACAATATAGGGTATTTAGGGTTCGTTTACCA
5 TCCATATTATTCCATACCTAAATCTGACAATCCTAAAAAAATAGTTGTACCAAAGGTGTCAGGATTACAATATAGGGTATTTAGGGTTCGTTTACCA
200 210 220 230 240 250 260 270 280 290
HPV-31 GATCCAAACAAATTTGGATTTCCTGATACATCTTTTTATAATCCTGAAACTCAACGCTTAGTTTGGGCCTGTGTTGGTTTAGAGGTAGGTCGCGGGC
5 GATCCAAACAAATTTGGATTTCCTGATACATCTTTTTATAATCCTGAAACTCAACGCTTAGTTTGGGCCTGTGTTGGTTTAGAGGTAGGTCGCGGGC
300 310
HPV-31 AGCCATTAGGTGTAGGTATTAGTG
5 AGCCATTAGGTGTAGGTATTAGTG

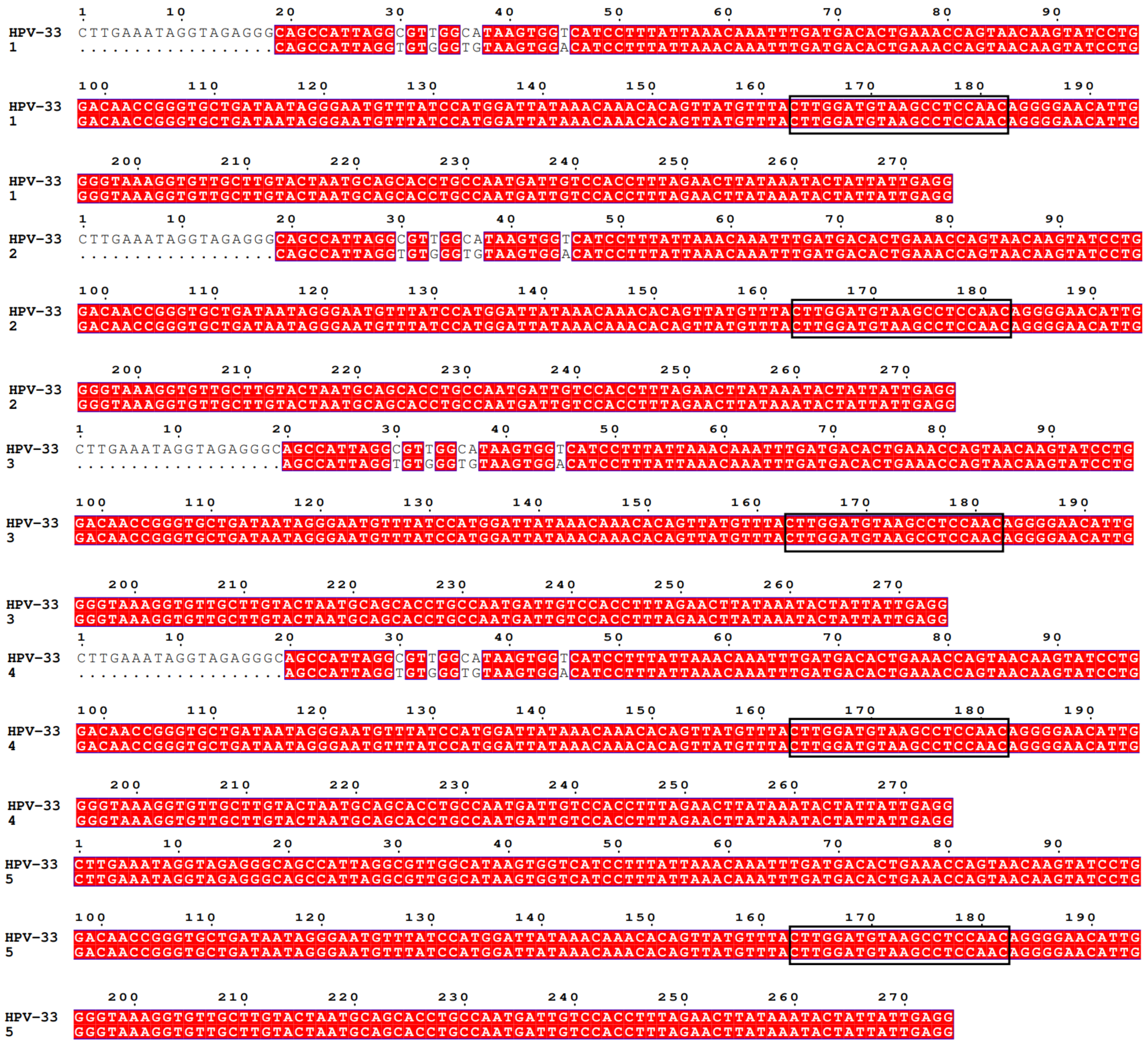

HPV-33 1
CTTGAAATAGGTAGAGGGCAGCCATTAGGCGTTGGCATAAGTGGTCATCCTTTATTAAACAAATTTGATGACACTGAAACCAGTAACAAGTATCCTG
..................CAGCCATTAGGTGTGGGTGTAAGTGGACATCCTTTATTAAACAAATTTGATGACACTGAAACCAGTAACAAGTATCCTG
HPV-33 1
GACAACCGGGTGCTGATAATAGGGAATGTTTATCCATGGATTATAAACAAACACAGTTATGTTTACTTGGATGTAAGCCTCCAACAGGGGAACATTG
GACAACCGGGTGCTGATAATAGGGAATGTTTATCCATGGATTATAAACAAACACAGTTATGTTTACTTGGATGTAAGCCTCCAACAGGGGAACATTG
HPV-33 1
GGGTAAAGGTGTTGCTTGTACTAATGCAGCACCTGCCAATGATTGTCCACCTTTAGAACTTATAAATACTATTATTGAGG
GGGTAAAGGTGTTGCTTGTACTAATGCAGCACCTGCCAATGATTGTCCACCTTTAGAACTTATAAATACTATTATTGAGG
HPV-33 2
CTTGAAATAGGTAGAGGGCAGCCATTAGGCGTTGGCATAAGTGGTCATCCTTTATTAAACAAATTTGATGACACTGAAACCAGTAACAAGTATCCTG
..................CAGCCATTAGGTGTGGGTGTAAGTGGACATCCTTTATTAAACAAATTTGATGACACTGAAACCAGTAACAAGTATCCTG
HPV-33 2
GACAACCGGGTGCTGATAATAGGGAATGTTTATCCATGGATTATAAACAAACACAGTTATGTTTACTTGGATGTAAGCCTCCAACAGGGGAACATTG
GACAACCGGGTGCTGATAATAGGGAATGTTTATCCATGGATTATAAACAAACACAGTTATGTTTACTTGGATGTAAGCCTCCAACAGGGGAACATTG
HPV-33 2
GGGTAAAGGTGTTGCTTGTACTAATGCAGCACCTGCCAATGATTGTCCACCTTTAGAACTTATAAATACTATTATTGAGG
GGGTAAAGGTGTTGCTTGTACTAATGCAGCACCTGCCAATGATTGTCCACCTTTAGAACTTATAAATACTATTATTGAGG
HPV-33 3
CTTGAAATAGGTAGAGGGCAGCCATTAGGCGTTGGCATAAGTGGTCATCCTTTATTAAACAAATTTGATGACACTGAAACCAGTAACAAGTATCCTG
...................AGCCATTAGGTGTGGGTGTAAGTGGACATCCTTTATTAAACAAATTTGATGACACTGAAACCAGTAACAAGTATCCTG
HPV-33 3
GACAACCGGGTGCTGATAATAGGGAATGTTTATCCATGGATTATAAACAAACACAGTTATGTTTACTTGGATGTAAGCCTCCAACAGGGGAACATTG
GACAACCGGGTGCTGATAATAGGGAATGTTTATCCATGGATTATAAACAAACACAGTTATGTTTACTTGGATGTAAGCCTCCAACAGGGGAACATTG
HPV-33 3
GGGTAAAGGTGTTGCTTGTACTAATGCAGCACCTGCCAATGATTGTCCACCTTTAGAACTTATAAATACTATTATTGAGG
GGGTAAAGGTGTTGCTTGTACTAATGCAGCACCTGCCAATGATTGTCCACCTTTAGAACTTATAAATACTATTATTGAGG
HPV-33 4
CTTGAAATAGGTAGAGGGCAGCCATTAGGCGTTGGCATAAGTGGTCATCCTTTATTAAACAAATTTGATGACACTGAAACCAGTAACAAGTATCCTG
...................AGCCATTAGGTGTGGGTGTAAGTGGACATCCTTTATTAAACAAATTTGATGACACTGAAACCAGTAACAAGTATCCTG
HPV-33 4
GACAACCGGGTGCTGATAATAGGGAATGTTTATCCATGGATTATAAACAAACACAGTTATGTTTACTTGGATGTAAGCCTCCAACAGGGGAACATTG
GACAACCGGGTGCTGATAATAGGGAATGTTTATCCATGGATTATAAACAAACACAGTTATGTTTACTTGGATGTAAGCCTCCAACAGGGGAACATTG
HPV-33 4
GGGTAAAGGTGTTGCTTGTACTAATGCAGCACCTGCCAATGATTGTCCACCTTTAGAACTTATAAATACTATTATTGAGG
GGGTAAAGGTGTTGCTTGTACTAATGCAGCACCTGCCAATGATTGTCCACCTTTAGAACTTATAAATACTATTATTGAGG
HPV-33 5
CTTGAAATAGGTAGAGGGCAGCCATTAGGCGTTGGCATAAGTGGTCATCCTTTATTAAACAAATTTGATGACACTGAAACCAGTAACAAGTATCCTG
CTTGAAATAGGTAGAGGGCAGCCATTAGGCGTTGGCATAAGTGGTCATCCTTTATTAAACAAATTTGATGACACTGAAACCAGTAACAAGTATCCTG
HPV-33 5
GACAACCGGGTGCTGATAATAGGGAATGTTTATCCATGGATTATAAACAAACACAGTTATGTTTACTTGGATGTAAGCCTCCAACAGGGGAACATTG
GACAACCGGGTGCTGATAATAGGGAATGTTTATCCATGGATTATAAACAAACACAGTTATGTTTACTTGGATGTAAGCCTCCAACAGGGGAACATTG
HPV-33 5
GGGTAAAGGTGTTGCTTGTACTAATGCAGCACCTGCCAATGATTGTCCACCTTTAGAACTTATAAATACTATTATTGAGG
GGGTAAAGGTGTTGCTTGTACTAATGCAGCACCTGCCAATGATTGTCCACCTTTAGAACTTATAAATACTATTATTGAGG

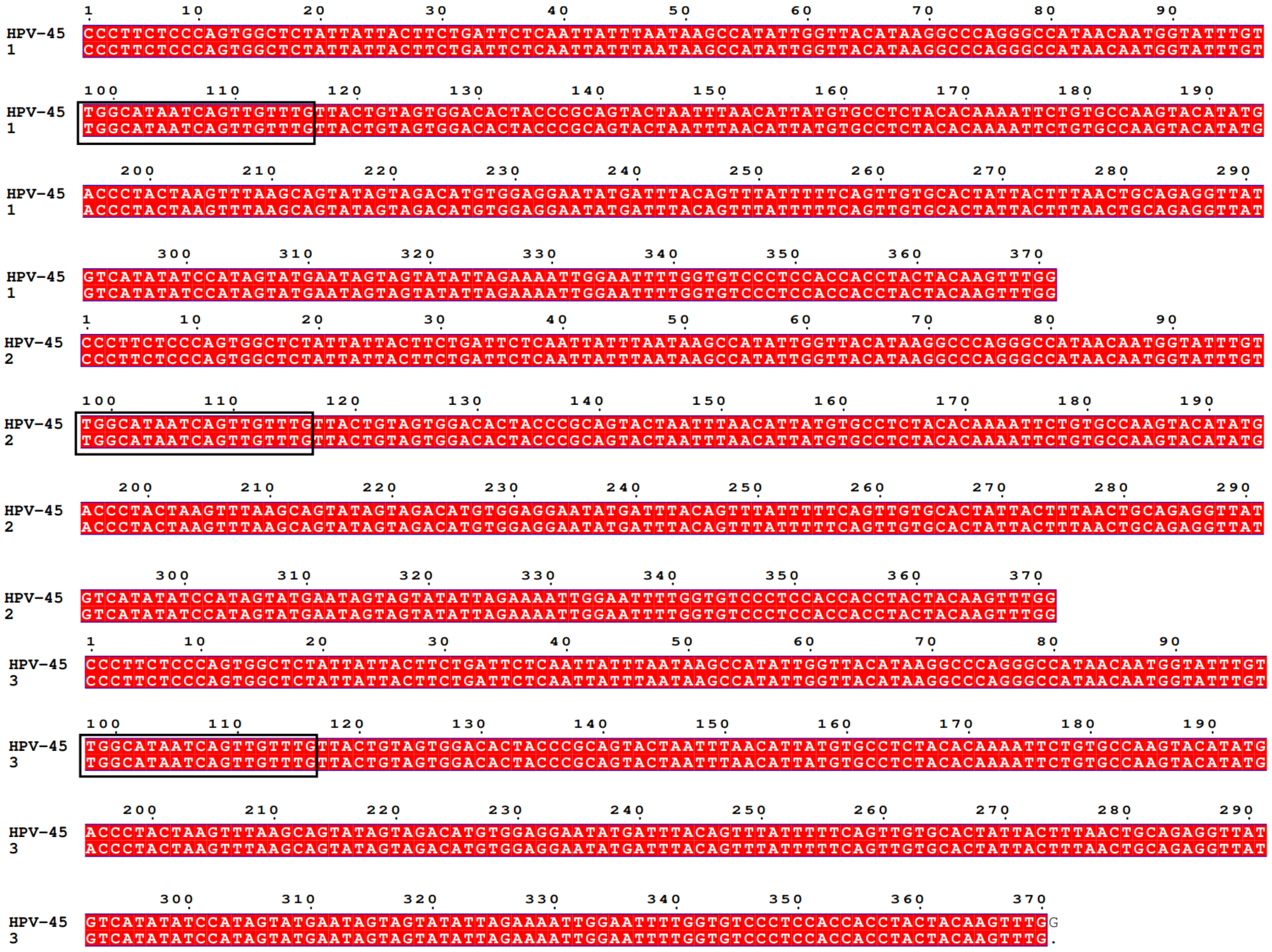
HPV-45 1
HPV-45 2
HPV-45 3

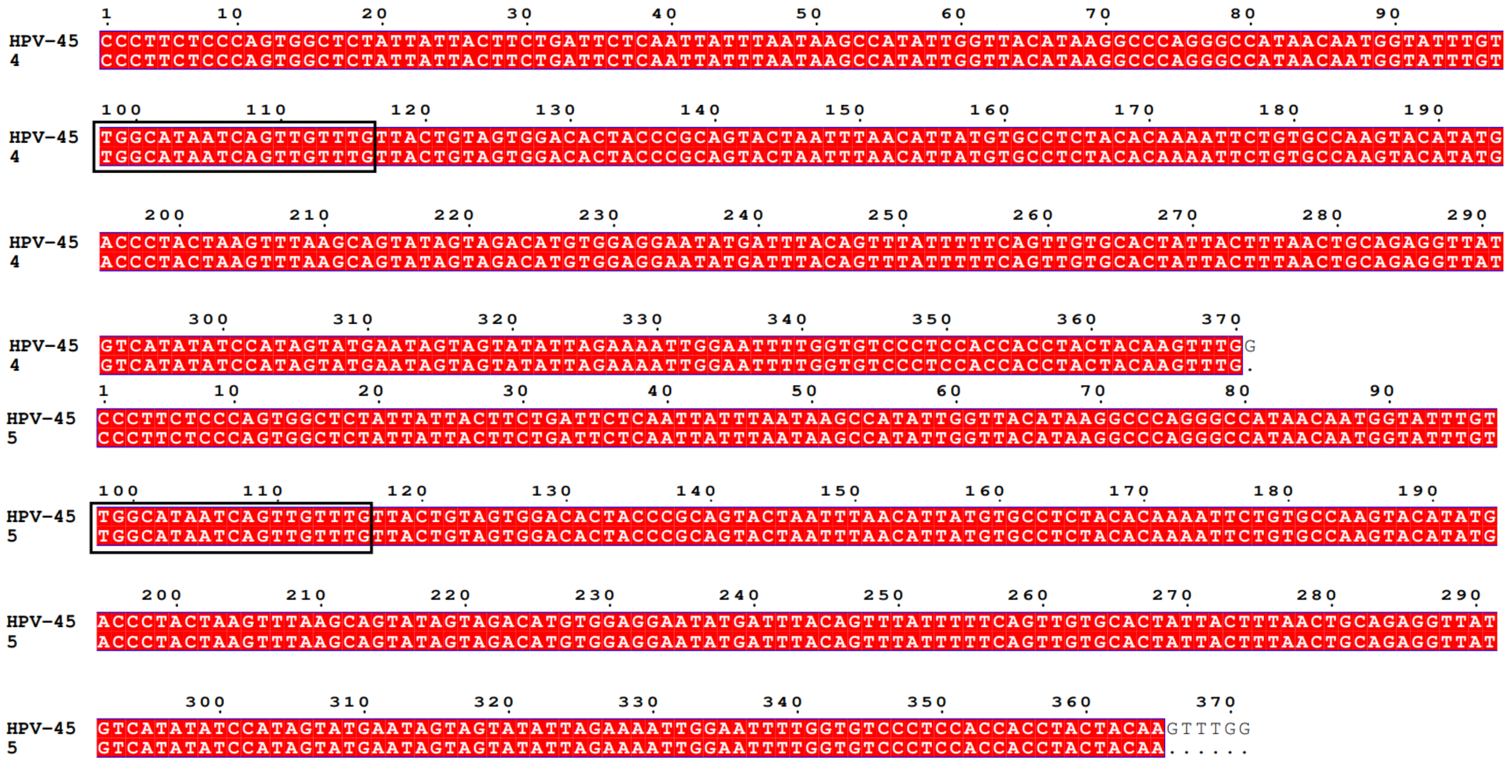
1 10 20 30 40 50 60 70 80 90
HPV-45 CCCTTCTCCCAGTGGCTCTATTATTACTTCTGATTCTCAATTATTTAATAAGCCATATTGGTTACATAAGGCCCAGGGCCATAACAATGGTATTTGT
4 CCCTTCTCCCAGTGGCTCTATTATTACTTCTGATTCTCAATTATTTAATAAGCCATATTGGTTACATAAGGCCCAGGGCCATAACAATGGTATTTGT
100 110 120 130 140 150 160 170 180 190
HPV-45 TGGCATAATCAGTTGTTTGTTACTGTAGTGGACACTACCCGCAGTACTAATTTAACATTATGTGCCTCTACACAAAATTCTGTGCCAAGTACATATG
4 TGGCATAATCAGTTGTTTGTTACTGTAGTGGACACTACCCGCAGTACTAATTTAACATTATGTGCCTCTACACAAAATTCTGTGCCAAGTACATATG
200 210 220 230 240 250 260 270 280 290
HPV-45 ACCCTACTAAGTTTAAGCAGTATAGTAGACATGTGGAGGAATATGATTTACAGTTTATTTTTCAGTTGTGCACTATTACTTTAACTGCAGAGGTTAT
4 ACCCTACTAAGTTTAAGCAGTATAGTAGACATGTGGAGGAATATGATTTACAGTTTATTTTTCAGTTGTGCACTATTACTTTAACTGCAGAGGTTAT
300 310 320 330 340 350 360 370
HPV-45 GTCATATATCCATAGTATGAATAGTAGTATATTAGAAAATTGGAATTTTGGTGTCCCTCCACCACCTACTACAAGTTTGG
4 GTCATATATCCATAGTATGAATAGTAGTATATTAGAAAATTGGAATTTTGGTGTCCCTCCACCACCTACTACAAGTTTG.
1 10 20 30 40 50 60 70 80 90
HPV-45 CCCTTCTCCCAGTGGCTCTATTATTACTTCTGATTCTCAATTATTTAATAAGCCATATTGGTTACATAAGGCCCAGGGCCATAACAATGGTATTTGT
5 CCCTTCTCCCAGTGGCTCTATTATTACTTCTGATTCTCAATTATTTAATAAGCCATATTGGTTACATAAGGCCCAGGGCCATAACAATGGTATTTGT
100 110 120 130 140 150 160 170 180 190
HPV-45 TGGCATAATCAGTTGTTTGTTACTGTAGTGGACACTACCCGCAGTACTAATTTAACATTATGTGCCTCTACACAAAATTCTGTGCCAAGTACATATG
5 TGGCATAATCAGTTGTTTGTTACTGTAGTGGACACTACCCGCAGTACTAATTTAACATTATGTGCCTCTACACAAAATTCTGTGCCAAGTACATATG
200 210 220 230 240 250 260 270 280 290
HPV-45 ACCCTACTAAGTTTAAGCAGTATAGTAGACATGTGGAGGAATATGATTTACAGTTTATTTTTCAGTTGTGCACTATTACTTTAACTGCAGAGGTTAT
5 ACCCTACTAAGTTTAAGCAGTATAGTAGACATGTGGAGGAATATGATTTACAGTTTATTTTTCAGTTGTGCACTATTACTTTAACTGCAGAGGTTAT
300 310 320 330 340 350 360 370
HPV-45 GTCATATATCCATAGTATGAATAGTAGTATATTAGAAAATTGGAATTTTGGTGTCCCTCCACCACCTACTACAAGTTTGG
5 GTCATATATCCATAGTATGAATAGTAGTATATTAGAAAATTGGAATTTTGGTGTCCCTCCACCACCTACTACAA......

```
         1         10        20        30        40        50        60        70        80        90
HPV-52 1 CCTGTCTCTAAGGTTGTAAGCACTGATGAGTATGTGTCTCGCACAAGCATCTATTATTATGCAGGCAGTTCTCGATTACTAACAGTAGGACATCCCT
         CCTGTCTCTAAGGTTGTAAGCACTGATGAGTATGTGTCTCGCACAAGCATCTATTATTATGCAGGCAGTTCTCGATTACTAACAGTAGGACATCCCT

         100       110       120       130       140       150       160       170       180       190
HPV-52 1 ATTTTTCTATTAAAAACACCAGTAGTGGTAATGGTAAAAAAGTTTTAGTTCCCAAGGTGTCTGGCCTGCAATACAGGGTATTTAGAATTAAATTGCC
         ATTTTTCTATTAAAAACACCAGTAGTGGTAATGGTAAAAAAGTTTTAGTTCCCAAGGTGTCTGGCCTGCAATACAGGGTATTTAGAATTAAATTGCC

            200       210       220       230       240       250       260       270       280       290
HPV-52 1 GGACCCTAATAAATTTGGTTTTCCAGATACATCTTTTTATAACCCAGAAACCCAAAGGTTGGTGTGGGCCTGTACAGGCTTGGAAATTGGTAGGGGA
         GGACCCTAATAAATTTGGTTTTCCAGATACATCTTTTTATAACCCAGAAACCCAAAGGTTGGTGTGGGCCTGTACAGGCTTGGAAATTGGTAGGGGA

               300       310
HPV-52 1 CAGCCTTTAGGTGTGGGTATTAGTGGG
         CAGCCTTTAGGTGTGGGTATTAGTGGG

         1         10        20        30        40        50        60        70        80        90
HPV-52 2 CCTGTCTCTAAGGTTGTAAGCACTGATGAGTATGTGTCTCGCACAAGCATCTATTATTATGCAGGCAGTTCTCGATTACTAACAGTAGGACATCCCT
         CCTGTCTCTAAGGTTGTAAGCACTGATGAGTATGTGTCTCGCACAAGCATCTATTATTATGCAGGCAGTTCTCGATTACTAACAGTAGGACATCCCT

         100       110       120       130       140       150       160       170       180       190
HPV-52 2 ATTTTTCTATTAAAAACACCAGTAGTGGTAATGGTAAAAAAGTTTTAGTTCCCAAGGTGTCTGGCCTGCAATACAGGGTATTTAGAATTAAATTGCC
         ATTTTTCTATTAAAAACACCAGTAGTGGTAATGGTAAAAAAGTTTTAGTTCCCAAGGTGTCTGGCCTGCAATACAGGGTATTTAGAATTAAATTGCC

            200       210       220       230       240       250       260       270       280       290
HPV-52 2 GGACCCTAATAAATTTGGTTTTCCAGATACATCTTTTTATAACCCAGAAACCCAAAGGTTGGTGTGGGCCTGTACAGGCTTGGAAATTGGTAGGGGA
         GGACCCTAATAAATTTGGTTTTCCAGATACATCTTTTTATAACCCAGAAACCCAAAGGTTGGTGTGGGCCTGTACAGGCTTGGAAATTGGTAGGGGA

               300       310
HPV-52 2 CAGCCTTTAGGTGTGGGTATTAGTGGG
         CAGCCTTTAGGTGTGGGTATTAGTGGG

                 1        10        20        30        40        50        60        70        80
HPV-52 3 ........CCTGTCTCTAAGGTTGTAAGCACTGATGAGTATGTGTCTCGCACAAGCATCTATTATTATGCAGGCAGTTCTCGATTACTAACAGTAGG
         CTCCTGTGCCTGTGTCTAAGGTTGTAAGCACTGATGAGTATGTGTCTCGCACAAGCATCTATTATTATGCAGGCAGTTCTCGATTACTAACAGTAGG

         90        100       110       120       130       140       150       160       170       180
HPV-52 3 ACATCCCTATTTTTCTATTAAAAACACCAGTAGTGGTAATGGTAAAAAAGTTTTAGTTCCCAAGGTGTCTGGCCTGCAATACAGGGTATTTAGAATT
         ACATCCCTATTTTTCTATTAAAAACACCAGTAGTGGTAATGGTAAAAAAGTTTTAGTTCCCAAGGTGTCTGGCCTGCAATACAGGGTATTTAGAATT

            190       200       210       220       230       240       250       260       270       280
HPV-52 3 AAATTGCCGGACCCTAATAAATTTGGTTTTCCAGATACATCTTTTTATAACCCAGAAACCCAAAGGTTGGTGTGGGCCTGTACAGGCTTGGAAATTG
         AAATTGCCGGACCCTAATAAATTTGGTTTTCCAGATACATCTTTTTATAACCCAGAAACCCAAAGGTTGGTGTGGGCCTGTACAGGCTTGGAAATTG

               290       300       310
HPV-52 3 GTAGGGGACAGCCTTTAGGTGTGGGTATTAGTGGG
         GTAGGGGACAGCCTTTAGGTGTGGGTATTAGTGGG
```

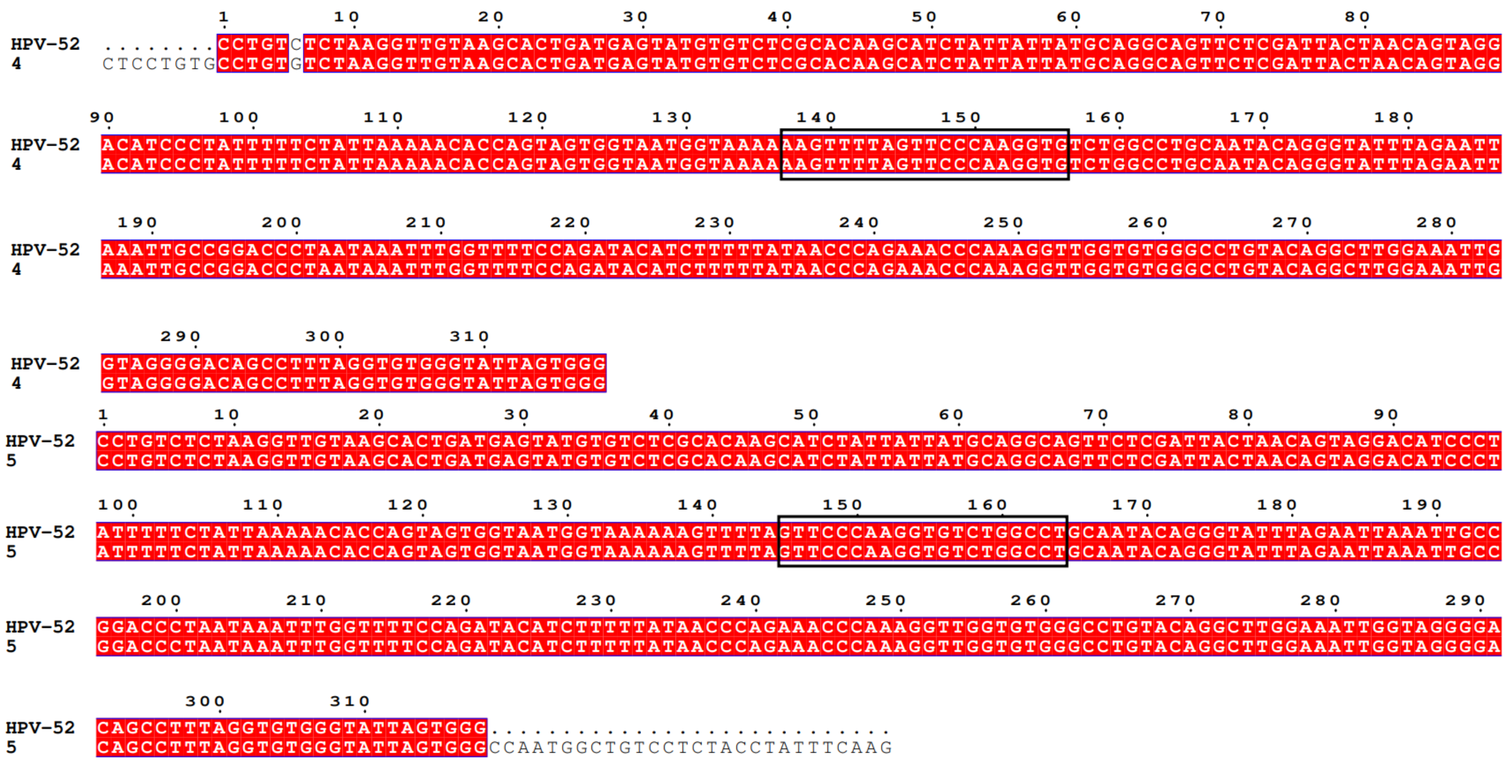
1 10 20 30 40 50 60 70 80
HPV-52 ........CCTGTCTCTAAGGTTGTAAGCACTGATGAGTATGTGTCTCGCACAAGCATCTATTATTATGCAGGCAGTTCTCGATTACTAACAGTAGG
4 CTCCTGTGCCTGTGTCTAAGGTTGTAAGCACTGATGAGTATGTGTCTCGCACAAGCATCTATTATTATGCAGGCAGTTCTCGATTACTAACAGTAGG
90 100 110 120 130 140 150 160 170 180
HPV-52 ACATCCCTATTTTTCTATTAAAAACACCAGTAGTGGTAATGGTAAAAAAGTTTTAGTTCCCAAGGTGTCTGGCCTGCAATACAGGGTATTTAGAATT
4 ACATCCCTATTTTTCTATTAAAAACACCAGTAGTGGTAATGGTAAAAAAGTTTTAGTTCCCAAGGTGTCTGGCCTGCAATACAGGGTATTTAGAATT
190 200 210 220 230 240 250 260 270 280
HPV-52 AAATTGCCGGACCCTAATAAATTTGGTTTTCCAGATACATCTTTTTATAACCCAGAAACCCAAAGGTTGGTGTGGGCCTGTACAGGCTTGGAAATTG
4 AAATTGCCGGACCCTAATAAATTTGGTTTTCCAGATACATCTTTTTATAACCCAGAAACCCAAAGGTTGGTGTGGGCCTGTACAGGCTTGGAAATTG
290 300 310
HPV-52 GTAGGGGACAGCCTTTAGGTGTGGGTATTAGTGGG
4 GTAGGGGACAGCCTTTAGGTGTGGGTATTAGTGGG
1 10 20 30 40 50 60 70 80 90
HPV-52 CCTGTCTCTAAGGTTGTAAGCACTGATGAGTATGTGTCTCGCACAAGCATCTATTATTATGCAGGCAGTTCTCGATTACTAACAGTAGGACATCCCT
5 CCTGTCTCTAAGGTTGTAAGCACTGATGAGTATGTGTCTCGCACAAGCATCTATTATTATGCAGGCAGTTCTCGATTACTAACAGTAGGACATCCCT
100 110 120 130 140 150 160 170 180 190
HPV-52 ATTTTTCTATTAAAAACACCAGTAGTGGTAATGGTAAAAAAGTTTTAGTTCCCAAGGTGTCTGGCCTGCAATACAGGGTATTTAGAATTAAATTGCC
5 ATTTTTCTATTAAAAACACCAGTAGTGGTAATGGTAAAAAAGTTTTAGTTCCCAAGGTGTCTGGCCTGCAATACAGGGTATTTAGAATTAAATTGCC
200 210 220 230 240 250 260 270 280 290
HPV-52 GGACCCTAATAAATTTGGTTTTCCAGATACATCTTTTTATAACCCAGAAACCCAAAGGTTGGTGTGGGCCTGTACAGGCTTGGAAATTGGTAGGGGA
5 GGACCCTAATAAATTTGGTTTTCCAGATACATCTTTTTATAACCCAGAAACCCAAAGGTTGGTGTGGGCCTGTACAGGCTTGGAAATTGGTAGGGGA
300 310
HPV-52 CAGCCTTTAGGTGTGGGTATTAGTGGG..............................
5 CAGCCTTTAGGTGTGGGTATTAGTGGGCCAATGGCTGTCCTCTACCTATTTCAAG

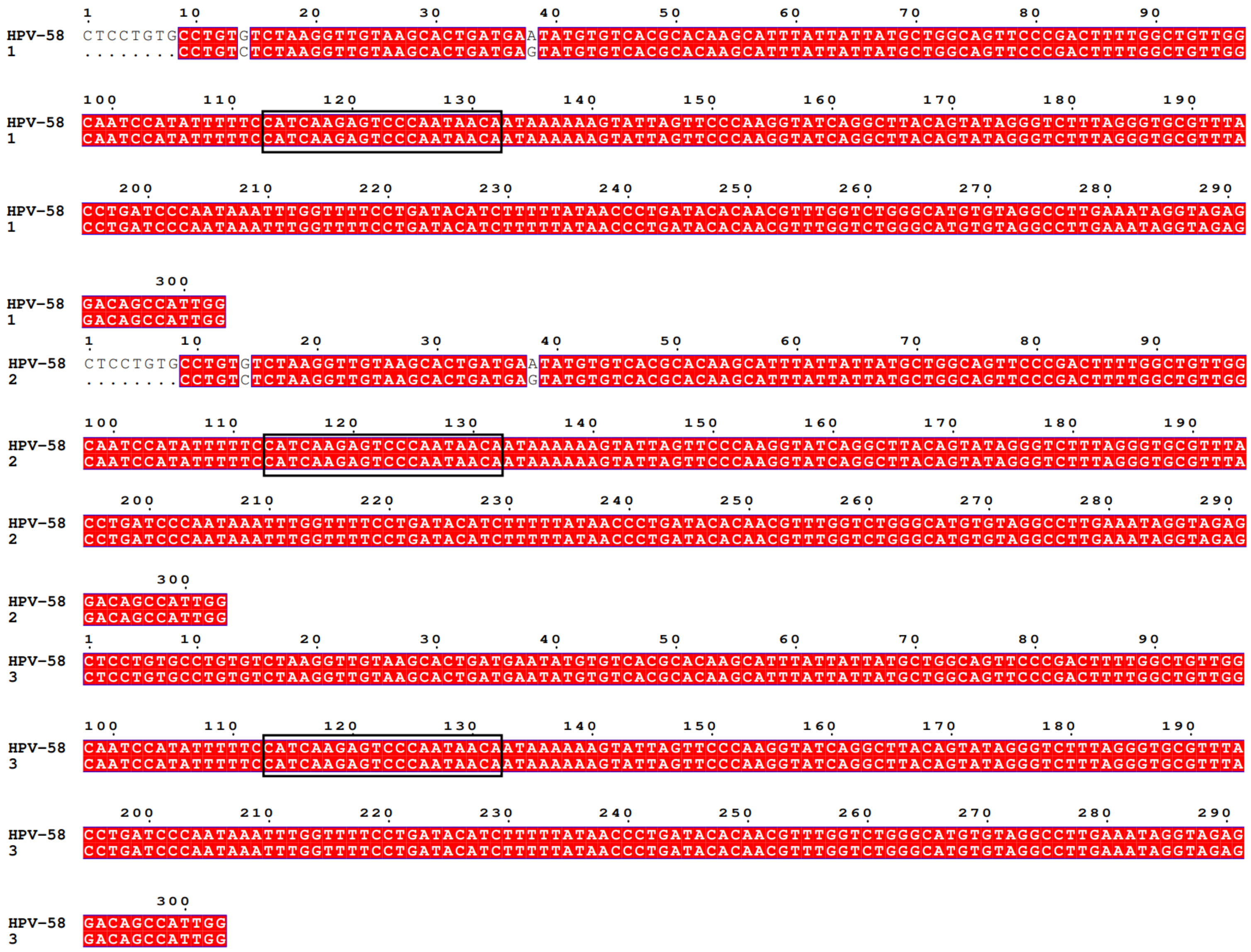
HPV-58 CTCCTGTGCCTGTGTCTAAGGTTGTAAGCACTGATGAATATGTGTCACGCACAAGCATTTATTATTATGCTGGCAGTTCCCGACTTTTGGCTGTTGG
1 ........CCTGTCTCTAAGGTTGTAAGCACTGATGAGTATGTGTCACGCACAAGCATTTATTATTATGCTGGCAGTTCCCGACTTTTGGCTGTTGG
HPV-58 CAATCCATATTTTTCCATCAAGAGTCCCAATAACAATAAAAAAGTATTAGTTCCCAAGGTATCAGGCTTACAGTATAGGGTCTTTAGGGTGCGTTTA
1 CAATCCATATTTTTCCATCAAGAGTCCCAATAACAATAAAAAAGTATTAGTTCCCAAGGTATCAGGCTTACAGTATAGGGTCTTTAGGGTGCGTTTA
HPV-58 CCTGATCCCAATAAATTTGGTTTTCCTGATACATCTTTTTATAACCCTGATACACAACGTTTGGTCTGGGCATGTGTAGGCCTTGAAATAGGTAGAG
1 CCTGATCCCAATAAATTTGGTTTTCCTGATACATCTTTTTATAACCCTGATACACAACGTTTGGTCTGGGCATGTGTAGGCCTTGAAATAGGTAGAG
HPV-58 GACAGCCATTGG
1 GACAGCCATTGG
HPV-58 CTCCTGTGCCTGTGTCTAAGGTTGTAAGCACTGATGAATATGTGTCACGCACAAGCATTTATTATTATGCTGGCAGTTCCCGACTTTTGGCTGTTGG
2 ........CCTGTCTCTAAGGTTGTAAGCACTGATGAGTATGTGTCACGCACAAGCATTTATTATTATGCTGGCAGTTCCCGACTTTTGGCTGTTGG
HPV-58 CAATCCATATTTTTCCATCAAGAGTCCCAATAACAATAAAAAAGTATTAGTTCCCAAGGTATCAGGCTTACAGTATAGGGTCTTTAGGGTGCGTTTA
2 CAATCCATATTTTTCCATCAAGAGTCCCAATAACAATAAAAAAGTATTAGTTCCCAAGGTATCAGGCTTACAGTATAGGGTCTTTAGGGTGCGTTTA
HPV-58 CCTGATCCCAATAAATTTGGTTTTCCTGATACATCTTTTTATAACCCTGATACACAACGTTTGGTCTGGGCATGTGTAGGCCTTGAAATAGGTAGAG
2 CCTGATCCCAATAAATTTGGTTTTCCTGATACATCTTTTTATAACCCTGATACACAACGTTTGGTCTGGGCATGTGTAGGCCTTGAAATAGGTAGAG
HPV-58 GACAGCCATTGG
2 GACAGCCATTGG
HPV-58 CTCCTGTGCCTGTGTCTAAGGTTGTAAGCACTGATGAATATGTGTCACGCACAAGCATTTATTATTATGCTGGCAGTTCCCGACTTTTGGCTGTTGG
3 CTCCTGTGCCTGTGTCTAAGGTTGTAAGCACTGATGAATATGTGTCACGCACAAGCATTTATTATTATGCTGGCAGTTCCCGACTTTTGGCTGTTGG
HPV-58 CAATCCATATTTTTCCATCAAGAGTCCCAATAACAATAAAAAAGTATTAGTTCCCAAGGTATCAGGCTTACAGTATAGGGTCTTTAGGGTGCGTTTA
3 CAATCCATATTTTTCCATCAAGAGTCCCAATAACAATAAAAAAGTATTAGTTCCCAAGGTATCAGGCTTACAGTATAGGGTCTTTAGGGTGCGTTTA
HPV-58 CCTGATCCCAATAAATTTGGTTTTCCTGATACATCTTTTTATAACCCTGATACACAACGTTTGGTCTGGGCATGTGTAGGCCTTGAAATAGGTAGAG
3 CCTGATCCCAATAAATTTGGTTTTCCTGATACATCTTTTTATAACCCTGATACACAACGTTTGGTCTGGGCATGTGTAGGCCTTGAAATAGGTAGAG
HPV-58 GACAGCCATTGG
3 GACAGCCATTGG

```
        1         10        20        30        40        50        60        70        80        90
HPV-58  CTCCTGTGCCTGTGTCTAAGGTTGTAAGCACTGATGAATATGTGTCACGCACAAGCATTTATTATTATGCTGGCAGTTCCCGACTTTTGGCTGTTGG
4       CTCCTGTGCCTGTGTCTAAGGTTGTAAGCACTGATGAATATGTGTCACGCACAAGCATTTATTATTATGCTGGCAGTTCCCGACTTTTGGCTGTTGG

        100       110       120       130       140       150       160       170       180       190
HPV-58  CAATCCATATTTTTCCATCAAGAGTCCCAATAACAATAAAAAAGTATTAGTTCCCAAGGTATCAGGCTTACAGTATAGGGTCTTTAGGGTGCGTTTA
4       CAATCCATATTTTTCCATCAAGAGTCCCAATAACAATAAAAAAGTATTAGTTCCCAAGGTATCAGGCTTACAGTATAGGGTCTTTAGGGTGCGTTTA

           200       210       220       230       240       250       260       270       280       290
HPV-58  CCTGATCCCAATAAATTTGGTTTTCCTGATACATCTTTTTATAACCCTGATACACAACGTTTGGTCTGGGCATGTGTAGGCCTTGAAATAGGTAGAG
4       CCTGATCCCAATAAATTTGGTTTTCCTGATACATCTTTTTATAACCCTGATACACAACGTTTGGTCTGGGCATGTGTAGGCCTTGAAATAGGTAGAG

              300
HPV-58  GACAGCCATTGG
4       GACAGCCATTGG
```

```
        1         10        20        30        40        50        60        70        80        90
HPV-58  CTCCTGTGCCTGTGTCTAAGGTTGTAAGCACTGATGAATATGTGTCACGCACAAGCATTTATTATTATGCTGGCAGTTCCCGACTTTTGGCTGTTGG
5       CTCCTGTGCCTGTGTCTAAGGTTGTAAGCACTGATGAGTATGTGTCACGCACAAGCATTTATTATTATGCTGGCAGTTCCCGACTTTTGGCTGTTGG

        100       110       120       130       140       150       160       170       180       190
HPV-58  CAATCCATATTTTTCCATCAAGAGTCCCAATAACAATAAAAAAGTATTAGTTCCCAAGGTATCAGGCTTACAGTATAGGGTCTTTAGGGTGCGTTTA
5       CAATCCATATTTTTCCATCAAGAGTCCCAATAACAATAAAAAAGTATTAGTTCCCAAGGTATCAGGCTTACAGTATAGGGTCTTTAGGGTGCGTTTA

           200       210       220       230       240       250       260       270       280       290
HPV-58  CCTGATCCCAATAAATTTGGTTTTCCTGATACATCTTTTTATAACCCTGATACACAACGTTTGGTCTGGGCATGTGTAGGCCTTGAAATAGGTAGAG
5       CCTGATCCCAATAAATTTGGTTTTCCTGATACATCTTTTTATAACCCTGATACACAACGTTTGGTCTGGGCATGTGTAGGCCTTGAAATAGGTAGAG

              300
HPV-58  GACAGCCATTGG
5       GACAGCCATTGG
```
